# Allogeneic bone marrow-derived mesenchymal stem cells in the aging kidney: secondary results of a Parkinson’s disease clinical trial

**DOI:** 10.1186/s13287-025-04577-y

**Published:** 2025-09-24

**Authors:** Juan D. Martinez-Lemus, Donald A. Molony, Jessika Suescun, Emily Tharp, Tia S. Thomas, Charles Green, Chiamaka Onuigbo, Robert Ritter, Mya C. Schiess

**Affiliations:** 1 Movement Disorders Division, Department of Neurology, McGovern Medical School at UTHealth Houston, University of Texas, 6431 Fannin Street, Houston, TX USA; 2 Division of Renal Diseases & Hypertension, Department of Medicine, McGovern Medical School at UTHealth Houston, Houston, TX 77030 USA; 3 Translational Research Institute, The Queensland University of Technology, Brisbane, QLD Australia; 4 Institute for Stroke and Cerebrovascular Diseases, McGovern Medical School at UTHealth Houston, Houston, TX USA; 5 Department of Pediatrics, Center for Clinical Research & Evidence-Based Medicine, McGovern Medical School at UTHealth, Houston, TX USA

**Keywords:** Stem cells, Clinical study, Estimated glomerular filtration rate, Glomerular filtration rate, Inflammation

## Abstract

**Background:**

Kidney function declines with age, largely due to chronic low-grade inflammation. Mesenchymal stem cells (MSCs) have demonstrated immunomodulatory effects in certain immune-mediated kidney diseases, but their role in preserving renal function in aging individuals without chronic kidney disease (CKD) remains unclear. This study presents secondary outcome findings from a randomized clinical trial in Parkinson’s disease (PD), evaluating the impact of allogeneic human bone marrow-derived MSCs (allo-hMSCs) on kidney function in an aging population with PD with preserved renal function.

**Methods:**

Subjects with PD aged 50–79 years with baseline estimated glomerular filtration rate (eGFR) > 60 mL/min/1.73 m^2^ were randomized to receive either three allo-hMSC infusions, one placebo followed by two allo-hMSC infusions, or three placebo infusions at 18-week intervals. Kidney function was assessed using eGFR, serum creatinine (SCr), and blood urea nitrogen (BUN) at baseline, 9 weeks after the first two infusions, and at weeks 40 and 88. eGFR was calculated using the 2021 CKD-EPI equation. A Bayesian modeling approach was used to estimate posterior probabilities (PP) of treatment effects.

**Results:**

Of 45 randomized patients, 44 were analyzed; 43 completed infusions, and 40 completed the 88-week follow-up. The three-infusion group (N = 16) showed an average annual eGFR increase of 3.29 mL/min/1.73 m^2^, versus declines of –1.46 and –2.92 in the two-infusion (N = 14) and placebo (N = 15) groups. SCr decreased by –0.12 mg/dL at both weeks 40 (PP: 93.9%) and 88 (PP: 86.2%) in the three-infusion group versus placebo, with no significant SCr differences between the two-infusion and placebo groups. BUN levels did not differ significantly between treatment and placebo groups.

**Conclusion:**

In older adults with PD and preserved kidney function, repeated allo-hMSC infusions were associated with improved kidney function measures. While promising, these findings are preliminary and may be specific to PD. Further studies are needed to assess potential benefits in the broader aging population.

**Trial Registration:**

ClinicalTrials.Gov. NCT04506073. November 09, 2020. https://clinicaltrials.gov/study/NCT04506073

**Supplementary Information:**

The online version contains supplementary material available at 10.1186/s13287-025-04577-y.

## Introduction

The aging population is a growing demographic challenge, with individuals aged 65 and over expected to comprise 23% of the population by 2054 [1]. This demographic shift is accompanied by an increasing prevalence of age-related neurodegenerative diseases, particularly Parkinson’s disease (PD), which affects approximately 1% of individuals over age 65 and more than 5% of those over 85 [2]. In parallel, chronic kidney disease (CKD), present in 39.4% of individuals over age 60 [3], has also been independently associated with higher PD prevalence [4]. While this link was initially attributed to uremic toxicity in the basal ganglia [5], emerging evidence supports the existence of a"kidney–brain axis"[6] in which impaired renal clearance of circulating alpha-synuclein may contribute to its accumulation and propagation in the brain, ultimately leading to dopaminergic neurodegeneration and the development of PD motor and non-motor symptoms [7].

More recently, researchers have proposed a potential reverse relationship: that PD itself, particularly its autonomic features, may negatively impact kidney function. Cardiovascular and urinary autonomic dysfunction commonly seen in PD, such as orthostatic hypotension, supine hypertension, and neurogenic bladder, may impair renal perfusion, increase the risk of infection, and contribute to kidney injury [8]. Similar patterns in related synucleinopathies like pure autonomic failure, where patients show significantly lower eGFR, support this possible link [9]. However, the absence of large, prospective studies following PD patients with preserved renal function means there is no clear evidence that PD independently increases the risk of CKD, and it is not currently recognized as a formal risk factor. Aging itself, however, is associated with structural and functional changes in the kidneys, including glomerular senescence and tubular atrophy [10, 11]. These changes contribute to an annual glomerular filtration rate (GFR) decline of approximately −1 mL/min/1.73 m^2^ [12, 13]. As a result, more than half of individuals over 70 years have an eGFR of less than 60 mL/min/1.73 m^2^, a level of function that is defined as CKD stage [14].

Renal senescence is characterized by multiple interconnected mechanisms, including telomere shortening, cellular senescence and apoptosis, mitochondrial and lysosomal dysfunction, changes in the sirtuin and Klotho signaling pathways, degeneration of the renin–angiotensin–aldosterone axis, and chronic inflammation [15, 16]. Recent findings in animal models suggest that chronic inflammation serves as an initial trigger of kidney aging, with Tumor Necrosis Factor (TNF)-α and interleukin (IL)−6 potentially dysregulating the pentose phosphate pathway and leading to increased oxidative stress, a known driver of cellular aging and damage [17]. This hypothesis is further supported by longitudinal studies demonstrating that both the levels and the rate of increase of TNFα and IL-6 correlate with kidney dysfunction in aging [18–23]. While inflammation plays a key role, it is not the only mechanism contributing to renal aging. Additional pathways, including the restoration of sirtuin activity, modulation of WNT/β-catenin signaling, and activation of mitogen-activated protein kinase (MAPK) pathways, have also been implicated in kidney aging and represent important therapeutic targets in regenerative research [24].

In addition to the physiological changes associated with aging, the increasing prevalence of noncommunicable chronic diseases contributes to the rising incidence of CKD, affecting 27.5% of older adults with hypertension [25] and 40% of those with diabetes [26]. However, aging itself has been proposed as an independent contributor to kidney dysfunction in hypertensive patients [27], potentially accelerating the decline in eGFR in individuals already at risk due to hypertension and other chronic diseases [28]. No existing therapies effectively halt the natural progression of kidney aging or the unfavorable course of age-related kidney diseases. This unmet need has driven research into regenerative medicine, including the potential application of stem cell-based therapies to mitigate age-related renal decline and restore kidney function [29].

Mesenchymal stem cells (MSCs) are multipotent progenitor cells derived from various tissues, including bone marrow, adipose tissue, umbilical cord, and peripheral blood. These cells exhibit a diverse range of therapeutic properties, including anti-apoptotic, antioxidant, anti-inflammatory, antifibrotic, and immunomodulatory effects [30–33]. They have also been proven to be minimally immunogenic [34], have low tumorigenesis risk [35], are easy to procure and expand, and raise few ethical concerns compared to other stem cell products, making them highly promising candidates for regenerative medicine and a wide array of therapeutic applications [29]. MSCs have the ability to transition between a proinflammatory (MSC1) and an anti-inflammatory phenotype (MSC2), a process primarily driven by elevated levels of IFN-γ, IL-6, and TNF-α [36, 37]. This phenotypic plasticity may influence key pathways involved in aging beyond inflammation, including sirtuin regulation, WNT/β-catenin modulation, and MAPK signaling, thereby offering a multifaceted approach to targeting renal senescence and preserving kidney function in older adults [24].

These therapeutic properties have translated into early-phase clinical trials, showing the potential of MSC-based therapies in treating acute kidney injury, CKD, diabetic nephropathy, atherosclerotic renovascular disease, and lupus nephritis [38], suggesting that MSCs may help manage established kidney diseases primarily by slowing or, in rare cases, halting the decline in kidney function. However, to date, no interventional studies have evaluated the impact of MSCs in individuals with normal kidney function and no preexisting renal comorbidities.

In this context, we report findings from a Phase 2 clinical trial of allogeneic bone marrow-derived MSCs (allo-hMSC) for PD, demonstrating longitudinal improvements in eGFR and serum creatinine (SCr) levels. These results are based on data from comprehensive metabolic panels used as part of the trial’s safety assessment in patients with PD who had normal kidney function at trial enrollment (eGFR > 60 mL/min/1.73 m^2^).

## Methods

### Study design

We conducted a single-center, Phase 2 randomized, double-blind, placebo-controlled clinical trial at the University of Texas Health Science Center at Houston (UTHealth), United States, from November 2020 to July 2023, to evaluate the potential of allo-hMSCs in reducing symptoms in patients with mild-to-moderate PD (NCT04506073)^in press^. The study was approved by the Institutional Review Board at UTHealth (IRB No. HSC-MS-20–0150) and by the U.S. Food and Drug Administration (IND No. 16756). The treatment phase included three arms, each receiving a different combination of three infusions administered every 18 weeks over a total treatment period of 36 weeks, followed by a final assessment at week 88, 52 weeks after the last infusion. The full protocol is available in Supplement 1.

### Participants

Patients aged 50 to 79 with mild-to-moderate PD were included. Relevant exclusion criteria for this report included an eGFR < 45 mL/min/m^2^, a Body Mass Index (BMI) ≥ 40, active autoimmune disorders, or a previous history of stem cell treatment. All participants provided written informed consent before undergoing trial-specific screening tests and evaluations. Details of the inclusion and exclusion criteria are provided in Supplement 1.

### Procedures

MSCs were derived from bone marrow aspirated from a single healthy donor, screened for infectious diseases, and HLA-typed [39]. Cells were expanded and validated under cGMP conditions at Baylor College of Medicine (Center for Cell and Gene Therapy) per International Society for Cell & Gene Therapy (ISCT) criteria, suspended in Plasma Lyte A with 5% Flexbumin and 10% DMSO, and then cryopreserved in treatment-ready doses. The placebo was visually identical but contained only 5% Buminate without allo-hMSCs. Masking was maintained using opaque covers on infusion bags and IV lines.

Eligible patients were randomly assigned in a 1:1:1 ratio to one of the three treatment groups: a) three infusions of 10 × 10^6^ allo-hMSCs/kg, b) placebo followed by two infusions of 10 × 10^6^ allo-hMSCs/kg, or c) three infusions of placebo (5% albumin solution). The dosage was based on our Phase 1 clinical trial, in which 10 × 10⁶ allo-hMSCs/kg (the highest dose tested) was found to be safe [39]. Initially, dosing intervals were planned every 12 weeks, reflecting Phase 1 observations that motor improvements began to wane after this period. However, due to supply constraints related to the COVID-19 pandemic at the time of study initiation in February 2020, the interval was extended to 18 weeks. All patients ultimately received infusions on this 18-week schedule. Stratified block randomization based on the Movement Disorders Society–Unified Parkinson’s Disease Rating Scale (MDS-UPDRS) subscale III scores was used to evenly distribute baseline motor symptom severity across treatment groups and strengthen the validity of comparisons.

Participants received allo-hMSC or placebo infusions at weeks 1, 18, and 36. Post-infusion follow-up assessments were conducted at weeks 9, 27, and 40, with a final follow-up visit at week 88. At baseline (prior to the first infusion) and during each post-infusion follow-up, all participants underwent comprehensive safety evaluations, including medical history, PD-specific assessments, biomarker measurements, and safety laboratory testing.

### Outcomes

Kidney function was assessed using SCr measured by an enzymatic assay with creatininase and colorimetric detection, and blood urea nitrogen (BUN), measured by the urease–glutamate dehydrogenase method, both performed in the laboratory of Memorial Hermann–Texas Medical Center. eGFR (measured in mL/min/1.73 m^2^) was calculated from SCr using the 2021 CKD-EPI Eq. [40]. Proteinuria was assessed using a dipstick method on a first-morning urine sample collected on the day of the visit.

### Statistical analyses

Bayesian methods were used to estimate the probability of the alternative hypothesis based on the observed data, capturing the posterior distribution and assessing the probability of the true parameter value within a defined range. SCr, BUN, and eGFR levels were compared across treatment groups after adjusting for stratification at weeks 40 and 88. Priors for regression coefficients followed a ~ Normal (µ = 0, σ2 = 1 × 10^3^) distribution, and level one error variances followed ~ Student-T (µ = 0, df = 3, σ2 = 1 × 10^2^). Furthermore, multilevel generalized linear modeling (GLM) with random intercepts was used to assess longitudinal changes in SCr, BUN, and eGFR. This approach accounted for repeated measurements within participants over time and adjusted for stratification and treatment group. The GLM results, expressed as daily rates of change, were linearly extrapolated by multiplying by 365 days to estimate the annual rate of change, providing clinically meaningful insight into year-over-year changes in kidney function. Additionally, statistical interactions between time and treatment group were tested to examine the differential rates of change by treatment group. Priors for regression coefficients followed a ~ Normal (µ = 0, σ2 = 1 × 10^2^) distribution, and level one error variances were ~ Student-T (µ = 0, df = 3, σ2 = 1 × 10). Level two variances followed Gelman’s recommendations [41]. Analyses applied intention-to-treat principles, addressing missingness through joint modeling of observed and missing data, a robust approach to ignorable missingness (MCAR and MAR) [42]. Data were analyzed using R version 4.2.0.

## Results

### Number of participants recruited and included in the analysis

**Fig. 1:**
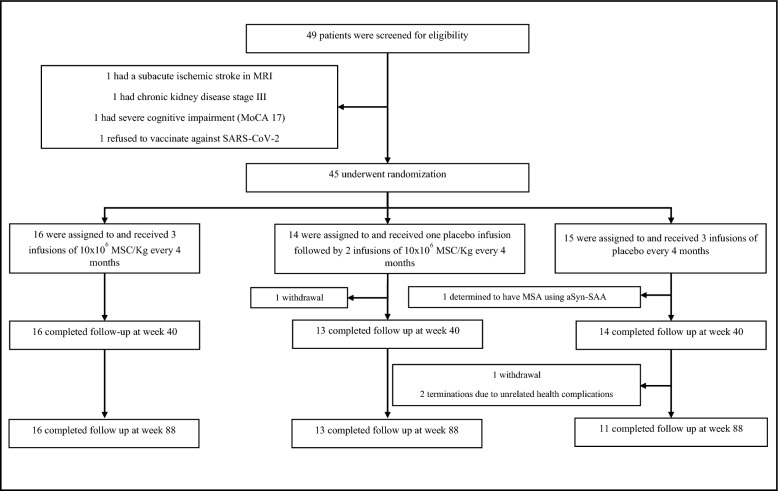
Screening, Randomization, and Follow-Up. MoCA: Montreal Cognitive Assessment. SARS-CoV-2: Severe Acute Respiratory Syndrome-Coronavirus 2. allo-hMSC: Human Allogeneic Bone Marrow-Derived Mesenchymal Stem Cells. MSA: Multiple System Atrophy. aSyn-SAA: Alpha-Synuclein Seed Amplification Assay

A total of 45 PD patients were enrolled and randomly assigned to one of three groups: 16 received three allo-hMSC infusions, 14 received two allo-hMSC infusions, and 15 were allocated to the placebo group. All participants received their infusions at 18-week intervals, administered within a one-week treatment window. During the trial, one participant was clinically diagnosed with Multiple System Atrophy, confirmed by an alpha-synuclein seed amplification test, and was excluded from the final analyses [43]. Of the remaining 44 participants, 43 completed all three infusions and were evaluated for safety and clinical outcomes, including SCr and BUN levels at week 40. By the end of the study at week 88, two participants discontinued early due to severe adverse events deemed unrelated to the treatment, and one additional participant withdrew voluntarily, resulting in 40 patients completing the 88-week follow-up (Fig. 1).

### Baseline characteristics

The mean age of the study cohort was 66.5 years (SD = 7.2), with 22.2% of participants being female. Among the cohort, 35.6% had chronic hypertension, 4.4% had diabetes, and 24.4% had hyperlipidemia. On entry into the study, all subjects had documented and stable control of their blood pressures and an absent antecedent history of CKD or progressive renal decline due to hypertension or medications. There were no differences in the prevalence of angiotensin receptor blocker (ARB) or angiotensin-converting enzyme inhibitor (ACEi) use between groups. No participant was on a chronically nephrotoxic medication, and past Non-Steroidal Anti-Inflammatory Drugs (NSAID) use was balanced across groups (Table 1). The mean BMI was 27.2 (SD = 4.27). Baseline kidney function assessments showed a mean SCr levels of 0.98 mg/dL (SD, 0.19), a mean BUN levels of 19.38 mg/dL (SD, 5.05), and a mean eGFR of 81.12 mL/min/1.73 m^2^ (SD, 12.74). Proteinuria was detected in 6.8% of participants via urine dipstick testing. Baseline characteristics stratified by treatment arm are presented in Table 1.

**Table 1:** Baseline characteristics of the participants

Variable	Three infusions of alio-hMSC (n = 16)	One placebo followed by two infusions of allo-hMSC (n = 14)	Three infusions of placebo (n = 14)
Age (years)	64.31 ± 8.55	66.86 ± 6.43	69.14 ± 6.04
Sex—Female	4 (25.0%)	3 (21.4%)	3 (21.4%)
Race Asian White	0 (0%) 16 (100%)	0 (0%) 14 (100%)	1 (7.1%) 14 (92.9%)
Ethnicity Hispanic Non-Hispanic	6 (37.5%) 10 (62.5%)	2 (14.3%) 12 (85.7%)	1 (7.1%) 13 (86.7%)
History of NSAID Use Duration—months Duration—years	2 (12.5%) 2 (50%) 2 (50%)	4 (28.6%) 2 (50%) 2 (50%)	4 (28.6%) 1 (25%) 3 (75%)
Current use of ACEi or ARB Use of only ACEi Use of only ARB	5 (31.3%) 1 (6.3%) 4 (25.0%)	2 (14.3%) 1 (7.1%) 1 (7.1%)	4 (28.8%) 4 (28.6%) 0 (0.0%)
History of Smoking	8 (50.0%)	7 (50.0%)	8 (57.1%)
Current Smoker	1 (6.3%)	1 (7.1%)	0 (0%)
Heavy Metal Exposure	0 (0%)	1 (7.1%)	3 (21.4%)
History of Diabetes Mellitus	1 (6.3%)	0 (0%)	1 (7.1%)
History of Hypertension	7 (43.8%)	3 (21.4%)	5 (35.7%)
History of Hyperlipidemia	4 (25.0%)	5 (35.7%)	1 (7.1%)
History of OSA	5 (31.3%)	1 (7.1%)	3 (21.4%)
LED (mg)	711.00 ± 338.74	603.36 ± 241.46	803.14 ± 383.23
Weight (kg)	82.11 ± 15.07	77.88 ± 14.11	81.63 ± 17.4
Height (cm)	171.96 ± 10.96	169.89 ± 8.08	175.96 ± 11.00
BMI	27.63 ± 3.46	26.83 ± 3.29	26.3 ± 5.20
Glucose (mg/dL)	97.63 ± 15.64	92.71 ± 12.34	93.71 ± 11.15
BUN (mg/dL)	18.87 ± 4.39	18.71 ± 5.24	20.79 ± 5.78
Creatinine (mg/dL)	0.96 ± 0.16	1.0 ± 0.19	0.97 ± 0.23
BUN/Creatinine Ratio	20.15 ± 5.63	18.94 ± 4.46	22.05 ± 5.62
eGFR (mL/min/1.73 m^2^)	82.76 ± 9.72	79.81 ± 13.06	79.57 ± 15.74
Proteinuria	2 (12.5%)	0 (0.0%)	1 (7.1%)

Plus-minus values are means ± SD

allo-hMSC: Allogeneic human mesenchymal stem cells; NSAID: Non-steroidal anti-inflammatory drug; ACEi: Angiotensin-Converting Enzyme Inhibitor; ARB: Angiotensin II Receptor Blocker; OSA: Obstructive sleep apnea; LED: Levodopa equivalent dose; BMI: Body mass index; BUN: Blood urea nitrogen; eGFR: Estimated glomerular filtration rate

### Serum creatinine

In the three allo-hMSC infusion group, estimated mean SCr levels were consistently lower compared to the placebo group at both week 40 (mean difference [MD]: –0.12 mg/dL; 95% credible interval [CrI]: –0.28 to 0.03 mg/dL; posterior probability [PP] = 94.2%) and week 88 (MD: –0.12 mg/dL; 95% CrI: –0.34 to 0.10 mg/dL; PP = 86.2%, Table 2). The two allo-hMSC infusion group did not show difference in SCr levels compared to placebo at week 40 (MD: 0.03 mg/dL; 95% CrI: –0.14 to 0.19 mg/dL; PP = 62.3%) and only a slight difference at week 88 (MD: 0.09 mg/dL; 95% CrI: –0.14 to 0.32 mg/dL; PP = 77.0%, Table 2).

**Table 2:** Cross-sectional description of kidney function at 40- and 88-week follow-up

Characteristics	Individual Estimates per Arm						Difference Between Arms					
	Three infusions of allo-hMSC Mean (95%CrI)		One placebo infusion followed by two allo-hMSC Mean (95%CrI)		Three placebo infusions Mean (95%CrI)		Three allo-hMSC vs Placebo infusions Mean Difference (95%CrI) [PP]			Two allo-hMSC vs Placebo infusions Mean Difference (95%CrI) [PP]		
*eGFR*												
Week 40	87.9	(80.4 to 95.3)	71.9	(63.2 to 80.8)	74.7	(67.0 to 82.6)	13.2	(2.4 to 23.7)	99.1%	−2.8	(−14.4 to 8.6)	68.5%
Week 88	88.1	(79.9 to 96.1)	71.1	(62.1 to 80.0)	78.6	(69.5 to 87.9)	9.4	(−2.8 to 21.6)	93.6%	−7.5	(−20.4 to 5.0)	88.2%
*Serum creatinine*												
Week 40	0.9	(0.8 to 1.0)	1.1	(0.9 to 1.2)	1.0	(0.9 to 1.1)	−0.12	(−0.28 to 0.03)	94.2%	0.026	(−0.14 to 0.19)	62.3%
Week 88	0.9	(0.8 to 1.1)	1.1	(1.0 to 1.3)	1.0	(0.9 to 1.2)	−0.12	(−0.34 to 0.10)	86.2%	0.085	(−0.14 to 0.31)	77.0%
*BUN*												
Week 40	18.7	(16.1 to 21.5)	17.1	(13.9 to 20.3)	18.6	(15.7 to 21.4)	0.19	(−3.6 to 4.0)	53.9%	−1.47	(−5.6 to 2.6)	76.4%
Week 88	18.9	(16.2 to 21.5)	18.8	(16.9 to 21.7)	18.2	(15.2 to 21.2)	0.64	(−3.3 to 4.67)	62.7%	0.54	(−3.62 to 4.7)	60.4%

95%CrI, 95% credible Bayesian interval; MDS-UPDRS, Movement Disorders Society-Unified Parkinson's Disease Rating Scale; PP, posterior probability. *PP thresholds are as 70–90% moderate certainty, 90–95% strong certainty, and* > *95% very strong certainty*

**Fig. 2:**
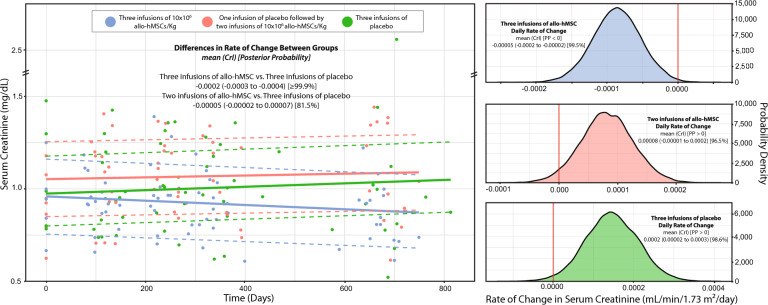
Estimated daily rate of change in serum creatinine levels following treatment with bone marrow-derived mesenchymal stem cells (allo-hMSC) in an aging population using Bayesian modelling analysis. Panel A shows changes in serum creatinine over time across the three treatment groups. Panels B, C, and D display the posterior distributions of the daily rate of change in serum creatinine for patients receiving three allo-hMSC infusions, one placebo followed by two allo-hMSC infusions, and three placebo infusions, respectively. Bayesian modeling was used to estimate the daily rate of change, with credible intervals (CrI) and posterior probabilities (PP) reported to quantify the certainty of the observed effects. A posterior probability (PP) of 50–70% indicates weak certainty, 70–90% moderate certainty, 90–95% strong certainty, and > 95% very strong certainty

Multilevel GLM showed differences in the daily rate of change in SCr levels between the active treatment arms and the placebo group (PP_Two infusions vs. placebo_ = 81.5%; PP_Three infusions vs. placebo_ > 99.9%, Fig. 2). A longitudinal plot illustrating changes in SCr over time by treatment group is presented in Fig. 2A, while Fig. 2B–D display the posterior distributions of the daily rate of change in each group. To improve clinical interpretability, the daily rates in Fig. 2 were linearly extrapolated to estimate the annual rate of change (i.e., daily rate × 365 days). The calculated annual rate of change in SCr was –0.033 mg/dL/year (95% CrI: –0.055 to –0.007; PP < 0 = 99.5%) in the three-infusion group (Fig. 2B), 0.029 mg/dL/year (95% CrI: –0.004 to 0.062; PP > 0 = 96.5%) in the two-infusion group (Fig. 2C), and 0.055 mg/dL/year (95% CrI: 0.007 to 0.10; PP > 0 = 98.6%) in the placebo group (Fig. 2D).

### Serum BUN

Lower BUN concentrations were observed at week 40 in the two-infusion group compared to the placebo group (MD: –1.47 mg/dL; 95% CrI: –5.57 to 2.63; PP = 76.4%), although a minimal difference was observed at week 88. Similarly, marginal differences in BUN levels were found between the three-infusion group and the placebo group at both week 40 and week 88 (Table 2).

There were no significant differences in the daily rate of change in BUN levels between the active treatment arms and placebo, based on multilevel GLM analysis (PP_two infusions vs. placebo_ = 51.9%; PP_three infusions vs. placebo_ = 67%). When extrapolated to a yearly rate, the change in BUN levels—regardless of treatment arm—was –0.365 mg/dL/year (95% CrI: –1.10 to 0.37; PP < 0 = 85.4%; see Supplement 2 A). Additionally, no significant differences were observed in the overall average BUN levels across all timepoints when comparing each active treatment arm to placebo (Supplement 2B).

### Estimated glomerular filtration rate

eGFR was higher in the group receiving three allo-hMSC infusions compared to the placebo group at both week 40 (MD: 13.2 mL/min/1.73 m^2^; 95% CrI: 2.4 to 23.7; PP = 99.1%) and week 88 (MD: 9.4 mL/min/1.73 m^2^; 95% CrI: –2.8 to 21.6; PP = 93.6%, Table 2). In contrast, the group receiving two allo-hMSC infusions showed minimal difference in eGFR compared to placebo at week 40 (MD: –2.8 mL/min/1.73 m^2^; 95% CrI: –14.4 to 8.6 mL/min/1.73 m^2^; PP = 68.5%), while a lower eGFR compared to the placebo was observed at week 88 (MD: –7.5 mL/min/1.73 m^2^; 95% CrI: –20.4 to 5.04 mL/min/1.73 m^2^; PP = 88.2%, Table 2).

**Fig. 3:**
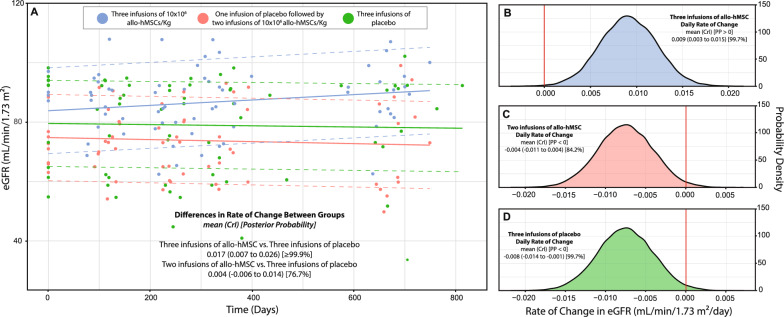
Estimated daily rate of change in glomerular filtration rate (eGRF) following treatment with bone marrow-derived mesenchymal stem cells (allo-hMSC) in an aging population using Bayesian modelling analysis. Panel A shows changes in eGFR over time across the three treatment groups. Panels B, C, and D display the posterior distributions of the daily rate of change in eGFR for patients receiving three allo-hMSC infusions, one placebo followed by two allo-hMSC infusions, and three placebo infusions, respectively. Bayesian modeling was used to estimate the daily rate of change, with credible intervals (CrI) and posterior probabilities (PP) reported to quantify the certainty of the observed effects. A posterior probability (PP) of 50–70% indicates weak certainty, 70–90% moderate certainty, 90–95% strong certainty, and > 95% very strong certainty

Using multilevel GLM, we observed differences in the daily rate of change in eGFR between the treatment groups and the placebo group (PP_Two infusions vs. placebo_ = 76.7%; PP_Three infusions vs. placebo_ > 99.9%). A longitudinal plot illustrating the estimated daily change in eGFR by treatment group is presented in Fig. 3A. The calculated annual rate of change in eGFR was 3.29 mL/min/1.73 m^2^/year (95% CrI: 1.10 to 5.48; PP > 0 = 99.7%) for the three-infusion group (Fig. 3B), –1.46 mL/min/1.73 m^2^/year (95% CrI: –4.02 to 1.46; PP < 0 = 84.2%) for the two-infusion group (Fig. 3C), and –2.92 mL/min/1.73 m^2^/year (95% CrI: –5.11 to –0.37; PP < 0 = 98.5%) for the placebo group (Fig. 3D).

### Proteinuria

At baseline, proteinuria was absent in all participants in the two allo-hMSC group, compared to 13 of 14 (92.9%) in the placebo group and 14 of 16 (87.5%) in the three allo-hMSC group (Table 1). All positive cases were classified as trace, except for one participant in the three allo-hMSC group who had 30 mg (+) proteinuria. By Week 88, all baseline cases of proteinuria had resolved.

## Discussion

The results from this trial, which studied elderly patients with PD without preexisting CKD (eGFR > 60 mL/min/1.73 m^2^ at enrollment), provide clinical evidence suggesting that repeated intravenous infusions of allo-hMSCs may help stabilize and possibly improve kidney function in the aging PD population. These findings are based on a secondary safety evaluation of organ function conducted as part of a Phase 2 clinical trial assessing the immunomodulatory effects of allo-hMSCs in patients with PD (NCT02611167)^in press^. More than 1% of individuals over the age of 65 are diagnosed with PD, a neurodegenerative disorder characterized by dopaminergic neuron loss and driven in part by microglial-mediated neuroinflammation [44]. It is hypothesized that allo-hMSCs reduce brain inflammation by modulating the peripheral immune system through multiple mechanisms [45].

Considering that the average age-related decline in eGFR is approximately −1 mL/min/1.73 m^2^ per year^13^, patients in the placebo arm experienced a more pronounced reduction of –2.92 mL/min/1.73 m^2^/year. In contrast, those receiving active treatment showed a slower decline with two allo-hMSC infusions (–1.46 mL/min/1.73 m^2^/year) and a notable improvement with three infusions (+ 3.29 mL/min/1.73 m^2^/year, Fig. 3). This was accompanied by a slower rise in the rate of change of serum creatinine among patients in the active treatment arms, with a slight decrease of –0.02 mg/dL/year (calculated by totalizing the daily rate of –0.00005 mg/dL/day from the linear model) in the three-infusion group and a modest increase of 0.03 mg/dL/year (0.00008 mg/dL/day) in the two-infusion group, compared to a substantial increase of 0.73 mg/dL/year (0.0002 mg/dL/day) in the placebo group, as shown by the linear modeling in Fig. 2.

Importantly, serum BUN levels remained stable across all groups, suggesting that the observed changes in eGFR and SCr were not attributable to hydration status or nutritional factors. Although this PD-focused study did not include measurements of other freely filtered biomarkers such as Cystatin C, which is unaffected by muscle mass, diet, or sex, prior comparative studies have shown that in individuals with eGFR values between 60 and 89 mL/min/1.73 m^2^, the same range observed in our study population (see Table 1), SCr alone estimates GFR as accurately as Cystatin C or the combined SCr and Cystatin C Eqs. [46].

According to these results, although the two-infusion group showed a slower decline in kidney function relative to placebo, the overall trajectory remained negative, indicating continued deterioration, though to a lesser degree. In contrast, the three-infusion group demonstrated a positive slope, reflecting actual improvement in kidney function over time. This divergence in outcomes between the two and three-infusion groups may reflect a cumulative dose effect or a threshold effect of repeated allo-hMSC administration. While patients were randomized, and measured variables were not different at baseline between groups, PD is a very heterogeneous disease, so there is always a possibility of an unmeasured confounder that could be playing a role in the differential response between groups. It is also possible that this difference was due to underlying variability in cell product batches. Although using the same donor minimizes inter-donor variability, individual infusion bags may still contain heterogeneous cell populations with differing immunomodulatory potency [47, 48]. Consequently, batch-to-batch differences could influence peripheral inflammatory responses and impact treatment efficacy. Future trials will incorporate additional equivalency and potency assays, as differences in therapeutic response may stem not only from dosing frequency but also from variability in the functional quality of stem cell preparations [49]. Establishing standardized potency measures will be critical to ensuring consistency and optimizing the nephroprotective potential of allo-hMSC therapy.

Both IL-6 and TNF-α play central roles in the inflammatory changes associated with aging [50]. Animal studies suggest that chronic inflammation initiates and accelerates age-related renal decline by disrupting metabolic pathways such as the pentose phosphate pathway, leading to impaired antioxidant defenses and increased oxidative stress, a hallmark of cellular senescence [17]. In the kidney, this may manifest as glomerular damage, podocyte loss, tubular atrophy, and interstitial fibrosis, ultimately contributing to eGFR decline [11, 51]. Longitudinal human studies further support this model, demonstrating that elevated levels of circulating IL-6 and TNF-α are independently associated with accelerated eGFR decline in older adults, even after adjusting for traditional risk factors [18–23].

Understanding how interventions like MSC therapy modulate inflammatory mediators may provide critical insight into their potential anti-aging effects on kidney function. Further correlation between kidney function and inflammatory markers is needed to clarify the mechanisms involved. Additionally, linking these outcomes to the activity of individual cell batches may help identify key features of potency and support the development of more precise assays to evaluate the therapeutic strength of MSC products in kidney-related inflammation. This mechanistic insight could not only be extended to other kidney diseases but may also support the broader use of MSCs as a strategy to counteract natural kidney senescence. Although we did not report inflammatory biomarker data or cell potency results in this manuscript, future work should focus on analyzing available longitudinal patient samples and correlating them with cell-based potency and equivalency assays to better understand and optimize the therapeutic potential of MSCs in the kidney.

This study has several limitations. First and foremost, the findings are based on secondary safety outcomes from a larger clinical trial in aging PD patients, and the study did not include non-PD participants. As a result, the generalizability of the findings may be limited, given the theoretical, though unproven, risk of worsened renal function in this population compared to non-PD aging individuals. In addition, the absence of younger healthy controls limits the ability to assess age-specific effects and establish baseline trajectories of renal aging. Taken together, these limitations prevent the current study design from isolating MSC-specific effects from those related to PD progression or normal aging. Future studies should include both age-matched healthy controls and younger participants to validate and contextualize the renal findings observed here. Additionally, because kidney function was not the primary endpoint of this trial, there may be baseline heterogeneity due to the lack of pretrial stratification focused on renal outcomes. However, the results provide proof-of-concept data that could support future clinical trials targeting anti-aging kidney therapies in asymptomatic individuals without CKD. The second limitation is the relatively small sample size, which may limit the generalizability of the findings. To address this, we used Bayesian analysis, which provides posterior probabilities that support clearer and more informed interpretations. This method is endorsed by regulatory agencies such as the FDA for early-phase trials due to its flexibility and suitability for small sample sizes [52]. Third, the exclusion of patients with significant renal comorbidities limits the applicability of these findings to individuals with more advanced CKD. Fourth, the study did not specifically track exposure to nephrotoxic medications or environmental toxins, which could have influenced kidney function and confounded the results. Finally, all participants were of similar age, preventing comparisons between older and younger individuals to evaluate age-specific effects.

## Conclusion

These results suggest that repeated intravenous infusions of 10 × 10⁶ allo-hMSCs/kg may improve eGFR and SCr levels in aging patients with PD without preexisting CKD. This represents an important first step in demonstrating the safety and proof-of-concept efficacy of allo-hMSCs as a potential therapy to stabilize and possibly improve kidney function in an aging PD population. Future studies should include well-designed efficacy trials involving diverse populations across different age groups and comorbidity profiles, with appropriate covariate control. In addition, functional potency assays and mechanistic studies are needed to better characterize the allo-hMSC secretome and understand the biological pathways driving the observed renal benefits.

## Supplementary Information

Additional file 1.

Additional file 2.
