# Supplementary material for: Allogeneic bone marrow-derived mesenchymal stem cells in the aging kidney: secondary results of a Parkinson’s disease clinical trial
Source: Stem Cell Res Ther. 2025 Sep 24;16:493. doi: 10.1186/s13287-025-04577-y (PMC12461955; doi:10.1186/s13287-025-04577-y)
Supplement: Supplementary file 1 — Additional file 1. [file 13287_2025_4577_MOESM1_ESM.pdf]

A randomized, double-blind, placebo-controlled trial of allogeneic bone marrow-derived Mesenchymal Stem Cells as a disease-modifying therapy for idiopathic Parkinson's disease

Department of Neurology, ***UTMOVE***
McGovern Medical School
University of Texas Health Science center at Houston

IND: 16756
HSC-MS-20-0150
NCT: 04506073

Version: 10.0

Date: 02/21/2024

Confidentiality Statement

The information in the document contains confidential and proprietary information that may not be disclosed to anyone other than the recipient study staff or members of the independent ethics committee/institutional review board (IRB) without the written permission of Dr. Mya Schiess unless disclosure is required by federal or state law or regulations. In any event, persons to whom the information is disclosed must be informed that the information is confidential and may not be disclosed by them. These restrictions on disclosure will apply equally to all future information supplied, which is indicated as confidential.

Principal Investigator:
Mya Schiess, MD: Professor, Adriana Blood Distinguished Chair in Neurology, Department of Neurology, Division Chief and Director of Movement Disorders & Neurodegenerative Diseases Program, University of Texas Health Science Center at Houston, McGovern Medical School. As a principal investigator on this study, she will be in charge of study design, outcome selection, regulatory submissions, patient recruitment, and pre-screening, clinical assessment, patient infusion monitoring, safety lab revisions, adverse event notifications, data interpretation, study regulatory reports and results dissemination.

Co-Investigators:
Jessika Suescun, MD: Senior Research Scientist in the Department of Neurology at UTHealth McGovern Medical School. As Co-Investigator, she will be responsible for managing the trial and contribute to study design, regulatory submissions, patient pre-screening, data collection, data cleaning, clinical data analysis and interpretation, and manuscript writing.

Timothy Ellmore, PhD: Professor at the City College of New York. As Co-Investigator, he will contribute to study design, and be in charge of exploring the MSC mechanism of action by planning, performing, processing, and evaluating neuroimaging data (Perfusion MRI, Perfusion MRI, and Neuromelanin MRI). Additionally, he would contribute to manuscript writing.

Collaborators:
Marie-Francoise Doursout, PhD: Professor in the Department of Anesthesiology at UTHealth McGovern Medical School. She will be responsible for planning, performing, processing, and evaluating changes in cytokine, chemokine, growth factors, angiogenesis, neurotransmitters, dopamine metabolites, and oxidative stress profiles.

Mohammad Shahnawaz, PhD, Assistant Professor of Neurology. As a Collaborators, Dr. Shahnawaz will be in charge of every single aspect related to alpha-synuclein oligomer assessment as target engagement. These include sample selection, sample analysis, data interpretation, and manuscript writing.

Juan Diego Martinez Lemus, M.D. As a Research Associate, he will be responsible for regulatory submissions, data cleaning, clinical data analysis and interpretation, and manuscript writing.

Research coordinator:

Rula Abuamouneh, MA (Research Coordinator II) As clinical coordinators, they are on staff in the Department of Neurology at UTHealth McGovern Medical School. They will provide day-to-day project coordination, assist with the preparation of budgets, supply purchases, pre-screening efforts, recruitment, patient scheduling, data collection, data storage, data quality control, IRB reports, and patient phone interview follow-ups.

Statistician:

Charles Green, PhD: Associate Professor in the Department of Pediatrics at UTHealth McGovern Medical School. As the study statistician, he will be in charge of trial design, outcome measures/endpoints selection, sample size calculation, effect size determination, block randomization, statistical analysis, and interpretation.

Consultants:

Jerome G. Saltarrelli, PhD: Assistant professor of the Immunology & Organ Transplantation Division, in the Department of Surgery at UTHealth McGovern Medical. As a consultant, Dr. Saltarrelli will advise the team on the analysis and interpretation of the donor-specific antibody reaction.

Other key personal:

Chris Adams, movement disorder fellow at the University of Washington. He will be responsible for analyzing some of the Peripheral and CNS Molecular Markers.

TABLE OF CONTENTS

ABBREVIATIONS 1
1. Study synopsis 4
2. Background: 6
2.1 Parkinson's disease (PD): 6
2.2 Mesenchymal stem cells 7
3. Rationale for using MSCs to treat PD 8
3.1 Proposed mechanisms of action: 9
3.2 Advantages 10
3.3 MSC Pharmacology 10
3.4 Pre-clinical and clinical experience of MSC in PD and other disorders 11
3.5 Safety and Tolerability 14
4. Design 16
5. Study objective and outcomes 17
5.1 Outcomes 17
5.1.1 Primary outcome 17
5.1.2 Secondary outcomes 17
6. Study subjects 19
6.1 Inclusion Criteria 20
6.2 Exclusion criteria 20
6.3 Selection and enrollment of Subjects 22
6.3.1 Advertisement 22
6.3.2 Pre-screening: 22
6.4 Estimated number of Subjects 22
6.5 Method of Assigning Subjects to Study Drug 23
7. Study intervention: Mesenchymal stem cell therapy 23
7.1 Allogeneic mesenchymal stem cells 23
7.2 Placebo 23
7.3 Delivery route and infusion rate 24
7.4 Facility: Clinical Research Unit 24
8. Study assessments 24
8.1 Demographics and complete medical history 24
8.2 Vital signs, orthostatic changes, weight and height 24

8.3 Physical and neurological examination ____ 25
8.4 Laboratory Test ____ 25
8.5 Rating scales ____ 25
8.5.1 MDS-UPDRS (Movement Disorder Society Unified Parkinson's disease Rating Scale) ____ 25
8.5.2 Modified H&Y (Hoehn and Yahr scale) ____ 26
8.5.3 TUG (Timed Up and Go Test) ____ 26
8.5.4 ADL (Modified Schwab and England Activities of daily living score) ____ 26
8.5.5 PDQ-39 (Parkinson's Disease Questionnaire) ____ 26
8.5.6 EuroQol- 5 Dimension (EQ-5D-5L) ____ 26
8.5.7 MoCA (Montreal Cognitive Assessment) ____ 27
8.5.8 UPSIT (University of Pennsylvania Smell Identification Test) ____ 27
8.5.9 C-SSRS (The Columbia Suicide Severity Rating Scale) ____ 27
8.5.10 Geriatric Depression Scale Short form (GDS-SF) ____ 27
8.5.11 Parkinson Anxiety Scale (PAS) ____ 27
8.5.12 RBD-Single-Question Screen (RBD1Q) ____ 28
8.6 Donor-specific antibodies (DSAs) ____ 28
8.7 Peripheral markers ____ 28
8.8 Lumbar Puncture ____ 29
8.9 Peripheral α-synuclein oligomers: ____ 29
8.10 Neuroimaging ____ 29
9. Potential Risks: ____ 31
10. Study schedule ____ 32
10.1 Clinical visits ____ 33
10.1.1 Screening visit ____ 33
10.1.2 Baseline visit ____ 34
10.1.3 Infusion visits ____ 35
10.1.4 Clinical and Safety Assessment ____ 37
10.2 Telephone or email evaluations ____ 38
10.3 Study Timeline ____ 39
10.4 Study Schedule ____ 40
11. Potential adverse events ____ 41
11.1 Infusion-Related Allergic Reactions ____ 41
11.1.2 Algorithm for response in case of an allergic reaction. ____ 42
11.2 Sensitization to Donor Specific Antibodies (DSA). ____ 43

11.2.1 Algorithm for the response to DSA 43
12. Management of adverse events 44
12.1 Adverse Events definitions 44
12.2 Serious Adverse Events (SAEs) 44
12.3 AE Relationship 45
12.4 AE/SAE Reporting 45
12.5 Follow-up AEs and SAEs 46
13. Study Modification/Discontinuation 46
13.1 Subject Withdrawal or Termination 46
13.2 Lost to Follow-up 47
13.3 Screen Failures 47
14. Statistical Considerations 47
14.1 Blinding and Randomization 48
14.2 Sample Size: 48
14.3 Statistical analysis 48
14.4 Interim analyses 49
15. Data collection and management 50
15.1 Records to Be Kept 50
15.2 Data Management 50
16. Data safety monitoring board (DSMB) 51
17. Intellectual Property and data sharing: 52
18. Ethics 52
19. Consent 53
20. Contacts 53
21. References 54
Appendix A. UK PARKINSON’S DISEASE BRAIN BANK CRITERIA 63
Appendix B. Modified Hoehn and Yahr 64
Appendix C. MoCA 65
Appendix D. MSC label sample 66
Appendix E. Medication Form 67
Appendix F. Modified Schwab and England Activities of Daily Living Scale (ADL) 68
Appendix G. Parkinson's disease Questionnaire (PDQ-39) 69
Appendix H. EQ-5D-5l 72
Appendix I. C-SSRS, Lifetime version 74

Appendix J. C-SSRS, Last visit version ____ 81
Appendix K. Geriatric Depression Scale (Short Form) ____ 88
Appendix L. The Parkinson Anxiety Scale (PAS) ____ 89
Appendix M. RBD-Single-Question Screen ____ 93
Appendix N. Red Flags ____ 94
Appendix O. Telephone Evaluation ____ 95

ABBREVIATIONS

α-Syn	Alpha- synuclein
ADL	Schwab and England activities of daily living scale
AE	Adverse event
ALS	Amyotrophic lateral sclerosis
ALT	Alanine aminotransferase
AS	Ankylosing spondylitis
AST	Aspartate transaminase
BBB	Blood brain barrier
BDNF	Brain-derived neurotrophic factor
BM	Bone marrow
BMI	Body mass index
BMMC	Bone marrow mononuclear cells
BP	Blood pressure
BUN	Blood urea nitrogen
CBC	Complete blood count
CCL	Chemokine C-C motif ligand
CD	Cluster of differentiation
CES-D	Center for Epidemiologic Studies Depression Scale
CLI	Critical limb ischemia
CNTF	Ciliary neurotrophic factor
CNS	Central nervous system
COPD	Chronic obstructive pulmonary disease
CRU	Clinical Research Unit
CSF	Cerebrospinal fluid
C-SSRS	Columbia Suicide Severity Rating Scale
CT	Computed tomography
CTCAE	Common terminology criteria for adverse events
DA	Dopaminergic
dL	Deciliter
DSMB	Data and Safety Monitoring Board
FDA	Food and Drug Administration
FGF	Fibroblast growth factor
FLAIR	Fluid attenuation inversion recovery
GCP	Good clinical practice
GDNF	Glial cell-derived neurotrophic factor
GFP	Green fluorescent protein
GFR	Glomerular filtration rate
GM- CSF	Granulocyte-macrophage colony-stimulating factor
GVHD	Graft-versus-host disease
Hgb	Hemoglobin

HGF	Hepatocyte growth factor
HIPAA	Health Insurance Portability and Accountability Act
HLA	Human leukocyte antigen
HR	Heart rate
Hr	Hour
H&Y	Hoehn and Yahr
ICM	Ischemic cardiomyopathy
IDO	Indoleamine 2,3-dioxygenase
IFN	Interferon
Ig	Immunoglobulin
IGF	Insulin-like growth factor
IL	Interleukin
IM	Intramuscular
IND	Investigational new drug
iNOS	Inducible nitric oxide synthase
INR	International normalized ratio
IRB	Institutional review board
IV	Intravenous
kg	Kilogram
LFT	Liver function test
MAO	Monoamine Oxidase
MDS	Movement Disorder Society
MHC	Major histocompatibility complex
MI	Myocardial infarction
MLR	Mixed Lymphocyte Response
mL	Milliliter
MoCA	Montreal cognitive assessment
MPTP	1-methyl-4-phenyl-1,2,3,6-tetrahydropyridine
MRI	Magnetic resonance imaging
MS	Multiple sclerosis
MSA	Multiple system atrophy
MSCs	Mesenchymal stem cells
NGF	Nerve grow factor
NINDS	National Institute of Neurological Disorders and Stroke
NSAIDs	Nonsteroidal anti-inflammatory drugs
NT	Neurotrophin
OHDA	Hydroxydopamine
OHRP	Office for Human Research Protections
OTC	Over-the-counter
PBMC	Peripheral blood mononuclear cell
PD	Parkinson's disease
PDGF	Platelet-derived growth factor
PD-NMS	Parkinson's disease non-motor symptom questionnaire
PDQ-39	Parkinson's disease questionnaire – 39 item version

PI	Principal investigator
PRI	Panel reactive antibody
PT	Prothrombin time
PTT	Partial thromboplastin time
QoL	Quality of life
RBD	Red blood cells
SAE	Serious adverse event
SC	Subcutaneous
SD	Standard deviation
SDF	Stromal cell-derived factor
SLE	Systemic lupus erythematosus
SN	Substantia nigra
SNRIs	Serotonin-norepinephrine reuptake inhibitors
SSRIs	Selective serotonin reuptake inhibitors
TCAs	Tricyclic antidepressants
TE	Echo time
TGF	Transforming growth factor
Th	T helper cell
TMC	Texas Medical Center
TNF	Tumor necrosis factor
TR	Repetition time
TUG	Timed up and go test
UC	Umbilical cord
UT	University of Texas
UPDRS	Unified Parkinson's disease Rating Scale
UPSIT	University of Pennsylvania Smell Identification Test
VEGF	Vascular endothelial growth factor
VS	Vital signs
WBC	White blood cells
Wk	Week

1. Study synopsis

**FULL STUDY TITLE**	Allogeneic Bone marrow-derived Mesenchymal Stem Cells as a disease-modifying therapy for idiopathic Parkinson's disease: Phase IIa double-blind randomized controlled trial.
**CLINICAL PHASE**	Phase IIa
**INVESTIGATORS/ STUDY GROUP**	Principal Investigator: Mya Schiess, MD<br>Co-Investigators: Jessika Suescun, MD; Timothy Ellmore, PhD<br>Collaborator: Marie-Francoise Doursout, PhD<br>Claudio Soto, PhD, Shivika Chandra, MD<br>Mohammad Shahnawaz, PhD<br>Juan Diego Martinez Lemus, MD<br>Coordinators: Rula Abuamouneh, MA<br>Statistician: Charles Green, PhD<br>Consultant: Sean Savitz, MD; Jerome G. Saltarrelli, PhD<br>Other non-key personnel: Christopher Adams, MD; Kelly Block, MD
**STUDY OBJECTIVE**	To select the safest and most effective number of repeat doses of allogeneic bone marrow-derived mesenchymal stem cell (MSC) infusions to slow the progression of Parkinson's disease (PD).
**STUDY RATIONALE**	Neuroinflammation plays a key role in the pathogenesis of PD; thus supporting the rationale for using an immunomodulatory therapy such as MSCs for restoring homeostasis to the neuronal-glial microenvironment. Preliminary findings from our Phase I trial showed that a single intravenous infusion of MSCs was safe and well-tolerated.
**STUDY SITES**	One site: The University of Texas Health Science Center at Houston (UTHealth)
**STUDY PERIOD**	4 years
**STUDY POPULATION**	The target population includes 45, men and women, between 50 and 79 years of age who meet UK Brain Bank clinical diagnostic criteria for PD. Subjects will be randomized 1:1:1 to 1 of the three treatment arms.
**STUDY DESIGN**	Phase IIa double-blind, randomized controlled trial of allogeneic bone marrow-derived MSCs as a disease-modifying therapy for PD. The study design includes 3 treatment arms: a) 2 infusions of 10 X 10^{6} MSC/kg and 1 placebo every 4 months; b) 3 infusions of 10 X 10^{6} MSC/kg every 4 months; c) 3 infusions of placebo every 4 months. All subjects receive 3 infusions, at 4 month intervals, and followed for 88 weeks.

**MAIN INCLUSION/ EXCLUSION CRITERIA**	Inclusion Criteria<br>Men and women between the ages of 50 and 79; DOD 4 to 10 years; UK Brain Bank PD diagnosis, mild microsmia or anosmia, and robust response to dopaminergic therapy.<br>Exclusion criteria<br>Atypical, vascular, or drug-induced Parkinsonism. A MoCA score of less than 25.
**DOSAGE:**	Intravenous infusion of either placebo or 10 $\times 10^6$ MSC /kg of body weight.
**DOSAGE: JUSTIFICATION**	Available literature reported safety data and efficacy on dosages ranging from 1–150 $\times 10^6$ MSC/kg for other pathologies. Preliminary findings from our Phase I trial showed 10 X 10^6 MSC/kg has the largest effect on reducing motor symptoms and improving quality of life.
**DURATION OF TREATMENT**	Each patient will receive 3 infusions of either placebo or MSC at a rate of 2 ml/min, followed by a 4-hour monitoring period.
**PRIMARY OUTCOME MEASURE(S)**	To select the safest and most effective number of repeat doses of allogeneic bone marrow-derived MSC with the aim to slow the progression of PD as determined by the change in the motor section of the MDS-UPDRS score (≥ -5) in each active treatment arm versus placebo between baseline and 62 weeks follow-up.
**SECONDARY OUTCOME MEASURE(S)**	Aim 2. Assess safety, tolerability, and secondary measurements of clinical efficacy including; cognition, behavioral changes, motor function, disability, and QOL.<br><br>Aim 3. Explore putative mechanisms of action for the effect of MSCs.
**SAMPLE SIZE CONSIDERATIONS**	Current estimates assume N = 45 participants randomized in 1:1:1 fashion to three conditions and medium effect size (MDS-UPDRS III difference = 5.2).

2. Background:

2.1 Parkinson's disease (PD):

Neurological disorders are the leading cause of disability worldwide, and Parkinson's disease (PD) is the fastest-growing. It is predicted that by 2040 PD will become a pandemic affecting 17.5 million people worldwide[1], due in large part to the aging of our population, increased longevity, decreased smoking, and industrialization. Despite the enormous progress made in understanding the pathophysiology and the clinical expression of this chronic slowly progressive disease, there is no biological marker for diagnosis. Idiopathic PD remains a diagnosis made clinically and most often in movement disorders centers by using the UK Brain Bank criteria for PD. Neuropathologically, PD is defined by dopaminergic loss in the substantia nigra pars compacta (SNc) and the presence of misfolded α-synuclein aggregates called Lewy bodies and Lewy neurites. Braak *et al*[2] described how the neuropathology progresses in a repeatable pattern and distribution with worsening severity over six stages of the disease; non-motor and motor symptoms localize neuroanatomically to the structures affected by the disease process. The premotor or non-motor symptoms include olfactory loss, constipation, anxiety, depression, autonomic dysfunction, and sleep disorders such as REM sleep behavior disorder (RBD). These non-motor features may present for up to a decade; importantly, by the time patients manifest motor symptoms, roughly 70% of the nigral dopaminergic neurons have been lost. There are a multitude of pharmacological formulations and some surgical therapies effective in alleviating motor symptoms. Although both medical and surgical therapies can 'normalize" movements especially early in the disease process, there is no spontaneous remission of motor symptoms within the natural history of idiopathic Parkinson's disease. Additionally, there are no FDA approved therapies to arrest or delay continued degeneration of neurons[3,4].

There is considerable evidence supporting the critical role of neuroinflammation in the degenerative process. Several mechanisms including alpha-synuclein protein aggregation (due to protein misfolding or degradation), oxidative stress (reactive oxygen and nitrogen species), mitochondrial dysfunction, endoplasmic reticulum stress and dysfunction of neurotrophic factors[5] are thought to trigger the inflammatory process. The inflammatory response is orchestrated by interactions of glial cells, peripheral lymphocyte infiltration, a disrupted blood brain barrier (BBB)[6-8], and signaling molecules that elicit a coordinated reaction between the CNS and the peripheral immune system. The purpose of this response is to protect and repair the tissues involved and restore homeostasis. The neuroinflammatory condition is well described in human post-mortem, *in vivo* (MPTP, 6-OHDA, rotenone, LPS, and SNc extracts) and *in vitro* models. Studies have shown activated glia, high levels of expression of major histocompatibility complex type II (MHC-II), integrins, neurotrophins, peripheral B and T-lymphocytes infiltration, related inflammatory molecules, pro-inflammatory cytokines and chemokines and changes in growth factors[9-18]. Inflammation also plays a significant role in the pathogenesis of both toxin-induced

and genetic models for PD[19,20], and epidemiological studies on the risk-lowering effects of anti-inflammatory drug regimens support its key role[21-23]. Our group has studied extensively the role of the peripheral immune system in PD neurodegeneration by using an LPS rat model[24,25], human-derived cultured glial cells[26,27], patients CSF[28,29], and plasma[30]. Ultimately, a chronic inflammatory state is perpetuated which leads to mitochondrial dysfunction, derangement in the ubiquitin-proteasomal degradation pathway, cellular swelling, excitotoxicity, release of free radicals and nitric oxide, decreased secretion of trophic factors that compromises cellular function and neuronal survival.

Together, these results indicate that a unique adaptive immune response is indeed occurring in patients with iPD and changes as the disease progresses. These aggregate data support the necessity of an immune modulator molecule to arrest this process, such as mesenchymal stem cells (MSCs).

2.2 Mesenchymal stem cells

Mesenchymal stem cells (MSCs) were first isolated in 1966 by Friedenstein *and colleagues*[31]. MSCs are multipotent cells that have the potential to differentiate into various cells of a mesodermal lineage. Bone marrow aspirate has been the primary source for MSC; however, they can also be isolated from neonatal tissue, adipose tissue, peripheral blood, dental pulp, amniotic fluid, placenta, and umbilical cord blood.

MSCs possess several advantages over other stem cell therapies that make them an attractive therapeutic for neurodegenerative disorders[32-34], in that they are easily procured and expanded on a large scale, and have little to no ethical issues or controversies surrounding them when derived from the bone marrow of healthy volunteers. MSCs can differentiate along several lineage pathways, are capable of migrating to sites of injury, including across a disrupted blood brain barrier. They possess immunomodulatory and anti-inflammatory properties, a low probability of being tumorigenic, and are amenable to genetic modification. The lack of a sustainable effect is a potential weakness of MSCs therapy. Repeated dosing may be required, and MSC infusions may fall short of modulating the individual’s intrinsic anti-inflammatory response.

Multiple companies worldwide manufacture mesenchymal stem cells (MSC) and offer them to the public, including Cynata Therapeutics, RoosterBio, Pluristem Therapeutics, BioEden, Regenexx, and many more. In fact, MSCs are now the most common cell type used in regenerative medicine. The 2018 report by BioInformant disclosed that in 2017, the global Mesenchymal Stem Cells market size was $170 million, and it is expected to reach $250 million by the end of 2025.

To-date only four MSC based therapies have been approved overseas, and neither have been for a neurodegenerative disorder. In 2012, Health Canada issued marketing approval for Prochymal (Osiris) to treat children with acute GvHD. Temcell HS allogeneic MSC therapy from JCR Pharmaceuticals was approved in Japan in 2015 for acute GvHD in bone marrow transplant

patients, TEMCELL generated ¥ 0.7 billion sales for JCR in 2016 fiscal year. Stempeucel (Ex vivo cultured adult allogeneic MSCs) was approved in India in 2016 for Critical limb ischemia due to Buerger's disease. Additionally, in 2018 Alofisel (Allogeneic expanded adipose-derived MSCs) from TiGenix, received approval from the European Commission to treat Crohn's-related enterocutaneous fistular disease.

3. Rationale for using MSCs to treat PD

Neuroinflammation plays a critical role in the development and progression of PD. Several experimental approaches to protect, rescue, and even restore dopaminergic neurons are under investigation[35-37]. Various types of stem cells have shown promising results as effective neuro-restorative treatments for a range of neurological disorders[38,39]. Decades worth of PD studies have focused on direct intra-striatal transplantation of fetal tissue with mixed results[40-42]. The ethical considerations regarding the source of the neural tissue have discouraged further investigation into the feasibility of this therapy. However, a growing body of literature indicates that MSC possess regenerative and immunomodulatory properties that can be harnessed for therapeutic applications. MSC have been studied extensively in multiple animal models of PD for over 10 years[43-51].

The highly plastic immune-regulatory function of MSC allows for modulation of the immune milieu by interacting with the neural-glial microenvironment, producing and responding to cytokines, chemotaxis, growth factors, and cell activation[32,52,53]. Several studies using animal models of PD have found that intravenous (IV) or intra-arterial administration of MSCs decreased DA neuron loss, decreased inflammatory cytokine production or microglial activation, or reduced α-synuclein oligomerization. Indeed, the degeneration of dopaminergic (DA) neurons in the substantia nigra pars compacta in iPD may be reversible, and previous studies have shown that MSCs can protect and stimulate regeneration in damaged recipient DA cells[54-57]**.** The potential therapeutic benefit of MSCs therapy is hypothesized to rely primarily on paracrine actions, exosome activity, and direct cell-to-cell modulation of host immune cells[58]. The paracrine actions include the release of trophic and immunosuppressant factors and can enhance angiogenesis and neurogenesis, inhibit fibrosis and apoptosis, and suppress free radical formation. The trophic factors stimulate the recruitment, retention, proliferation, and differentiation of tissue-residing stem cells, and produce an extracellular matrix that supports neural cell attachment, growth, and axonal extension[38]. In turn, activated microglia produce trophic factors that stimulate DA neural regeneration and provide signals that promote endogenous neural stem cells and glial differentiation. MSCs secrete extracellular vesicles known as exosomes. Exosomes elicit diverse cellular responses as an intercellular communication vehicle and support the maintenance of a dynamic and immune homeostatic within the tissue microenvironment. They potentially restore tissue function by providing catalytically active enzymes to promote homeostasis[58]. All these actions can create a favorable environment for regeneration and allow a restorative process to occur[59].

Our collaborators at the University of Texas Health Science Center have demonstrated the safety and tolerability of intravenously administered MSCs in a pilot study of patients with recent ischemic stroke[60], and we demonstrated an overall effect of MSC infusion on limb function in PD animal models[50]. In 2015, our research group obtained an IND to study the safety and tolerability of intravenous administration of bone marrow-derived MSCs in patients with mild to moderate PD. This Phase I study demonstrated that a single IV infusion of MSCs is safe, well-tolerated, and not immunogenic at doses that range from 1 to 10 X 10^6 IV MSCs/kg in subjects with mild to moderate PD. The most significant change in peripheral inflammatory markers steadily declined after 3 months with some residual effect at 6 months; based on this data, we would like to test 2 infusions vs. 3, at 4 month intervals for primary effect at 62 weeks and total follow-up at 88 weeks.

3.1 Proposed mechanisms of action:

DA neurons are damaged early in the PD disease process, which in turn causes activation of microglia. As the neurons degrade, α-synuclein is released and taken up by the microglia, which in turn recruits circulating immune cells that infiltrate into the CNS, secrete proinflammatory cytokines, chemokines, and reactive oxygen species. These events eventually lead to a chronic inflammatory state and accelerated neuronal death through oxidative stress and apoptosis. Based on evidence from previous studies, we hypothesize that IV injection of MSC will help to reduce the neuroinflammatory response through the manipulation of microglia-mediated neuroinflammation.

The potential benefit of MSC therapy is hypothesized to rely on three specific actions. Paracrine immunomodulation and exosome-mediated activity play significant roles.

- Paracrine actions:

  Paracrine factors as cell-derived microvesicles that perform intercellular communication. The release of trophic factors and immunosuppressant factors can enhance angiogenesis, inhibit fibrosis and apoptosis, and suppress free radical formation. The trophic factors stimulate the recruitment, retention, proliferation, and differentiation of tissue-residing stem cells, and produce an extracellular matrix that supports neural cell attachment, growth, and axonal extension. Together all these actions can create a favorable environment for regeneration and allow for an intrinsic restorative process to occur[59,61-65].

  MSCs can secrete numerous growth factors and cytokines, including the following: Brain-derived neurotrophic factor (BDNF), nerve growth factor (NGF), glial cell-derived neurotrophic factor (GDNF), fibroblast growth factors 2 and 8 (FGF2 and FGF8), ciliary neurotrophic factor (CNTF), neurotrophin-3 (NT-3), hepatocyte growth factor (HGF), platelet-derived growth factor (PDGF), vascular endothelial growth factor (VEGF), insulin-like growth factor 1 (IGF-

1) and stromal cell-derived factor-1 (SDF-1) [66-71]. GDNF, BDNF, FGF, and CNTF have been shown to elicit neurotrophic and neuroprotective effects on DA neurons[72-75]. These neuro-regulatory molecules promote neuronal survival and regeneration, as well as endothelial cell proliferation and angiogenesis[67,70,76].

MSCs also activate microglia and astrocytes[77]. Activated microglia produce trophic factors that stimulate DA neural regeneration and provide signals that promote endogenous neural stem cells and glial differentiation[78-82]. MSCs have been shown to suppress or activate immune responses; through cell-cell contact and release of different factors, they evoke a cellular immune response by expressing immunomodulatory mediators such as indoleamine 2,3-dioxygenase (IDO) and induced nitric oxide synthase (iNOS). On the other hand, MSC secretion of the anti-inflammatory cytokines TGFβ, IL-1ra, IL-10, and subsequent down-regulation of IL-2 and IL-15 may suppress the activities of macrophages, neutrophils, T and B lymphocytes, dendritic cells and natural killer cells[83-89].

- Exosomes:

  MSC secrete extracellular vesicles known as exosomes derived from the endosomal membrane. Exosomes carry nucleic acids, lipids, and proteins, including micro RNAs; they elicit diverse cellular responses and can function as an intercellular communication vehicle for modulation of cellular processes. Exosomes support the maintenance of immune homeostasis within the tissue and allow for a dynamic response to the external tissue microenvironment. They potentially restore tissue function by providing catalytically active enzymes to promote homeostasis.[58,90]

3.2 Advantages

MSC possess several advantages over other stem cell therapies that make them an attractive therapeutic for neurodegenerative disorders[32-34]:

a) Easily procured and expanded on a large scale.
b) Little to no ethical issues or controversies.
c) Able to differentiate along several lineage pathways.
d) Capable of migrating to sites of injury, including crossing the brain-blood barrier.
e) Immunomodulatory and anti-inflammatory properties.
f) Low probability of being tumorigenic.
g) Amenable to genetic modification.

3.3 MSC Pharmacology

<u>Absorption and Distribution</u>:

Animal studies have shown that following intravenous administration, the majority of allogeneic MSCs are trapped in the lungs upon the first passage and can remain there for up to approximately a week. Under inflammatory conditions, MSC may persist at distal sites of injury up to 5-8 days post-injection [91,92]. In rat models of PD, xenogeneic MSC injected I.V. were detected in brain tissue between 2-7 days post-administration [47,93].

Metabolism and Excretion:

Allogeneic MSC migrate to the spleen and the liver within 2-5 days post-injection and are presumably phagocytized by local macrophages at these sites within 14 days [92].

Toxicology:

Up to 2 X 10^6 cells/mouse have been injected (equivalent to 1 X 10^{11} cells/kg) with no serious adverse effects [94]. The development of an immune response directed against allogeneic MSC has been reported in several different animal models; thus far, the only measurable consequence appears to be decreased survival and engraftment of the injected cells [95-101].

3.4 Pre-clinical and clinical experience of MSC in PD and other disorders

There is substantial evidence from animal models and from chronic human diseases that MSC transplantation by intravenous administration can restore immune system homeostasis and stimulate DA regeneration, thus having the potential to slow the progression of PD.

Animal Models

Several studies using animal models of PD have found that intravenous (I.V.) or intra-arterial administration of MSC can protect dopaminergic neurons and may improve motor function[45-47,4960]. Table 1 provides a list of animal studies that assess the effect of intravenous MSC, which includes: 6-OHDA, Preformed fibril model + MPTP, MPTP + 3-NP, MPTP, and a transgenic MSA model. Most of the studies showed dopaminergic survival (increased TH+ neurons), reduced inflammatory cytokines, decreased microglial activation, and some behavioral improvement. Intriguingly, some of these models suggest that the therapeutic effects of intravenous delivery of MSCs may not even be dependent on the status of the BBB because of the profound impact of MSCs on the peripheral immune responses. Our group's recent meta-analysis of 25 studies of MSC therapy in animal models showed safety and efficacy. The meta-analysis demonstrated that the I.V. route of administration had a more substantial effect on limb function as well as improved rotational behavior.[50]

Clinical Experience in Neurodegenerative disease

Table 1. Animal Models						
**Model**	**Cell type**	**# cells/kg**	**Injection site**	**Follow-up**	**Results**	**Ref**
6-OHDA rat	MSC transfected with CDNF	2 x 10^5	Intrastriatal, intraventricular or jugular vein	6 weeks	Behavioral improvement (better for intrastriatal) and Increased TH+ neurons	102
6-OHDA rat	Human adipose derived MSC	?	Intraventricular into SN	3 days	Evidence of S100 positive BDNF cells and endothelial cells derived from MSC	49
6-OHDA rat	Human BM-MSC (up to 6th passage)	1 x 10^7	Femoral vein	26 or 80 days	Increased TH+ neurons Behavior improved out to 80 days. No evidence of hMSC engraftment	47
6-OHDA rat	Rat BM-MSC GFP transfected (2nd passage)	1 x 10^7	Femoral vein	2d. 1wk. 4 wks	Cells in lung and brain at 2 days, also at 1 week	48
MPTP mouse and a-syn-inoculation	Human MSCs (2nd passage)	1 x 10^6	Tail Vein	7 days	Reduced levels of immunoreactivity for both a-synuclein and ThT	103
(PLP)- αSYN mouse (MSA)	GFP positive syngeneic MSC	5 x 10^5	Tail Vein	4 weeks	No behavior improvement Reduced inflammatory cytokines in the brain	104
MPTP and 3-NP (MSA)	Human BM-MSC (6th passage)	1 x 10^6	Tail Vein	4 weeks	Increased TH+ Nuer-N+ cell survival Decreased microglial activation	45
MPTP mouse	Human BM-derived MSC (6th passage)	1 x 10^6	Tail Vein	2-4 weeks	Reduced α-syn in the brain, increased autophagolysosomes in DA neurons	46
MPTP rat	BMMC or BM-MSC (4th passage)	5 x 10^6 1 x 10^6	Jugular vein	1-8 days	No behavioral improvement MSC injected had slightly better neuron survival	43
MPTP mouse	Mouse BM-derived MSC	1 x 10^5	Tail Vein	3, 7, 14, and 28 days	Recovery of BBB integrity, suppression of microglial activation and Increased TH+ neurons survival	105

Venkataramana *et al.* in an open-label Phase I study demonstrated the safety of autologous MSC transplantation into sublateral ventricular zone of 7 PD patients, reporting a decrease by 18% of the conventional "off" Unified Parkinson's disease Rating Scale (UPDRS) -Total score after one year and 26% following 2 years post-treatment. There was a substantial reduction in total levodopa dosing for the PD patients with no adverse effects reported[106]. The same group reported results from a 2 year pilot clinical study demonstrating feasibility, safety, and efficacy of bilateral transplantation of allogeneic MSCs in 8 PD patients with a mean improvement of 31% in the off UPDRS-Total score. Neither of these studies reported any serious adverse events related to treatment [107].

To date, there have been studies of the effects of MSC therapy on three neurodegenerative diseases, including multiple system atrophy (MSA), multiple sclerosis (MS) and amyotrophic lateral sclerosis (ALS):

- Dongmei *et al.* showed that intrathecal injection of umbilical cord mesenchymal stem cells (UC-MSC) is safe and can delay the progression of neurologic deficits in MSA-C patients. They reported 3 symptoms as side effects after intrathecal injection, including dizziness, back pain, and headache that disappeared between 1-3 days, and were attributed to the lumbar puncture procedure[108]. Xi *et al.* tried a comprehensive cell-based neurorestorative therapy (I.V. UC-MSC, intrathecal Schwann cells, and transplantation of olfactory ensheathing cells) for patients with multiple system atrophy, in which they proved safety and to some extent benefit with improvement in total Unified Multiple System Atrophy Rating Scale (UMSARS)[109]. Lee *et al.* evaluated the feasibility of consecutive intra-arterial and intravenous MSC treatment for MSA patients and proved safety and a significant improvement on the UMSARS[110].
- Karusis *et al.* showed that one intrathecal and one intravenous administration of MSCs in patients with MS and with ALS is clinically feasible, relatively safe, and induces immediate immunomodulatory effects[111]. Connick *et al.* performed an open-label Phase 2a proof-of-concept study and concluded that autologous MSCs were safely given in one I.V. dosage for secondary progressive MS. There was evidence of structural, functional, and physiological improvement after treatment.[112] Liang *et al.* used allogeneic MSC transplantations for the treatment of multiple sclerosis resulting in disease stabilization and improved quality of life in a single MS patient [113]. Hou *et al.* showed that I.V. and intrathecal administration of umbilical cord and bone marrow-derived MSCs over 4 years in a patient with relapsing-remitting multiple sclerosis was safe and effective[114].
- Mazzini *et al.* showed safety of MSC transplantation in the dorsal spinal cord in patients with amyotrophic lateral sclerosis[115]. Additionally, Navabi *et al.* have also shown that intravenous (IV) and intrathecal (IT) transplantation of BM-derived stromal cells is safe and feasible in patients with amyotrophic lateral sclerosis[116].

Clinical Experience in other diseases

Allogeneic MSC have been used worldwide in research for the past decade, available data indexed in PubMed contains results for a wide range of diseases including acute graft-versus-host disease (aGVHD), ankylosing spondylitis, systemic lupus erythematosus (SLE), Crohn's disease, human immunodeficiency virus (HIV), ulcerative colitis, psoriasis, leukemia, diabetes mellitus (DM), chronic obstructive pulmonary disease (COPD), interstitial lung disease (ILD), asthma, partial medial meniscectomy, orthogenesis imperfecta, spinal cord injuries, ischemic cardiomyopathy, myocardial infarction, heart failure, critical limb ischemia (CLI) and cerebral palsy.

Currently, there are 84 allogeneic mesenchymal stem cell trials registered in the U.S. National Institutes of Health (NIH) database conducted by investigators in the USA, Australia, Belgium, China, Denmark, Greece, Germany, India, Iran, Japan, Korea, Malaysia, Spain, and Vietnam.

Dosage, Intervals, and Route of Administration for allogeneic MSC

Different delivery routes for MSC have been tested, including intra-arterial, I.V., intra-articular, intrathecal, intramuscular, transendocardial, and intra-bone marrow. An I.V. infusion is the least invasive and therefore the safest. Available literature reported eleven studies performed in the USA, Australia, Japan and China that used I.V. infusion as a delivery route; none of them had a serious adverse event (SAE) related to I.V. infusion of MSC and none had specific reactions following serial infusions[113,114,117-126]. Dosages of I.V. infusion used in the studies mentioned above ranged from 1–150 $\times$ 10^6 MSC/ kg, with each of the doses proven to be safe and showing some degree of efficacy. Eight of the eleven trials included serial infusions ranging from 2 to a maximum of 12 dosages, scheduled as often as twice a week or as far apart as once every 6 months. Serial infusions are an important consideration in PD since our data show that PD is a chronic neuroinflammatory condition[127] that is relentlessly progressive and unlike an acute injury, requires serial infusions to modify and maintain the immune system response.

3.5 Safety and Tolerability

Monitoring for safety after administration of undifferentiated MSC focus on acute hypersensitivity reaction and a sub-acute or delayed antibody-mediated reaction that can occur within 3-4 weeks of the infusion. A primary concern, although extremely rare, in MSC infusion therapy is the development of a hypersensitivity reaction. Transient (lasting several hours) fever is the most common but again rare administration side effect. An acute, more serious hypersensitivity reaction causing fever, confusion, kidney, liver, or other organ damage, hemolytic anemia or pneumonitis has a very low risk of occurrence. Additionally, the systemic or intravenous route of administration means that the MSC can be distributed to many different tissues and organs in the body and we know that most will end up in pulmonary tissue and that the small molecules will distribute throughout the circulation.

The immune privileged status of MSC has been demonstrated using expanded undifferentiated and differentiated MSC *in vitro* culture. The cell surface of these cells expresses intermediate levels of human leukocyte antigen (HLA) major histocompatibility complex (MHC) class I molecules and negligible levels of HLA class II and Fas ligand. There is no expression of the co-stimulatory molecules B7-1, B7-2, CD40 or CD40L, and when exposed to allogeneic lymphocytes, they fail to elicit a reactive response[128]. However, with alloantibody production following repeated doses of MSC, no life-threatening host vs. graft responses have been reported[96,129,130].

Lalu *et al*[131] reviewed the safety of systemic MSC administration in humans in a meta-analysis of 36 studies. Based on these analyses, MSC therapy appears safe with transient fever as the only significant administration side effect.

To prove the safety and feasibility of the use of intravenously deliver allogeneic bone marrow-derived MSCs in patients with PD, our group designed and conducted the first USA single-center open-label dose-escalation phase 1 study and we demonstrated that a single infusion of allogeneic

MSCs is safe and well-tolerated in mild to moderate PD patients. The treatment effect likely relies on peripheral immune modulation. The study recruited 20 subjects with mild-moderate PD that were sequentially assigned to one of four doses: 1, 3, 6, or 10 X 10^6 MSCs/kg given IV and evaluated at 3, 12, 24, and 52 weeks post-infusion. Primary outcome safety measures were defined as the absence of immediate transfusion reaction, study-related adverse events, organ damage, or immunogenic reactions. Secondary outcomes were defined by the therapy's impact on peripheral markers, PD progression, and changes in brain perfusion.

All 20 patients received a single MSCs IV infusion. During the first 24 hrs post-infusion, three patients reported TEAEs, one with phlebitis, one with an antecubital fossa hematoma, and one with a headache (*Table 2*). The first patient had 3 cm superficial phlebitis Grade 2 Infusion-Related Allergic Reactions (NCI-CTCAE criteria) that required local medical management. The other two patients had mild symptoms and did not require any treatment. All patients left the research unit with full resolution of their symptoms. In subsequent follow-up, 50% of the patients reported TEAEs; most of them were mild, with dyskinesia (20%, N=4) and hypertension (20%, N=4) as the most common. Two of the patients experiencing dyskinesias required levodopa reduction for resolution and the remaining resolved without intervention. All hypertension cases were reported between 3 and 12 weeks after infusion and were followed by their primary care physicians. Of those, three cases were transient, and one patient was diagnosed with stage 2 hypertension and is currently under medical management.

There was one SAE during the study. One patient with a 4-year history of lymphocytosis was diagnosed with chronic lymphocytic leukemia (CLL) 8 months after the infusion. Patient 10B (stem cell dose 3 X 10^6 MSC/kg) was enrolled with a lymphocyte percentage of 50.7; our laboratory range is 20-40. The PI determined that the elevation was not clinically significant, taking into consideration that; a) the study team was not aware of a previous history of elevated lymphocytes; b) the patient had no symptoms or positive findings at the physical examination with the exception of the ones related to PD; c) the patient met all the inclusion criteria and d) the entirety of the labs including CBC and differential (WBC count and lymphocyte absolute number) were within normal range. A CBC was obtained at each visit except for the infusion visit; the patient's lymphocyte percentage remained out of range during the whole study with a normal WBC and lymphocyte absolute number (which is the current value used for CLL guidelines[132]). One hundred twenty-six days after the MSC infusion, the patient informed us that their PCP had been following them for 4 years for an elevated lymphocyte % and that at that point, they had a significant increase in lymphocyte absolute number. The patient was evaluated by a hematologist-oncologist who made the diagnosis of Chronic Lymphocytic Leukemia. Based on a low disease burden and the fact that the patient has remained asymptomatic, a therapeutic intervention was not recommended, and the patient remains untreated and under scheduled monitoring. Our DSMB was immediately informed and based on the absence of literature on this adverse event in studies with MSCs; they labeled the SAE as possibly related. The patient remained asymptomatic at the end of the study.

Laboratory assessment showed a transient decrease in lymphocytes in 30% (N=6) of the patients and a transient increase in basophils in 20% (N=4) of the patients. These changes did not last more than three months and were not related to any specific disease process. Suicidal ideation did not change during the study. There was no relationship between the incidence of adverse events and dose. No trends emerged from physical examinations or neurological examinations.

There was no response to donor HLA over the 12-month study. At 52 weeks CCL2 declined by 15% (p<0.01), and CCL22 by 29% (p<0.05), along with an increase in BDNF 46% (p<0.05). The highest dose had the most significant effect on reducing OFF UPDRS motor -14.4 (p<0.01) and total scores (-20.8, p<0.001). Further, there was an increase in basal ganglia perfusion at 24 weeks (p<0.001).

Table 2. Treatment-Emergent Adverse Events (TEAE)

Body System	*Adverse Reaction*	*Group A N=5 (100%)*	*Group B N=5 (100%)*	*Group C N=5 (100%)*	*Group D N=5 (100%)*	*AE Relationship to MSC*
Cardiovascular	Hypertension	2 (40%)	2 (40%)	0	0	Unlikely related
	Phlebitis[b]	0	0	0	1 (20%)	Related
	Hematoma[b]	0	0	1 (20%)	0	Related
Gastrointestinal	Nausea[a]	1 (20%)	2 (40%)	0	0	Possibly related
Neurologic	Headache[b]	0	1 (20%)	0	0	Possibly related
	Dyskinesia	1 (20%)	0	2 (40%)	1 (20%)	Probably related
Hematology	CLL	0	0	1 (20%)	0	Possibly related
Lab	↓Lymphocytes[a]	3 (60%)	2 (40%)	0	1 (20%)	Possibly related
	↑Basophils[a]	1 (20%)	1 (20%)	1 (20%)	1 (20%)	Possibly related

Data shows n and (%) per dose group. CLL, Chronic Lymphocytic Leukemia. [a]: Mild and transient. [b]There were three AEs related to the infusion procedure (phlebitis, antecubital fossa hematoma, and headache). The remaining AEs were reported at follow-up and 3 patients required management, one for hypertension and two for dyskinesias.

4. Design

Phase IIa double-blind, randomized controlled clinical trial of allogeneic bone marrow-derived mesenchymal stem cells as a disease-modifying therapy for PD. Forty-five men and women between 50 and 79 years of age who meet the UK Brain Bank criteria for idiopathic PD and who have a Hoehn and Yahr of <3 in the OFF medicine state and are between 3-10 years from the onset of disease diagnosis will be recruited to participate. Subjects will be randomized 1:1:1 to 1 of the

3 following groups: 2 infusions of 10 X 10^6 MSC/Kg every 4 months and one placebo infusion; 3 infusions of 10 X 10^6 MSC/Kg every 4 months and 3 infusions of placebo every 4 months. *Figure 1***.** The subjects will be followed for a total of 88 weeks.

Figure 1. Study Design

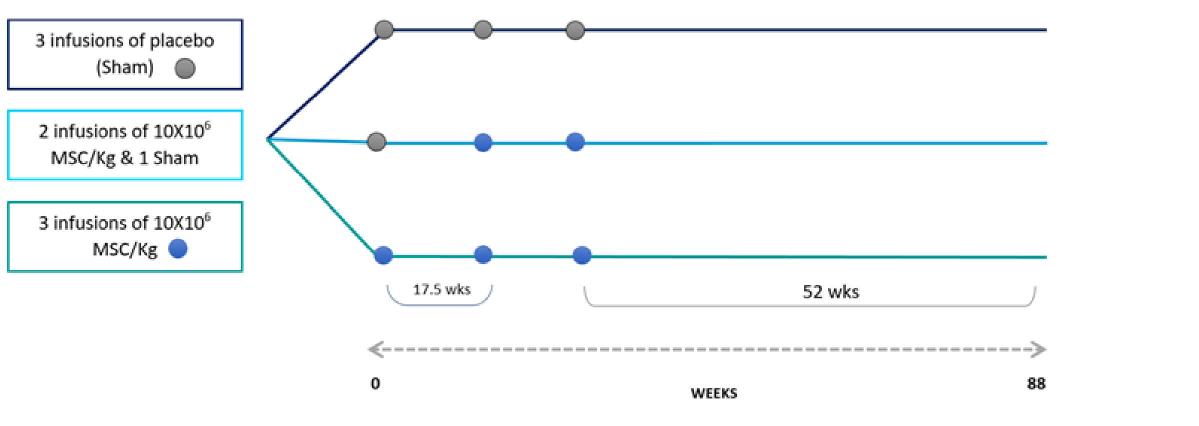

5. Study objective and outcomes

The primary objective of this study is to select the safest and most effective number of repeat doses of allogeneic bone marrow-derived mesenchymal stem cell infusions to slow the progression of PD.

5.1 Outcomes

5.1.1 Primary outcome

The primary goal of this trial is to select the safest and most effective number of repeat doses of allogeneic bone marrow-derived MSC with the aim to slow the progression of PD as determined by the change on the motor section of the MDS-UPDRS (≥ -5) in each active treatment arm versus placebo between screening and 62 weeks follow-up.

5.1.2 Secondary outcomes

Assess 1) safety and tolerability; 2) secondary measurements of clinical efficacy and 3) explore putative mechanisms of action for the effect of MSC

1. MSC therapy will demonstrate safety and tolerability comparable to placebo in terms of serious adverse reactions and immunologic responses.

- New-onset organ failure defined as a significant acute change in the kidney or liver function, leukocytosis, leukopenia or anemia sustained over 3 months; > 75 % reduction in GFR compared to baseline; altered liver function as defined by ALT >150 U/L and or total bilirubin >1.6 mg/dl; or leukopenia defined as < 4K WBC count or anemia as defined by Hgb < 12 for men and < 11 for women.
- Immunologic responses: Donor specific antibodies (DSA) in serum of patients will be measured and compared to baseline to determine if there is a development of antibody response to donor HLA. (Please refer to section 11.2)

2. MSC therapy will improve motor function, disability, quality of life, non-motor symptoms, cognition, and behavioral changes relative to placebo.
   - Motor function: characterized by a change in MDS-UPDRS Total and Motor scores and Timed-Up-and-Go (TUG) from baseline. Immediate response will be assessed at weeks 29, 39, 52, and 78. Normal progression in UPDRS-T is defined by > 8-14 points per year[133-135] Therefore abnormal clinical deterioration is an increase of >12 points per year in the "Off" state. For the UPDRS-M, the literature supports a natural progression of 3-6 points per year[134,136,137] and this equivalent for the MDS-UPDRS motor section[138,139]; therefore, abnormal clinical deterioration is an increase of > 6 points per year. TUG progression is defined as a +/- 3 seconds difference every 6 months.
   - Global measurement of disability as measured by the change in the screening "Off" modified Hoehn and Yahr (H&Y) at the final visit. Expected progression is defined as a < 1.0 increase in H&Y every 2 years[135,140]. Therefore, clinically significant deterioration would be > 0.5 increase in the H&Y over 1-year.
   - Quality of life: based on changes in the modified Schwab and England activities of daily living scale (ADL), Parkinson's Disease Questionnaire 39 (PDQ-39) and EuroQol- 5 Dimension (EQ-5D) from screening and at the final visit. The normal progression is an annual change of 10% in ADL[136] and a change of less than 10% of the PDQ-39 at the final visit compared with the screening visit[141]. Therefore a change of > 10% in ADL or the PDQ-39 over the 1 year study period will be considered clinically significant. For the EuroQol- 5 Dimension (EQ-5D), simulation-based estimates defined the minimally important difference (MID) index score to be between 0.037 and 0.069[142].
   - Non-motor symptoms: The non-motor symptoms questionnaire (NMS) and The University of Pennsylvania Smell Identification Test (UPSIT- 40 odor test booklet).
   - Cognitive function: measured by the change in Montreal Cognitive Assessment (MoCA) from screening to final visit. Normal change is less than 3 points per year. Therefore a ≥ 3 point change over a one year study will be considered clinically significant.
   - Behavioral changes: The Columbia Suicide Severity Rating Scale (C-SSRS), Geriatric Depression Scale-Short Form (GDS-SF), and Parkinson Anxiety Scale (PAS). Suicidal ideation will be assessed by the Columbia Suicide Severity Rating Scale (C-SSRS); any

positive response to any of the questions will be considered significant and would require further investigation. A score of 8 or more in the Geriatric Depression Scale will be considered significant and would require further investigation.

3. Explore putative mechanisms of action for the effect of MSC therapy. We will assess the putative paracrine mechanism of MSCs using neuroimaging and measuring concentrations of cytokines, chemokines, growth factors, neurotransmitters and alpha-synuclein oligomers in the blood (serum or plasma) and CSF.

- Neuroimaging: 3 imaging time points per patient, baseline, week 40, and week 88.
  - Perfusion MRI: We will measure resting cerebral blood flow (CBF) using a pseudo-continuous arterial spin labeling (pCASL) MRI sequence with gradient-echo echo-planar imaging (EPI). Recent ASL studies of PD patients[143] report wide-spread cortical hypoperfusion and, subcortically, hypoperfusion of the caudate nucleus.
  - Diffusion-weighted MRI (for DTI analysis): will be used to examine white matter microstructures, which represent the structural pathways linking specific cortical and subcortical regions. Specifically, we expect to find increased fractional anisotropy and decreased mean diffusivity along white matter fibers that link regions exhibiting increased functional connectivity such as substantia nigra and dorsal striatum.
  - Neuromelanin MRI: neuromelanin-sensitive and iron-sensitive MRI sequences can robustly detect and quantify the neuromelanin loss, and iron accumulation in the substantia nigra pars compacta (SNc) and locus coeruleus (LC). We expect no changes in the neuromelanin (NM) signal in the SNc at 40 weeks.

- Peripheral responses: serum or plasma concentrations of cytokines, chemokines, growth factors, and neurotransmitters will be measured at each follow-up and compared to baseline. Preliminary data generated in our laboratory demonstrated that MSCs effect likely relies on peripheral immune modulation, promoting an anti-inflammatory state.

- Alpha-synuclein oligomers: Total and phosphorylated alpha-synuclein in plasma will be measured using ELISA and total alpha-synuclein in CSF will be measured using Protein Misfolding Cyclic Amplification (PMCA). Kinetic parameters seem to correlate with disease severity measured by H&Y.[144]

6. Study subjects

The target population includes the following characteristics: male or female sex; any ethnic, racial or socioeconomic background; sporadic late-onset PD; PD diagnosed by a neurologist; motor symptoms for 2-10 years and age between 50–79 years old at the start of the trial. Since the prevalence of PD in men is slightly higher than women at a 3:2 ratio, we anticipate that our

recruited population will have a similar gender distribution. A period of 2-10 years from motor symptom onset or disease diagnosis defines a mild to moderate disease stage and medically responsive phase of PD. Our research suggests that peripheral immune response in PD patients, which we hypothesize will be modified by MSC treatment, diminishes 6–10 years after the time of diagnosis. In order to participate, patients must be able to demonstrate ≥ 33% reduction of motor symptoms as determined by the motor subscale of the MDS-UPDRS in the levodopa OFF state vs. the levodopa ON state. The clinical diagnosis must be confirmed by a Movement Disorders Specialist and the PI at the screening. Patients with PD who meet the above criteria must be able to safely tolerate an OFF medicine state as defined by not taking levodopa therapy for 12 hours prior to testing.

Note: At any point during the trial patients will be allowed to receive a Covid-19 vaccine against SARS-CoV-2 approve by the FDA, however, to minimize a confounder effect, this should not be scheduled 8 days prior to an infusion visit or 13 days after an infusion visit.

6.1 Inclusion Criteria

- Men and women between the ages of 50 and 79 years.
- Diagnosis of Parkinson's disease by the UK brain bank criteria including the presence of 1 cardinal signs of PD plus bradykinesia. Diagnosis will be confirmed by the PI or other specialists in Movement Disorders and based on medical history, physical and neurological exams. Patients should have an asymmetric unilateral symptoms onset and supporting features (*See Appendix A for a copy of the UK brain bank criteria*).
- A modified Hoehn and Yahr stage of 3 or less in the levodopa OFF state. (*See Appendix B for a copy of the modified Hoehn and Yahr*)
- Date of Diagnosis of PD between 2 to 10 years, as documented in the medical record.
- Robust response to dopaminergic therapy (defined as greater than or equal to 33% reduction in symptoms (on the MDS-Unified Parkinson's Disease Rating Scale part III) when measured in the ON medicine state compared to OFF state[145].
- If the subject is taking any central nervous system acting medications (e.g., benzodiazepines, antidepressants, hypnotics, stimulants), the regimen must be optimized and stable for 60 days prior to the screening visit.
- A stable Parkinson's disease symptomatic medical regimen therapy for at least 60 days prior to infusion and not projected to require additional Parkinson's disease symptomatic therapy for at least three months after the last infusion.
- Women of childbearing potential will be required to use a reliable form of contraception from 60 days prior to baseline visit until 6 months after the final dose of the study drug.

6.2 Exclusion criteria

- Atypical, vascular, or drug-induced Parkinsonism.

- An atypical DAT scan or MRI supporting an alternative explanation for PD symptoms.
- Presence of any red flags of atypical parkinsonism.
- Patient not on levodopa containing medications or dopamine agonist.
- Unwilling to undergo a Levodopa challenge.
- Clinical features of psychosis or refractory hallucinations.
- A Montreal Cognitive Assessment (MoCA) score of less than 25 (*See Appendix C for a copy of MoCA*).
- Uncontrolled seizure disorder, defined as a seizure within the last 6 months.
- Abnormal Kidney and liver function defined as GFR <45mL/min/m^2, or 2 times the normal range defined by the laboratory for ALT and AST..
- Presence of anemia defined as: Hgb <12g/dl for men and <11g/dl for women or Hct < 38% for men and < 34 % for women.
- Presence of clinically refractory orthostatic hypotension at the screening visit.
- Body mass index of ≥ 40. This cut off is based on the high likelihood of a traumatic LP at this BMI and/or failure to obtain an LP.
- Cardiac disease: History of congestive heart failure, clinically significant bradycardia, presence of 2^{nd,} or 3rd-degree atrioventricular block.
- Pulmonary disease: COPD with oxygen-requirement at rest or with ambulation; or moderate to severe asthma.
- Active malignancy under treatment within 2 years prior to the screening visit, except for cancer in situ NXMX (TNM staging).
- Any current suicidal ideation or behaviors defined by 1 or more positive answers on the Columbia Suicide Severity Rating Scale (C-SSRS) in the last month.
- Active autoimmune disorder or immunocompromised state, including chemotherapy administration within the last three years or current immunosuppression as defined by WBC <3 x 10^3 cells/ml.
- History of medium or large size vessel cerebrovascular accidents.
- History of traumatic brain injury with loss of consciousness and residual neurologic symptoms.
- Major surgery within the previous 3 months or planned in the ensuing 6 months.
- Clinically significant abnormalities in the Screening visit laboratory studies.
- History of use of an investigational drug 4 months prior to the screening visit.
- Currently enroll in an interventional clinical trial.
- History of brain surgery for PD.
- History of stem cell treatment for any indication within 3 years prior to the screening visit.
- Unable to return for follow-up visits for clinical evaluation, laboratory studies, or imaging evaluation.
- Unable to obtain peripheral IV access at the screening visit.
- Not willing to allow physical exam videotaping.
- Refusal or inability to undergo the lumbar puncture.

- Refusal or inability to undergo the MRI procedures.
- Substance abuse disorder.
- Active anticoagulation treatment and/or abnormal INR (with the exception of Aspirin).
- Any other condition that the investigator feels would pose a significant hazard to the patient if enrolled or complicate the study assessments.

6.3 Selection and enrollment of Subjects

6.3.1 Advertisement

Advertisement will be focus on the following strategies:

a) Websites: Place trial information on 3 different websites, including clinical trials.gov, Research Match, and Fox Trial Finder website.
b) UTHealth Patients: we will leverage electronic health records and electronic databases from UTHealth to reach possible candidates. We will also contact our study subjects from previous studies.
c) Clinic and Hospital outreach: Informational flyers will be placed in the waiting room of the movement disorders and general neurology clinics at UTHealth (TMC, Woodlands and Harris Health Smith clinic).
d) Inform Colleagues: The PI will send an email to all the neurologists at UTHealth, Baylor College of Medicine, St Luke's and Methodist. This email will include a short description of the study and the set of materials designed for physicians.
e) Out-Reach to the Community: Flyers will be distributed at different events for patients, including the ones organized by HAPS and other support groups.

6.3.2 Pre-screening:

After contacting potential candidates and getting their approval, we will send them a pre-screening questionnaire (CPHS approved) to answer and return via a pre-paid envelope. The questionnaire provides information on the patient's diagnosis, their response to medicines, laboratory tests (no older than a year), neuroimaging results, and their current medical history. This will allow us to develop a list of appropriate candidates to screen, maximizing our recruiting time, and reducing screen failures. We will contact the responders via email or phone one month after receiving the questionnaires to inform them of a decision to consider them as potential participants in the study. If a patient is considered a good candidate, a copy of the consent will be emailed to them, and a week later, the research coordinator will contact the patient to have an initial discussion about the consent. Afterward, a screening visit will be scheduled in which a longer and more in-depth discussion of the consent would take place before starting any study procedures.

6.4 Estimated number of Subjects

Our goal is to recruit and retain 45 patients, with each patient finishing the one-year follow-up after the last injection and complete the study. In order to achieve this goal, we estimate the need to recruit 50 patients, which assumes a 10% projected screen failure rate.

6.5 Method of Assigning Subjects to Study Drug

Subjects will be assigned to any of the three treatment arms randomly on ratio 1:1:1 by computer table randomization method to yield a total of up to 45 randomized treated subjects; 15 subjects per arm. Screen failures or subjects who exit the study prior to the administration of the first treatment dose may be replaced with additional subjects, which could cause an overall number of subjects randomized to be greater than the 45 anticipated. However, no more than 45 subjects will be treated.

7. Study intervention: Mesenchymal stem cell therapy

7.1 Allogeneic mesenchymal stem cells

Bone marrow extraction from a healthy donor (21 CFR Parts 1271 Subpart C) will be obtained by aspiration under local anesthesia at Memorial Hermann Hospital. Testing will be performed using FDA-approved licensed kits by Gulf Coast Regional Blood Center within seven days of collection of the marrow. Mesenchymal stem cells will be expanded 4 passages using a Terumo Quantum Bioreactor[146] by the Center for Cell and Gene Therapy of Baylor College of Medicine under Current Good Manufacturing Practices designated by the FDA. Thawing will be initiated on the planned date of administration, the cells will go through the tests described in the manufacturing section and will be placed in 5% buminate Fenwal transfer packs routinely used in blood banks. Bags will be covered with an opaque cloth, so differences in the contents cannot be appreciated. The concentration of the infusion will be 1.5 X 10^7/ ml. The cells will be transported from the CAGT laboratory to the Memorial Hermann hospital clinical research unit (CRU) in certified coolers at 4°C-10° C, this is an approximately 15 minutes transport that via routes that link the Texas Medical Center institutions. Upon receipt, the cell packs will be examined for integrity and appearance, and this will be documented on the administrative paperwork. (*For a sample of labeling see Appendix D*).

7.2 Placebo

Placebo will be identical to the investigational product but will not contain MSCs. Placebo will be constituted by a 5% buminate solution. All bags will be covered with an opaque cloth, so differences in the contents cannot be appreciated.

7.3 Delivery route and infusion rate

A study drug or placebo will be supplied to each one of the patients as an intravenous infusion. Antecubital vein access will be used for the infusion with 18 or 20 gauge catheter tubing. The access will be initially primed with normal saline; thereafter the study drug will be infused at a rate of 2 ml/min followed by a flush with 25ml of normal saline. This rate may be modified dependent on the patient's response. (*As detailed in Section 11.1.2*)

7.4 Facility: Clinical Research Unit

The infusion will take place in the clinical research unit (CRU) located on the third floor, Robertson Pavilion 352 at the Hermann Memorial Hospital in the Texas Medical Center. This facility provides a team of research nurses, study coordinators, and lab specialists. Their services include outpatient and inpatient rooms, diagnostic tests and procedures, regulatory monitoring, critical or emergent response access, and lab services 24 hours a day.

8. Study assessments

8.1 Demographics and complete medical history

Subjects will provide information on their demographics, complete medical history including social, smoking, alcohol and caffeine usage, as well as concomitant prescribed or OTC medication usage including date started, dose, and frequency. (*See Appendix E for the medication form*)

<u>Allowed Concomitant Medications</u>

Provided they were in use for at least 60 days prior to infusion, a stable regimen of central nervous system acting medications is allowed. These include benzodiazepines, antidepressants, hypnotics, anti-psychotic, and dopaminergic therapy (all levodopa formulations, dopamine agonists, amantadine, anticholinergic agents, and MAO-B inhibitors). Any modifications to the medical regimen will be determined by the PI.

<u>Medications or Supplements Not Allowed</u>

The regular use of over the counter medications other than a standard daily multivitamin, pain medication, antihistamines, and cold medications or any experimental medication is not allowed.

8.2 Vital signs, orthostatic changes, weight and height

Blood pressure (supine and standing), heart rate (supine and standing), respiration, oxygen saturation, and body weight (in kg) will be measured during most of the visits. Height (in cm) will be measured at the screening visit only.

8.3 Physical and neurological examination

A complete physical and neurological examination will be performed and recorded at every clinical visit. The neurologic exam will include orientation to self, time and place, speech observation, cranial nerves, Global Bradykinesia, dexterity, tremor, muscle tone, strength, sensory, reflexes, cerebellar function, Retropulsive pull test, Romberg's test, and tandem gait.

8.4 Laboratory Test

- Complete blood count (CBC) with differential: hematocrit, hemoglobin, platelet count, RBC indices, Total RBC, Total WBC, and WBC & differential. Additionally, for CSF analysis protein and glucose.
- Comprehensive Metabolic Panel: albumin, total protein, Alkaline phosphatase (ALP), Alanine aminotransferase (ALT), Aspartate aminotransferase (AST), Blood urea nitrogen (BUN), Calcium, Carbon dioxide, Chloride, Creatinine, Glucose, Potassium, Sodium, Total bilirubin
- Specialty Blood Chemistry: INR, PT, PTT, and dipstick urinalysis.

8.5 Rating scales

8.5.1 MDS-UPDRS (Movement Disorder Society Unified Parkinson's disease Rating Scale)

The MDS-UPDRS serves as a disability and impairment scale for progression; it is divided into four sections:

- Part I: Non-Motor Aspects of Experiences of Daily Living (13 questions).
- Part II: Motor Aspects of Experiences of Daily Living (M-EDL) (13 questions).
- Part III: Motor examination (18 questions total, 9 of those with bilateral assessment of different body).
- Part IV: Motor complications (6 questions).

All items have 5 response options with uniform anchors of: 0 = normal, 1 = slight (symptoms/signs with sufficiently low frequency or intensity to cause no impact on function), 2 = mild (symptoms/signs of frequency or intensity sufficient to cause a modest impact on function, 3 = moderate (symptoms/signs sufficiently frequent or intense to impact considerably, but not prevent function), 4 = severe (symptoms/signs that prevent function).
The motor section of the MDS- UPDRS will be performed by a qualified Movement Disorder Specialist.

8.5.2 Modified H&Y (Hoehn and Yahr scale)

The Modified Hoehn and Yahr Scale is a staging instrument that defines 8 broad categories of motor function in Parkinson's disease, starting at Stage 0: no signs of disease to the highest stage 5: wheelchair bound or bedridden unless aided. The principal investigator will conduct the assessment at each clinical visit (See Appendix B for a copy of the Modified Hoehn and Yahr Scale).

8.5.3 TUG (Timed Up and Go Test)

Time in seconds required to stand from a chair, walk 7m, turn, walk back to the chair and sit down. The principal investigator will conduct the assessment at each clinical visit.

8.5.4 ADL (Modified Schwab and England Activities of daily living score)

The Schwab & England scale is a physician assessment of the subject's level of independence. The subject will be scored on a percentage scale reflective of his/her ability to perform acts of daily living in relation to what he/she did before Parkinson's disease appeared. Scores range from 0% to 100% in increments of 10%, where 100% is completely independent and 0% is only vegetative functions. The principal investigator will conduct the assessment at each clinical visit (*See Appendix F for a copy of the ADL*).

8.5.5 PDQ-39 (Parkinson's Disease Questionnaire)

The PDQ-39 is a 39 questions Parkinson's disease self-completed that assess Parkinson's disease-specific health related quality over the last month. Thirty nine multiple-choice items covering 8 dimensions: mobility (10 items), activities of daily living (6 items), emotional well-being (6 items), stigma (4 items), social support (3 items), cognition (4 items), communication (3 items) and bodily discomfort (3 items). All items are assumed to impact QoL and must be answered to compute scores for each dimension. Questions are answered based on experiences from the preceding month using a 5-point ordinal scoring system: 0 = never, 1 = occasionally, 2 = sometimes, 3 = often, 4 = always. Lower scores reflect better quality of life.

Dimension score = sum of scores of each item in the dimension divided by the maximum possible score of all the items in the dimension, multiplied by 100. Each score range from 0 = never have difficulty to 100 = always have difficulty his calculation provides a percentage score ranging between 0 and 100. Overall score can be summarized as the Parkinson's Disease Summary Index (PDSI). PDSI sum of dimension total scores divided by 8. This calculation provides a percentage score ranging between 0 and 100. The higher the percentage, the higher the disease impact on the quality of life. (*See Appendix G for a copy of the PDQ-39*).

8.5.6 EuroQol- 5 Dimension (EQ-5D-5L)

The EQ-5D-5L is a self-assessed, health-related, quality of life questionnaire. The scale measures quality of life on a 5-component scale, including mobility, self-care, usual activities, pain/discomfort, and anxiety/depression. Each level is rated on a scale that describes the degree of problems in that area (i.e., I have no problems walking about, slight problems, moderate problems, severe problems, or unable to walk). This tool also has an overall health scale where the rater selects a number between 1-100 to describe the condition of their health, 100 being the best imaginable. (*See Appendix H for a copy of The EQ-5D-5L*).

8.5.7 MoCA (Montreal Cognitive Assessment)

The Montreal Cognitive Assessment is a rapid screening instrument for working memory, visual-spatial abilities, executive function, attention, concentration, language, and orientation. The total score ranges from 0 to 30; a score of 26 or above is considered normal, 18-25 = mild cognitive impairment (MCI), 10-17= moderate cognitive impairment (Alzheimer's disease) and less than 10= severe cognitive impairment. MoCA will be administered at the screening visit and at the final visit by the principal investigator (*See Appendix C for a copy of the MoCA*).

8.5.8 UPSIT (University of Pennsylvania Smell Identification Test)

The University of Pennsylvania Smell Identification Test is a comprehensive 40-item self-administered olfactory test that provides an absolute indication of smell loss (anosmia; mild, moderate, or severe microsmia). The kit consists of four booklets, each containing ten questions with a total score of 40. The score is compared to scores in a normative database from 4000 normal individuals (according to their age group and gender).

8.5.9 C-SSRS (The Columbia Suicide Severity Rating Scale)

The Columbia–Suicide Severity Rating Scale (C-SSRS) is an assessment tool that evaluates suicidal ideation and behavior. It rates an individual's degree of suicidal ideation on a scale, ranging from "wish to be dead" to "active suicidal ideation with specific plan and intent". A study member will conduct the assessment at screening using the Lifetime/Recent version and the Since Last Visit version at each safety/clinical visit. (*See Appendix I and J for the Lifetime/Recent version and the Since Last Visit version of the C-SSRS*).

8.5.10 Geriatric Depression Scale Short form (GDS-SF)

The Geriatric Depression Scale Short form (GDS-SF) is a 15-item screening tool that is used to identify depression in older adults. A score of 0 to 5 is normal. A score greater than 5 suggests depression. (*See Appendix K for a copy of the Geriatric Depression Scale Short form*)

8.5.11 Parkinson Anxiety Scale (PAS)

The Parkinson Anxiety Scale (PAS) is an anxiety measure scale for use in PD patients. This is a

12-item patient-rated scale with three subscales, subscales for persisting anxiety (5 items), episodic anxiety (4 items), and avoidance behavior (3 items). Items are a score on a 5-point Likert scale, with 0 = not or never, 1 = very mild or rarely, 2= mild or sometimes, 3= moderate or often and 4= severe or almost always (*See Appendix L for a copy of The Parkinson Anxiety Scale*).

8.5.12 RBD-Single-Question Screen (RBD1Q)

Consists of a single question, answered "yes" or "no," as follows: "Have you ever been told, or suspected yourself, that you seem to 'act out your dreams' while asleep (for example, punching, flailing your arms in the air, making running movements, etc.)?"

A positive answer is suggestive of REM Behaviour Disorder (RBD). RBD1Q can detect RBD with 94% sensitivity and 87% specificity (*See Appendix M for a copy of the RBD-Single-Question Screen)*.

8.6 Donor-specific antibodies (DSAs)

As these MSC are derived from third party donor cells, we cannot discount the possibility of generation of recipient anti-donor alloimmune responses. The donor MSC will be haplotyped to determine the HLA gene expression on these cells before they are injected. In order to monitor the development of immune response directed against the donor MSC, we will perform Donor-specific antibodies in between infusions and 6 months and 12 months after the final infusion. HLA antibody level will be assessed using One Lambda (West Hills, CA) LABScreen Single Antigen Bead (SAB) Assay evaluated on either a Luminex 200 or LabScan 3D (instruments correlated for clinical use). The development of alloantibody directed against donor MSC will be identified by a positive response post-MSC injection. In case of sensitization patients will enter a modified schedule for their infusions (*As detailed in section 11.2.1)*

8.7 Peripheral markers

Serum and plasma samples will be stored at −80°C until further analysis. To determine the proposed biological markers listed in *Table 3*, a Millipore Milliplex MAP® 37-plex human cytokine/chemokine panel and Milliplex MAP Human Neurodegenerative Disease Magnetic Bead Panel 3 (EMD Millipore, Billerica, MA, USA) will be used.

Table 3. Peripheral and CNS Molecular Markers						
Growth Factors	Inflammation	Chemotaxis	Lymphocytes	Neurotransmitters & metabolites	Oxidative stress markers	Physiological Markers
VEGF, BDNF, GDNF	IL-1β, IL-2 IL-6, IL-17, TNFα, COX-2, PGE-2	CCL2 (MCP-1), CCL7 (MCP-3), CCL11 (Eotaxin) CCL22 (MDC), CXCL10 (IP-10), CX3CL1 (Fractalkine)	IFNγ, TGFβ, IL-4, IL-10	5-hydroxytryptamine, Homovanillic acid	Total antioxidant capacity	NfL mir-7

8.8 Lumbar Puncture

The same biological markers, as proposed in the peripheral markers, will be assessed in the spinal fluid at baseline and week 49. A qualified neurologist will perform a bedside lumbar puncture under sterile procedural guidelines; approximately 15-20 ccs of CSF will be collected and will take approximately 30-60 minutes. Afterward, the subject will remain recumbent and in observation at the clinical research unit (Part of the Memorial Hermann hospital) for 2 hours following the procedure, with fluid intake encouraged.

8.9 Peripheral CSF and urine α-synuclein oligomers:

CSF,plasma and urine will be used to assess peripheral α-synuclein oligomers. Total and phosphorylated alpha-synuclein in plasma will be measured using ELISA and Total alpha-synuclein in CSF and urine will be measured using Protein Misfolding Cyclic Amplification (PMCA)[147]. There is published data stating that when preformed a-synuclein aggregates are incubated with MSC-conditioned medium, a-synuclein aggregates become disassembled, and insoluble and oligomeric forms of a-synuclein are markedly decreased[103]. In addition, kinetic parameters correlated with disease severity measured by H&Y[144].

8.10 Neuroimaging

We will include 3 imaging time points per patient, baseline, week 40, and week 88.

Imaging will be collected using a 3 tesla Philips Ingenia MR scanner. A T1-weighted magnetization-prepared rapid acquisition turbo field echo sequence will be collected (repetition time/echo time TR/TE] = 8.4/3.9 ms; flip angle = 8 degrees; matrix size = 256× 256; field of view = 240 mm; slice thickness = 1.0 mm, sagittal acquisition). A T2-weighted turbo spin-echo volume acquisition (TR/TE = 2500/367 ms; echo train length 120, pixel bandwidth 380, flip angle = 90 degrees, matrix size = 256 × 256; field of view = 240 mm, slice thickness = 0.94 mm, 186 sagittal slices) will also be collected. A fluid-attenuated inversion recovery (FLAIR) volume will also be collected (repetition time/echo time TR/TE = 4800/323 ms; inversion time = 1650 ms; echo train length = 182; pixel bandwidth = 957; flip angle = 90 deg; matrix size = 256 x 256; isotropic 1 mm voxels). Detailed structural MRI analysis will be carried out according to Ellmore et al 2010[148].

- Perfusion MRI**:** We will measure resting cerebral blood flow (CBF) using a pseudo-continuous arterial spin labeling (pCASL) MRI sequence with gradient-echo echo-planar

imaging (EPI). Arterial spin-labeled (ASL) perfusion MRI allows for noninvasive quantification of CBF as part of a multimodal MRI examination. It is less invasive, less costly, and more widely available than radionuclide methods like Xe-133 clearance or O^{15} positron emission tomography. Critical parameters our acquisition protocol include the following TR/TE =4000/17 msec, flip angle=90 degrees, slice thickness = 5 mm, 240 mm field of view, slice gap = 1.0 mm, voxel resolution = 2.75x2.75x6 mm^3, 30 dynamic signal averages, with a labeling duration of 1650 msec and a post-labeling delay of 1600 msec. ASL data processing and CBF calculation will be performed in AFNI. Raw ASL images will be co-registered to each subject's high-resolution T1-weighted MRI. The T1 MRI will be segmented into gray, white, and CSF binary masks and sampled to the resolution of the raw ASL images. After motion correction, the ASL images will be multiplied by the gray and white matter masks, and global cortical gray matter and white matter images will be generated. Pairwise subtraction images (label minus control) will be generated for each dynamic after smoothing by an 8 mm full-width half-maximum kernel. Averaged difference images will be converted to ml/100g/min gray matter and white matter CBF maps using a single-compartment model [149].

- Diffusion-weighted MRI (for DTI analysis)**:** A set of diffusion-weighted image volumes (32-directions, high angular resolution) will be collected using the gradient overplus option with one B0 (non-diffusion weighted) image volume acquired before the acquisition of one repetition of the diffusion-weighted scans (TR/TE = 8500/67 ms; FA =90 deg; matrix size 128 x 128; FOV = 224 mm; 2 mm thick axial slices, b-value of 800 s/mm^2). Diffusion tensor and associated computations will be done as specified in Ellmore et al. 2014[150]. Diffusion tensor imaging will be used to examine white matter microstructures, which represent the structural pathways linking specific cortical and subcortical regions.
- Neuromelanin MRI: We will use a 2D gradient response echo sequence with magnetization transfer contrast (2D GREMT)[151] with the following parameters: repetition time (TR) = 260 ms; echo time (TE) = 2.68 ms; flip angle = 40°; in-plane resolution = 0.39 × 0.39 mm2; partial brain coverage with field of view (FoV) = 162 × 200; matrix = 416 × 512; number of slices = 10; slice thickness = 3 mm; slice gap = 0 mm; magnetization transfer frequency offset = 1,200 Hz; number of excitations (NEX) = 8; acquisition time = 8.04 min. The slice-prescription protocol will consist of orienting the image stack along the anterior-commissure–posterior commissure line and placing the top slice 3 mm below the floor of the third ventricle, viewed on a sagittal plane in the middle of the brain. This protocol will provide coverage of SN-containing portions of the midbrain (and cortical and subcortical structures surrounding the brainstem) with high in-plane spatial resolution using a short scan easy to tolerate by clinical populations. Whole-brain, high-resolution structural MRI scans will also be acquired for preprocessing of the 2D GRE-MT (NM-MRI) data: a T1-weighted 3D BRAVO sequence (inversion time = 450 ms, TR ~ 7.85 ms, TE ~ 3.10 ms, flip angle = 12°, FoV = 240 × 240, matrix = 300 × 300, number of slices = 220, isotropic voxel size =

0.8 mm3) and a T2-weighted CUBE sequence (TR = 2.50 ms, TE ~ 0.98 ms, echo train length = 120, FoV = 256 × 256, number of slices = 1, isotropic voxel size = 0.8 mm3).

The anatomical imaging (T1- and T2- weighted and Fluid Attenuated Inversion Recovery) data allow us to see brain structure at the level of about 1 cubic millimeter. We will use these scans to estimate the volume of subcortical structures (e.g., putamen, caudate nucleus) to measure the cortical thickness (e.g., gray matter density), and detect any gross structural changes or inflammation that appear during treatment. These structural MRI scans are also necessary to provide co-registration templates (to correct for movement between scans) for the ASL, DTI, and neuromelanin sequences described above.

9. Potential Risks:

Lumbar puncture: There is some discomfort during the lumbar puncture procedure. After a brief sting from the numbing medication, the subject will feel mostly pressure or occasionally tingling sensations. The most common side effects of a lumbar puncture are headache, nausea, vomiting, or fever. In our experience, approximately 1 % of patients undergoing a lumbar puncture can develop a refractory headache (positional). Most post LP headaches respond to lying flat and resting for 48 hours and drinking plenty of fluids and caffeinated beverages as tolerated. By definition, they are refractory if they fail to resolve after these measures are taken and an autologous blood patch performed by anesthesia or neuroradiology is the treatment of choice for immediate resolution.

Blood draw: Blood draws, and infusions may cause discomfort, pain, and bruises at the site where blood is taken and sometimes causes people to feel lightheaded or to faint.

MRI scanning: All participants will be screened for metallic implants incompatible with the MRI environment. If a patient experiences unbearable claustrophobia inside MRI scanners, he/she will be removed from the scanner.

Cognitive, neurologic, and physical exams and history: These are administered by medical professionals. However, there may be some subjects embarrassed or anxious due to the questions that must be answered.

Acute immune reactions: The PI will ensure that appropriate best clinical practices and treatment are initiated should an event indicating an allergic or hypersensitive reaction occur. If this occurs, the infusion will be stopped immediately, and the subject closely monitored until stable. The suggested treatment will depend upon the grading of the reaction (NCI-CTCAE criteria). The PI will have the support of the emergency response teams from the hospital as needed. After the infusion, patients will be continuously monitored for 4 more hours, and a full neurological physical examination will be performed before discharging the patients. In case of a severe adverse event,

we can get an assessment in regards to the continuation of the study from our DSMB within 24-48 hours.

Confidentiality: There is a possible risk of breach of confidentiality. Records with the subject's name will be maintained in the CRU charts. A database with identifiers will be maintained by the coordinator for scheduling purposes only, and will not contain any study results. All data will be kept in a password protected Redcap database. The tapes with the MDS-UPDRS section III will be kept in a locked office and the patients/subjects are not identified by name or any other identifying features.

10. Study schedule

The study will last a total of one year and 8 months (88 weeks) per patient (3 groups) and will include 11 clinical visits and 10 telephone evaluations (*Please see section 10.3 for the study timeline and 10.4 for study schedule*).

There are 6 types of clinical visits: Screening, Baseline, Infusion, Safety and Clinical assessment, unscheduled visit, and final visit plus phone call evaluations starting after the first infusion.

- Visit #1 Screening Visit (between week -12 to week -3): after signing the consent and discussing the purpose and timeline of the study, the PI will confirm the PD diagnosis and establish the current disease stage. At this visit, the CRU staff will collect blood for laboratory assessment. The purpose of this visit is to ensure that the patient meets the inclusion and exclusion criteria.
- Visit #2 Baseline visit (between week -11 to week-1): the purpose of this visit to obtain baseline measurements on serum, plasma, CSF, and neuroimaging.
- Visit #3 Infusion Day (week 0): First IV MSCs infusion.
- Visit #4 Clinical and Safety assessment (week 9+/-1.5 week): the purpose of this visit is to determine the safety and the immediate immunological, physical and neurological response to the first infusion.
- Visit #5 Infusion Day (week 18+/-1 week): Second IV MSCs infusion.
  Visit #6 Clinical and Safety assessment (week 27+/-1.5 week): the purpose of this visit is to monitor safety and the immunological, physical and neurological response to the second infusion.
- Visit #7 Infusion Day (week 36+/-1 week): Third IV MSC infusion
- Visit #8 Clinical and Safety assessment (week 40+/-1.5 week): the purpose of this visit is to monitor the safety and the immunological, physical and neurological response to the third infusion. Additionally, to collect enough data for our first interim analysis.

- Visit #9 Clinical and Safety assessment (week 49+/-1.5 week): the purpose of this visit is to monitor the safety and the immunological, physical, and neurological response to the infusions.
- Visit #10: Clinical and Safety assessment (week 62+/-1.5 week): the purpose of this visit is to monitor the safety and the immunological, physical, and neurological response to the infusions.
- Visit # 11: Final visit (week 88+/-1.5 week): the purpose of this visit is to determine the physical and neurological response to the complete MSC treatment.
- Unscheduled visit (between 2 to 15 days after reporting a non-live treating AE related to the MSC infusion): the purpose of this visit is to fully assess any adverse event or any other condition that the principal investigator considers vital to assess.
- Telephone or email evaluations (+/- 7 days): The purpose of this evaluation is to assess and document AEs. These will occur at week 1, week 4, week 13, week 19, week 23, week 31, week 38, week 444, week 55, and week 75.

10.1 Clinical visits

10.1.1 Screening visit: (*Visit 1*= between weeks -12 and -3)

Patients will be screen in numerical order based on their assigned number. This visit will take approximately 3-4 hours.

Prior to performing any study activity, the subject will be thoroughly informed on all aspects of the study, including all scheduled visits, activities, and procedures, and will be requested to sign and date the IRB approved informed consent form.

The following procedures and evaluations are to be performed at Screening visit:

- Obtain written informed consent.
- Subject ID Number assigned (2 digits number starting at 01)
- Obtain Vital Signs (orthostatic blood pressure, respiration, oxygen saturation, heart rate, temperature, weight, height).
- Obtain a 12 lead Electrocardiogram (EKG)
- Obtain Demographic information.
- Medical History.
- Obtain socio-economic information.
- Obtain smoking, alcohol & caffeine information.
- Review and document concomitant medications.
- Document PD diagnosis date and PD Features.
- Assess primary diagnosis/probability of PD.
- Perform general Physical Exam: general appearance, head, eyes, ears, nose, neck, and throat, lungs, heart, peripheral pulses, and skin.

- Perform a complete neurological exam: orientation to self, time and place, speech observation, cranial nerves, strength, sensory, reflexes, gait, and cerebellar.
- Perform the MDS-UPDRS (Movement Disorders Society Unified PD Rating Scale). The MDS-UPDRS scale will be performed first OFF medication and then ON medication (For this assessment, patients will be asked to hold their current PD treatment for 12 hours prior to the visit). The MDS- UPDRS-III will be videotaped for rating assessment by the other two movement disorder specialists.
- Assess H&Y (Hoehn and Yahr scale).
- Measure TUG (Timed Up and Go Test).
- Administer MoCA (Montreal Cognitive Assessment).
- Provide UPSIT (University of Pennsylvania Smell identification test) for self-administration.
- Provide ADL (Schwab and England Activities of daily living score) for self-administration.
- Administer the Lifetime/Recent version of the Columbia–Suicide Severity Rating Scale (C-SSRS).
- Provide EQ-5D-5L (EuroQol- 5Dimension).
- Provide GDS-SF (Geriatric Depression Scale Short form).
- Provide PAS (Parkinson Anxiety Scale).
- Provide PDQ-39 (Parkinson's Disease Questionnaire) for self-administration.
- Provide RBD-Single-Question Screen (RBD1Q).
- Urine sample for Dipstick urinalysis.
- Collect a blood sample for laboratory screening:
  - ➢ Complete blood count (CBC) with differential.
  - ➢ Comprehensive Metabolic Panel.
  - ➢ Specialty Blood Chemistry.
- Vascular access assessment
- Lumbar access assessment
- Assess the need for change in symptomatic therapy.

A week after this visit, the principal investigator will assess subjects for study eligibility based on all inclusion/exclusion criteria, including the laboratory results and will determine if the patient will be enrolled in the study.

10.1.2 Baseline visit (*Visit 2*= between week -11 to week-1)

This visit will take approximately 6-7 hours. The following procedures and evaluations are to be performed at baseline:

- Performed Neuroimaging in the levodopa OFF state:
  - ➢ High-resolution T1- & T2-weighted MRI.
  - ➢ Perfusion MR.

- Diffusion-weighted MRI.
- Neuromelanin MRI.
- Collect blood samples for:
  - DSA (Donor specific antibodies). Order as HLA typing.
  - Peripheral markers: Neurotransmitters, Oxidative stress, cytokines, chemokines, and growth factors.
  - α-synuclein oligomers levels.
- Collect urine sample for α-synuclein oligomers
- Performed Lumbar Puncture and observe for 2 hours.
  CSF samples for:
  - CBC with differential.
  - Glucose and Protein levels
  - α-synuclein oligomers
  - Markers: Neurotransmitters, Oxidative stress, cytokines, chemokines and growth factors.
- Provide verbal and written instruction regarding the infusion visit and explain the procedure to the patient.
- Obtain vital signs before discharge (blood pressure, respiration, oxygen saturation, heart rate, and temperature).

10.1.3 Infusion visits: (*Visit 3*= Week 0; *Visit 5*=Week 18+/-1 week; *Visit 7*=Week 36+/-1 week)

This visit will take approximately 5-6 hours. The following procedures and evaluations are to be performed at Infusion visit:

- Explain the procedure to the patient, including the 4-hour monitoring period.
- Obtain Vital Signs (blood pressure, respiration, oxygen saturation, heart rate, and temperature).
- If the urine sample was not collected during the baseline, please collect the sample at this visit.Review and document concomitant medications
- Perform general Physical Exam: general appearance, head, eyes, ears, nose, neck, and throat, lungs, abdomen, heart, peripheral pulses and skin.
- Perform a complete neurological exam: orientation to self, time and place, speech observation, cranial nerves, strength, sensory, reflexes, gait, and cerebellar.
- Infusion procedure: The study patient will take their regular PD meds on the infusion day and arrive by 8:00 am. After obtaining vital signs and performing a general and neurological examination, two IV lines will be started. MSCs will be infused according to the description provided in section 7.3. Vitals signs will be obtained at the end of the infusion.

- 4-hour monitoring period: After the infusion patients will be monitored for 4 hours. Vital signs will be checked 1 hour after the infusion, 2 hours after the infusion, and before discharge**.** A complete physical and neurological exam will be done before discharge.
- Provide written information regarding red flags. (*See Appendix N for a copy of Red Flags*).

Subjects will be instructed to immediately report any adverse events to the Coordinator after their departure.

10.1.4 Clinical and Safety Assessment: (*Visit 4* = week 9+/-1.5 week; *Visit 6* = week 27+/-1.5 week and *Visit 8* = week 40+/-1.5week; *Visit 9*=week 49+/-1.5 week; *Visit 10*=week 62+/-1.5 week)

This visit will take approximately 3-4 hours. The following procedures and evaluations are to be performed at Clinical and Safety Assessment visits:

- Obtain Vital Signs (orthostatic blood pressure, respiration, oxygen saturation, heart rate, temperature, weight). (All visits except visit 9)
- Review and document concomitant medications.
- Perform general Physical Exam: general appearance, head, eyes, ears, nose, neck, and throat, lungs, heart, peripheral pulses and skin.
- Perform a complete neurological exam: orientation to self, time and place, speech observation, cranial nerves, strength, sensory, reflexes, gait, and cerebellar.
- Assess MDS-UPDRS (Movement Disorders Society Unified Parkinson's disease Rating Scale). The MDS-UPDRS-III will be videotaped for rating assessment by the other two movement disorder specialists. (All visits except visit 9)
- Assess H&Y (Hoehn and Yahr scale). (All visits except visit 9)
- Assess TUG (Timed Up and Go Test). (All visits except visit 9)
- Administer MoCA (Montreal Cognitive Assessment). (***Only at visit 9***)
- Provide UPSIT (University of Pennsylvania Smell identification test) for self-administration. (***Only at visit 9***). This will be sent to the patient home address one week before visit 9. Each patient will complete the test at home and bring it for visit 9.Administer the Since last time version of the Columbia–Suicide Severity Rating Scale (C-SSRS). (All visits except visit 9)
- Provide ADL (Schwab and England Activities of daily living score) for self-administration.
- Provide EQ-5D-5L (EuroQol- 5Dimension). (All visits except visit 9)
- Provide GDS-SF (Geriatric Depression Scale Short form). (All visits except visit 9)
- Provide PAS (Parkinson Anxiety Scale). (All visits except visit 9)
- Provide PDQ-39 (Parkinson's Disease Questionnaire) for self-administration. (All visits except visit 9)
- Collect blood samples for:
  - ➢ DSA (Donor specific antibodies). Order as HLA typing. (***All visits except visit 9 & 10***)

- ➢ Peripheral markers: Neurotransmitters, Oxidative stress, cytokines, chemokines, and growth factors. (***All visits except visit 10***)
  - ➢ α-synuclein oligomers levels. (***Only at visit 8 &9***)
- Performed Neuroimaging in the levodopa OFF state ***(Only at visit 8)***:
  - ➢ High-resolution T1- & T2-weighted MRI.
  - ➢ Perfusion MR.
  - ➢ Diffusion-weighted MRI.
  - ➢ Neuromelanin MRI.
- Performed Lumbar Puncture and observe for 2 hours ***(Only at visit 9)***
- Collect urine sample for α-synuclein oligomers ***(Only at visit 9)***
- CSF samples for:
  - ➢ CBC with differential.
  - ➢ Glucose and Protein levels
  - ➢ α-synuclein oligomers
  - ➢ Markers: Neurotransmitters, Oxidative stress, cytokines, chemokines, and growth factors.
- Review DSA results from the previous visit. ***(Only at visit 4 & 6)***:
- Assess the Need for change in symptomatic therapy.

10.1.5 Final visit: (*Visit 11*= Week 88 +/-1.5 week) OR Early termination visit

The following procedures and evaluations are to be performed at the final visit:

- Obtain Vital Signs (orthostatic blood pressure, respiration, oxygen saturation, heart rate, temperature, weight).
- Review and document concomitant medications.
- Perform general Physical Exam: general appearance, head, eyes, ears, nose, neck, and throat, lungs, heart, peripheral pulses and skin.
- Perform a complete neurological exam: orientation to self, time and place, speech observation, cranial nerves, strength, sensory, reflexes, gait, and cerebellar.
- Assess changes in diet, physical activity and sleep patterns during the study.
- Asses Covid-19 disease during the study
- Assess MDS-UPDRS (Movement Disorders Society Unified Parkinson's disease Rating Scale). The MDS-UPDRS-III will be videotaped for rating assessment by other two-movement disorder specialists.
- Assess H&Y (Hoehn and Yahr scale).
- Assess TUG (Timed Up and Go Test).
- Administer MoCA (Montreal Cognitive Assessment).
- Provide UPSIT (University of Pennsylvania Smell identification test) for self-administration.
- Administer the Since last time version of the Columbia–Suicide Severity Rating Scale (C-SSRS).

- Provide ADL (Schwab and England Activities of daily living score) for self-administration.
- Provide EQ-5D-5L (EuroQol- 5Dimension).
- Provide GDS-SF (Geriatric Depression Scale Short form).
- Provide PAS (Parkinson Anxiety Scale).
- Provide PDQ-39 (Parkinson's Disease Questionnaire) for self-administration.
- Provide RBD-Single-Question Screen (RBD1Q).
- Urine sample for Dipstick urinalysis.
- Collect blood samples for:
  - DSA (Donor specific antibodies)- Order as HLA typing.
  - Peripheral markers: Neurotransmitters, Oxidative stress, cytokines, chemokines, and growth factors.
  - α-synuclein oligomers levels.
- Collect urine sample for α-synuclein oligomers
- Assess the Need for change in symptomatic therapy.

10.2 Telephone or email evaluations

These will occur at week 1, week 4, week 13, week 19, week 23, week 31, week 38, week 44, week 55, and week 75. In order to maintain continuous monitoring of the presence of AEs after the infusion visit, patients would either receive a phone call or an email to monitor signs or symptoms of adverse reactions to the transfusion, at regular intervals until the study end, with a total of 10 evaluation calls/emails (*See appendix O for a copy of the Telephone evaluation form*).

10.3 Study Timeline

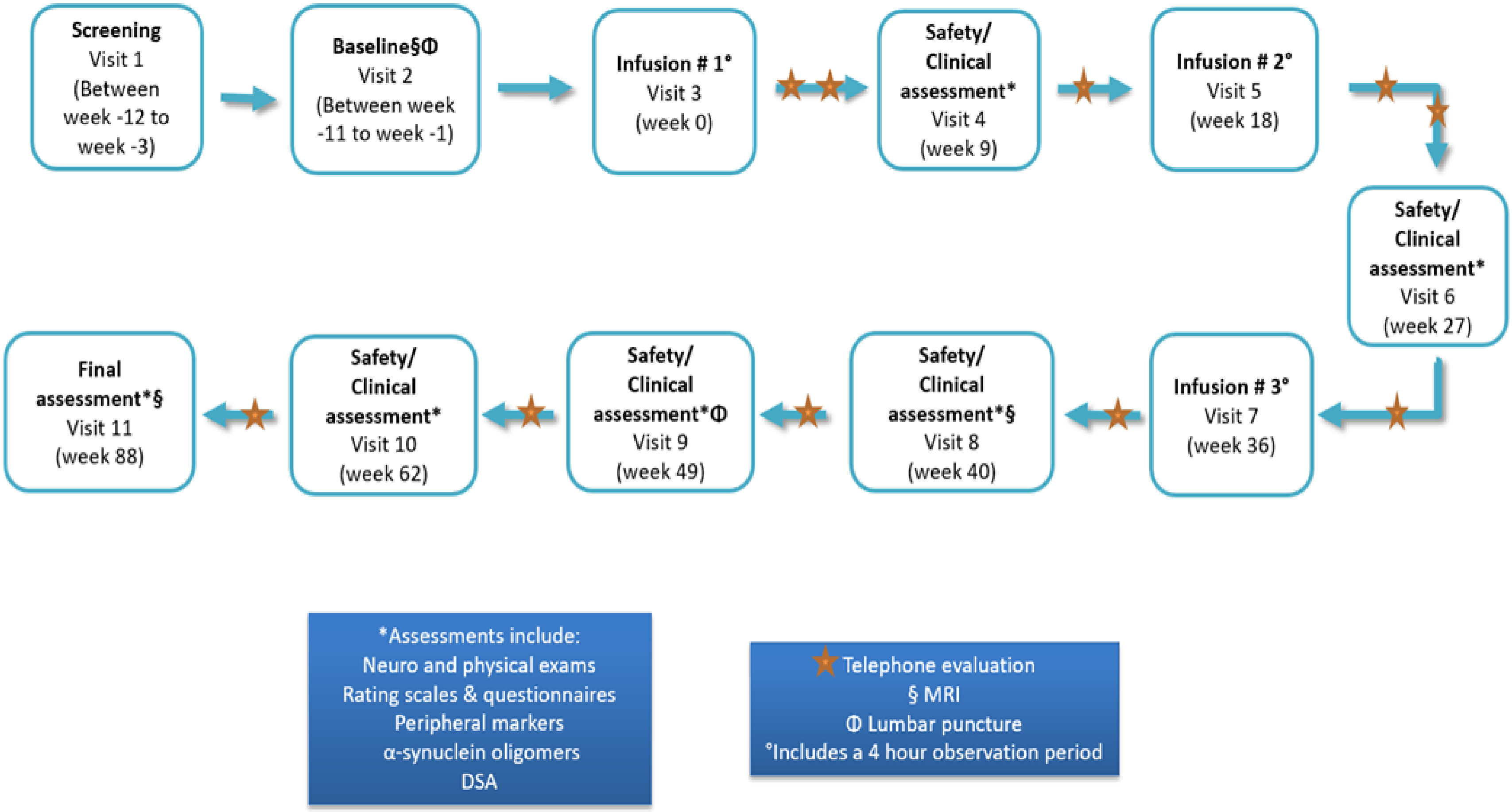

10.3 Study Schedule

				Treatment Period					Follow up Period					
		Screen visit	BL	INF 1	Safety/ Clinical	INF 2	Safety/ Clinical	INF 3	Safety/ Clinical	Safety/ Clinical	Safety/ Clinical	Final visit	Un-scheduled visit	Early termination visit
	**VISIT #**	***1***	***2***	***3***	***4***	***5***	***6***	***7***	***8***	***9***	***10***	***11***		
	**WEEKS**	Week -12 to week -3	Week -11 to week -1	0	Week 9 +/- 1.5 week	Week 18 +/- 1 week	Week 27 +/- 1.5 week	Week 36 +/- 1 week	Week 40 +/- 1.5 week	Week 49 +/- 1.5 week	Week 62 +/- 1.5 week	Week 88 +/- 1.5 week		
Clinical Assessment	Demographics, EKG, Specialty labs*	X												
	Physical and neurological examination. Meds review	X		X	X	X	X	X	X	X	X	X	X	X
	Vital Signs	X	X	X	X	X	X	X	X	X	X	X	X	X
	UPSIT & MOCA	X								X		X		X
	MDS- UPDRS, TUG, H&Y (OFF)	X			X		X		X		X	X		X
	MDS-UPDRS On	X												
	Questionnaires: ADL, PAS, GDS, PDQ-39, C-SSRS, EQ-5D-5L, NMS	X			X		X		X		X	X		X
Laboratory	Lumbar puncture**		X							X				
	CBC with differential and CMP	X			X		X		X			X		X
	Dipstick UA	X										X		
	Blood*** for peripheral markers		X		X		X		X	X		X		X
	Plasma α-synuclein oligomers		X						X	X		X		X
	Donor Specific Antibodies		X		X		X		X			X		X
	Infusion procedure and 4 hours Observation			X		X		X						
	Neuroimaging		X						X			X		X

*Specialty labs include INR, PT, and PTT.
**CSF for CBC + diff, glucose, protein, Neurotransmitters, Oxidative Stress Markers, α-synuclein oligomers, Cytokines, Chemokines, and Growth Factors.
*** Serum for Neurotransmitters, Oxidative Stress Markers, Cytokines, Chemokines, and Growth Factors.

11. Potential adverse events

11.1 Infusion-Related Allergic Reactions

The investigators will ensure that appropriate best clinical practices and treatment are initiated should an event indicating an allergic or hypersensitive reaction occur. Each event will be recorded as an AE or SAE, depending on which criteria are met by the event. The start and stop/restart times must be recorded in the CRF.

Should an infusion-related allergic reaction occur the infusion will be stopped immediately, and the subject closely monitored until stable. An infusion-related allergic reaction may appear as flushing, sudden rash, or shortness of breath or difficulty breathing.

Definitions and grading will follow the current NCI-CTCAE criteria. The current definitions and suggested treatment plans for an infusion-related allergic reaction are listed below:

Grade 1:

Mild transient reaction

1. Stop infusion and evaluate for severity. If reaction remains a grade 1, then restart at a reduced rate.
2. Reduce infusion rate by 50%,
3. Treat subject per good clinical practice (suggest antihistamines, corticosteroids, etc. as medically indicated) and monitor for worsening condition.
4. If the reaction persists or worsens, the infusion will be discontinued.

Grade 2:

Readily responds to clinical treatment (e.g., antihistamines, corticosteroids); should consider prophylactic treatment for ≤24 hours.

1. Pause infusion for up to 2 hours (product should not be infused after 4hours).
2. Administer clinical treatment for the allergic reaction as medically indicated.
3. Resume infusion at 50% of the previous rate once the reaction has decreased to Grade 1 in severity. Monitor closely for any worsening.
4. If the reaction reoccurs, stop the infusion. Study treatment is to be discontinued.

Grade 3:

Prolonged or severe reaction (e.g., not rapidly responsive to treatment or a recurrence of reaction after an initial improvement of a Grade 1 or Grade 2 reaction).

1. Discontinue infusion immediately. Study treatment is to be discontinued
2. Administer clinical treatment for the allergic reaction as medically indicated.
3. Report as an SAE

Grade 4:

Characterized as life-threatening AE; urgent intervention indicated to maintain hemostasis.

1. Discontinue infusion immediately. Study treatment will be discontinued.
2. Administer clinical treatment for the allergic reaction as medically indicated.
3. Report as an SAE

11.1.2 Algorithm for response in case of an allergic reaction.

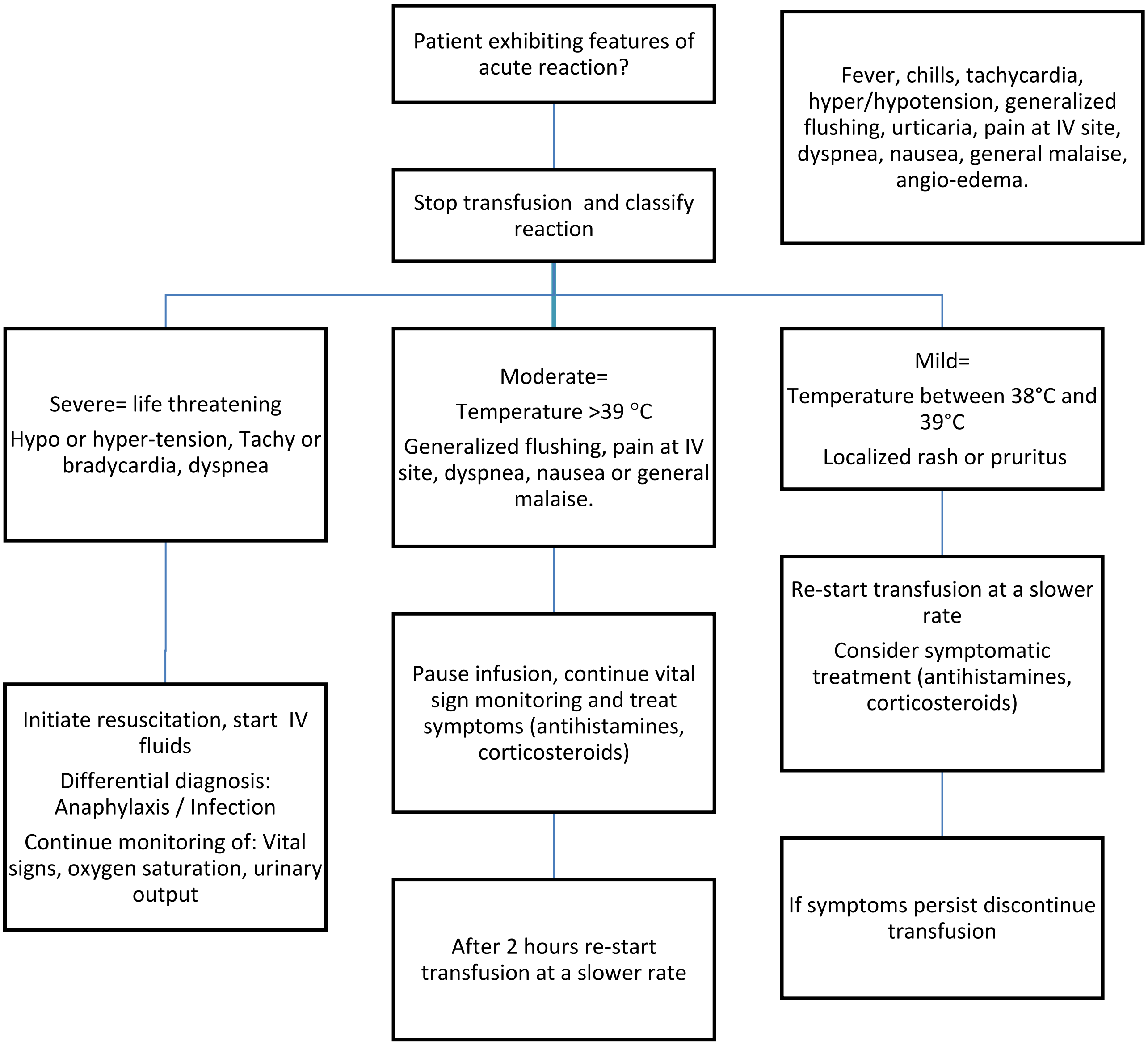

11.2 Sensitization to Donor Specific Antibodies (DSA).

There is a possibility of anti-donor alloimmune response after the first or second infusion of MSC, which might lead to a subsequent antibody-mediated rejection (AMR) with the following infusion. IF DSA are identified at either week 8 or 21 patients would be put on hold of their regular schedule on infusions and the PI will follow the algorithm describes in 11.2.1.

11.2.1 Algorithm for DSA response

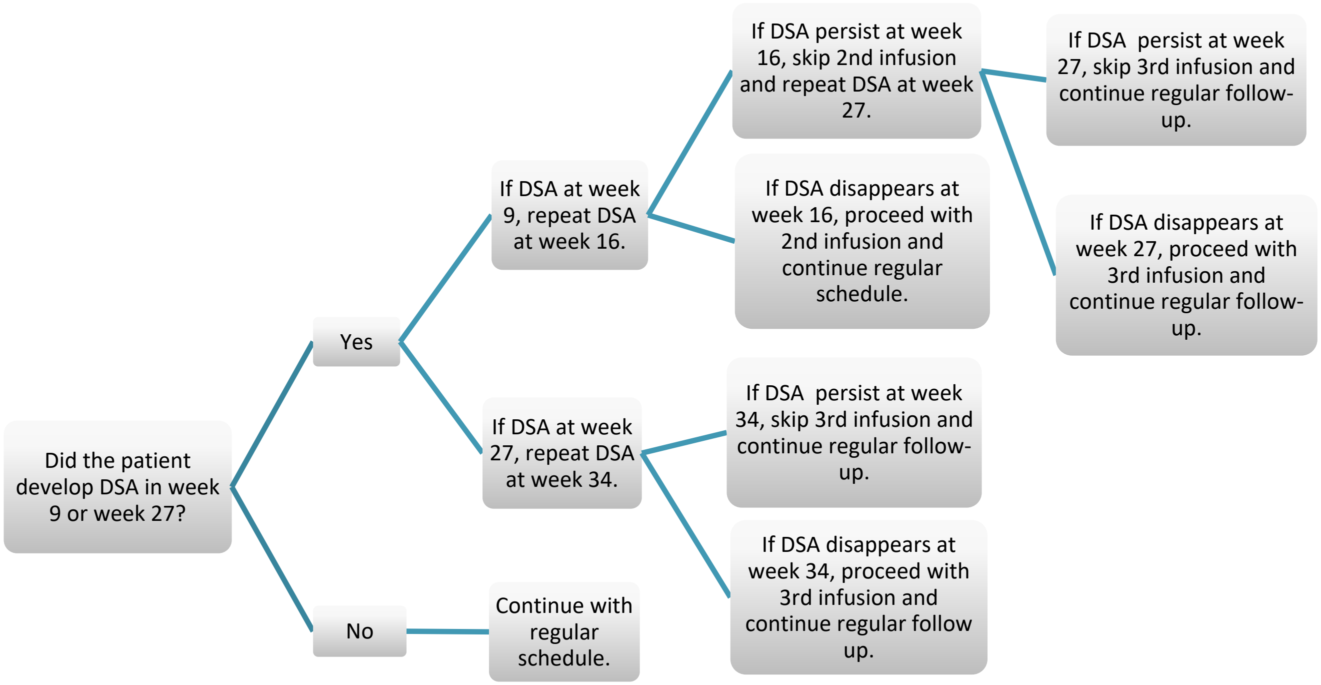

12. Management of adverse events

12.1 Adverse Events definitions

An Adverse Event (AE) will be defined as any unfavorable and unintended sign, symptom, or disease that occurs during the study, or is present at baseline and significantly worsens following the start of MSCs Infusion or any study procedure. Any AE that occurs prior to the first MSCs infusion will be considered part of the subject's medical history. AEs may include abnormal laboratory findings or other abnormal assessments (e.g., laboratory screening, vital signs, etc.). Accidental traumas are not considered SAEs but the medical condition resulting from the trauma may be an AE or SAE (e.g., a fracture resulting from a fall).

12.2 Serious Adverse Events (SAEs)

All serious adverse events (SAE) will be reported to the PI or the study coordinator. An SAE is any untoward medical occurrence that:

a. Results in death.
b. Is life-threatening: referring to an event in which the subject was at risk of death at the time of the event. It does not refer to an event, which hypothetically might have caused death if it were more severe or left untreated.
c. Requires hospitalization or prolongation of existing hospitalization. NOTE: Hospitalization describes an admission for > 24 hours. Over-night stays for observation or stays at the emergency room do not constitute hospitalization. However, the medical judgment must always be exercised and, when in doubt, should be considered an SAE. Any medical/surgical complications which prolong the hospitalization are SAEs. Hospitalization for elective treatment or for a pre-existing condition that did not worsen from baseline is not considered an AE.
d. Results in disability/incapacity. NOTE: The Investigator must determine that the disability is not related to the expected progression of the patient Parkinson's disease. Also, the term disability means a substantial disruption of a person's ability to conduct normal life functions. This definition is not intended to include experiences of relatively minor medical significance such as uncomplicated headache, nausea, vomiting, diarrhea, and infections which may temporarily interfere or prevent everyday life functions but do not constitute a substantial disruption.
e. AEs that occur in a higher frequency than can be expected in the PD population.
f. Any event that the Primary Investigator judges to be an SAE.

12.3 AE Relationship

- Definitely related: symptoms experienced a temporal sequence from MSC administration or discontinuance.
- Probably related: symptoms experienced a temporal sequence from MSC administration or discontinuance and cannot be reasonably explained by another known clinical condition.
- Possibly related: symptoms experienced a temporal sequence from MSC administration or discontinuance but could have been produced by another known clinical condition or by other therapies administered to the subject.
- Unlikely related: The temporal sequence between the AE/SAE and the MSC administration is such that the infusion is not likely to have had any reasonable association with the symptoms, and the AE/SAE could have been produced by another known clinical condition or by other therapies administered to the subject.
- Unrelated: The AE/SAE is definitely produced by another known clinical condition or by other therapies administered to the subject, and the AE does not follow a temporal sequence from MSC administration or discontinuance.

12.4 AE/SAE Reporting

AE/SAE will be documented at each clinic visit starting at visit 3. Information on adverse effects will be determined at each clinic visit by direct questioning of the subjects, vital signs, clinical assessment, laboratory test, and by interval telephone evaluations starting 1 week after the first infusion using a set of questions to identify symptoms or signs related to an adverse event and will continue through the rest of the study.

The PI, with the assistance of her study team, will be responsible for the detection and documentation of events meeting the criteria and definition of an AE or SAE as provided in this protocol. For each AE and SAE, the PI will make an assessment of:

1. The severity grade (CTCAE criteria).
2. The duration (start and end dates or if continuing at the Safety Follow-up Visit).
3. Its relationship to the study treatment (Reasonable possibility that AE is related: No, Yes). The investigator will use clinical judgment to determine the relationship, alternative causes (e.g. the natural history of an underlying disease, concomitant therapy, other risk factors), and the temporal relationship of the AE to the infusion procedure will be considered and investigated.
4. Action taken with respect to study or investigational treatment (none, dose adjusted, temporarily interrupted, permanently discontinued, hospitalized, unknown, not applicable).
5. Whether medication or therapy was given (no concomitant medication/non-drug therapy, concomitant medication/non-drug therapy).

6. Outcome (not recovered/not resolved, recovered/resolved, recovering/resolving, recovered/resolved with sequelae, fatal, unknown).
7. Whether it is serious, where a serious adverse event (SAE) is defined as in Section 12.2.

All Adverse events that meet all three definitions described above of a Suspected (related or possibly related to study drug or participation in the research), Unexpected and Serious Adverse Reaction (SUSAR) must be reported as soon as possible but no later than 7 calendar days after the investigator's initial receipt of the information, to the University of Texas Health Science IRB and the FDA. If the SUSAR report involves the death of a participant, the event needs to be reported to IRB/FDA within 24 hours after first knowledge by the investigator via facsimile or email or by telephone. Annually a list of all AEs & SAEs will be complied in an approved format and will be submitted to the FDA as part of the IND Annual FDA Report.

12.5 Follow-up AEs and SAEs

All AEs and SAEs documented at a previous visit and designated as on-going, will be reviewed at subsequent visits until resolved or at the time of the subject's end of study participation. All SAEs that the primary investigator determines is related to the study will be followed until resolution, until the condition stabilizes, until the event is otherwise explained, until the subject is lost to follow-up or until the patient has completed their study follow up period. Once resolved, the appropriate AE/SAE forms page(s) will be updated in the CRF and the IRB will be updated as appropriate.

13. Study Modification/Discontinuation

The study may be modified or discontinued at any time by the IRB, the DSMB or the FDA as part of their duties to ensure that research subjects are protected

13.1 Subject Withdrawal or Termination

Subjects' participation is considered complete after the final visit at 78 weeks. However, subjects may withdraw from the study at any time for any reason, without any consequence.

In addition, a subject may be withdrawn from the study by the investigator for reasons including the following:

1. Lost to follow-up
2. Adverse Event (AE) or Serious Adverse Event (SAE)
3. Patient choice (withdrawal of consent; the investigator will attempt to ascertain reason)
4. Protocol violation/non-compliance

5. Investigator's decision to terminate the study
6. Development of a new co-morbid neurological, medical or psychiatric disorder that interferes with the measurement of disease progression.

Subjects are free to withdraw at any time. The primary reason for withdrawals from the study will be recorded in the study exit form. Subjects who withdraw or are withdrawn will be encouraged to complete an Early Termination Visit. Although subjects are free to withdraw at any time, subjects will be encouraged to remain in the study for follow- up evaluations. Subjects who withdraw will be discontinued from all study procedures and will be encouraged to return to their primary neurologist for follow-up care.

13.2 Lost to Follow-up

If a subject fails to appear for any visit, three attempts on separate occasions will be made by telephone and/or e-mail to contact the subjects, if there is no response this should be followed by a certified letter to the last known address. The contact attempts will be recorded on the study exit form. Every effort will be made to determine the subject's health status and document the subject outcome if possible.

13.3 Screen Failures

Screen Failures are defined as any patient who has been consented but fails to continue to meet inclusion/exclusion criteria prior to the MSCs infusion. Any patient that discontinues the study subsequent to the infusion will be a study patient withdrawal.

14. Statistical Considerations

Bayesian Statistical Methods. Developing effective neurological interventions requires incremental improvement of theoretically sound treatments based on systematically accruing data. Indeed, addressing the so-called "Pipeline Problem" in developing clinical applications, the FDA has indicated that Bayesian statistics offers one avenue for improved methodological efficiency[152]. Decision-making based on an initial treatment trial is assisted by estimates of the probability of an effect of some specified magnitude. These statements, not part of the conventional, Frequentist statistical lexicon, are accessible via Bayesian approaches, particularly with small sample sizes.

14.1 Blinding and Randomization

This is a double-blind placebo-controlled study. Subjects and site personnel will be blinded to the randomization assignment except for one study person who will be authorized to randomize and keep the blind (or break the blind if needed). Subjects will be randomized to receive any of the 3 treatment arms in 1:1:1 ratio using a computer table randomization method by the statistician prior to the blinded study treatment.

14.2 Sample Size:

Having demonstrated the safety of MSC Therapy in a previous study, sample size justification will focus on Hypothesis 1.1. Schulman et al[153] define small, medium, and large clinically important differences on the UPDRS- Motor-as 2.5, 5.2, and 10.8 points respectively. These correspond to Cohen's d = 0.19, 0.39 and 0.81 respectively. Current estimates assume N = 45 participants randomized in 1:1:1 fashion to three conditions and a medium effect size (M-UPDRS difference = 5.2). We stipulate that relative to placebo if any treatment has a posterior probability > 0.70 of conferring improvement (MDS-UPDRS-M difference > 0) and a median posterior estimate of the effect size at least as large as an MDS-UPDRS-M difference of 2.5 (i.e. a small effect), that this treatment is eligible for continued investigation in a larger clinical trial. Under these assumptions, M = 1000 Monte Carlo simulations using the normal approximation to the posterior distribution indicate that the proposed design will identify an effect of treatment 72% of the time.

14.3 Statistical analysis

Hypothesis 1.1. At least one active MSC group will demonstrate improved scores (≥ -5) relative to placebo at 62 weeks follow-up. Generalized linear modeling will evaluate the MDS-UPDRS motor section as a function of treatment. Application of the decision-rules, discussed in the Sample Size Section, to the resulting posteriors will identify the candidate doses that warrant further investigation.

Hypothesis 2.1. Generalized linear modeling will characterize serious adverse reactions, significant worsening of general and motor functional state, activities of daily living, and peripheral immunologic responses. In each case, the subtraction of the posterior distribution for the treatment condition from the posterior distribution of the placebo condition will permit estimates of the posterior probability that the active condition is better than or equal to the control.

Hypothesis 2.2. Generalized linear modeling will estimate the improvement in motor function, disability, quality of life, non-motor symptoms, cognitive and behavioral symptoms as a function of treatment.

Hypothesis 3.1. Bayesian, multilevel structural equation modeling will evaluate the degree to which cytokines, chemokines, growth factors, and neurotransmitters transmit the effect of treatment to the MDS-UPDRS motor section at 62 weeks.

Hypothesis 3.2. Separate multilevel generalized linear modeling to account for correlations due to repeated observations within participants will evaluate the MDS-UPDRS motor section as a function of time and time-varying covariates, including kinetic parameters of αSyn-PMCA in plasma and CSF before and after treatment.

14.4 Interim analyses

At Week 29 will mirror those at trial completion, but only for those outcomes on which data has been collected at that point:

Hypothesis 1.1. At least one active MSC group will demonstrate improved scores (≥ -5) relative to placebo at 40 weeks follow up. Generalized linear modeling will evaluate the motor MDS-UPDRS as a function of treatment.

Hypothesis 2.1. Generalized linear modeling will characterize severe adverse reactions, significant worsening of general and motor functional state, activities of daily living, and peripheral immunologic responses. In each case, the subtraction of the posterior distribution for the treatment condition from the posterior distribution of the placebo condition will permit estimates of the posterior probability that the active condition is better than or equal to the control.

Hypothesis 2.2. Generalized linear modeling will estimate the improvement in motor function, disability, quality of life, non-motor symptoms, cognitive and behavioral symptoms as a function of treatment.

Hypothesis 3.1. Bayesian, multilevel structural equation modeling will evaluate the degree to which cytokines, chemokines, growth factors, and neurotransmitters transmit the effect of treatment to the MDS-UPDRS at 40 weeks.

Hypothesis 3.2. Separate multilevel generalized linear modeling to account for correlations due to repeated observations within participants will evaluate MDS-UPDRS Motor as a function of time and time-varying covariates, including kinetic parameters of αSyn-PMCA in plasma and CSF before and after treatment.

While multiple analyses often require correction for Type I Error, the Bayesian approach to analysis, because it obeys the likelihood principle, does not require this. Moreover, given the

degree of uncertainty that will exist in the form of the posterior distribution variances, the primary concern will be with brining the trial to a premature conclusion for lack of benefit, when in fact, such benefit does exist (Type II Error).

15. Data collection and management

15.1 Records to Be Kept

The master trial file will include but not be limited to source documents, monitoring visit logs, regulatory documents, investigator and coordinators CVs and appropriate certifications, and correspondence pertaining to the trial. Source data is defined as all information in original records of clinical findings, observations, copies or other activities occurring in a clinical trial. These source data are necessary for the data quality of the clinical trial.

15.2 Data Management

The Principal investigator will complete an enrollment log to permit the identification of each subject and will file the master log in the study file. Additionally, the research coordinator will create a chart for each subject in which all CRF data will be recorded. The Chart will be protected with HIPAA compliant techniques. The research scientist will create a database in REDCap that meets all verification and validation requirements of FDA rule 21 CFR 11, including reference to all pertinent FDA-provided guidelines. The study will use a REDcap database to store the de-identified information of all the visits for each subject.

Source Data and Documentation

Source documentation will be available to confirm data collected in the CRF and for resolution of SAE/AEs. A photocopy of the Label (s) on the Cell bag must be retained in the Coordinators source document binder but the bag may be discarded per hospital standard for blood products. Additionally, a chart of copied progress notes, vital sign records, imaging and lab result from the observation period must be retained in the study documents to verify presence or absence of AEs. A common term for this chart is “Shadow Chart” since it shadows the actual patient chart. The Shadow Chart must be protected with the same HIPAA compliant techniques as actual charts are protected. Data from any subsequent admission need only be retained as it applies to AEs.

CAGT Documentation

Records are kept of all of the items that are cited. These include:

- Donor identity
- Donor infectious disease testing as indicated in the CMC section
- Risk behavior questionnaire
- Health evaluation for donation
- Former donor eligibility declaration
- Records of all cell culture procedures
- Results of all in-process and release tests as described in the CMC section

Cells are stored according to Standard Operating Procedures using a formal inventory system that includes the sample identity, date of cryopreservation, sample location, donor identification, and passage number.

Data Quality Assurance

The Study PI or designee will conduct ongoing subject data quality assurance audits. The audit may include, but will not be limited to, a review of all subject ICFs, a review of CRFs and source documents, a review of regulatory documents, an assessment of trial conduct, and compliance. Data quality will be maintained by establishing a written Data Management Plan describing all applicable aspects of the data management process including: Developing a database that meets all verification and validation requirements of FDA rule 21 CFR 11, including reference to all pertinent FDA-provided guidelines.

16. Data safety monitoring board (DSMB)

An independent DSMB will monitor the conduct of the trial and subject safety based on the review of the aggregate data one-month post each infusion. The DSMB will review the aggregate data after each infusion of the initial 22 patients and determine the safety to proceed to the next infusion. The DSMB will review the aggregate data for all the patients in order to determine safety regarding neurological and physical status. At any review, the DSMB may recommend the study continues as per protocol or recommend changes to the protocol or the conduct of the study. The DSMB can recommend suspension or study termination at any time for any reason.

The DSMB Plan will be formulated and agreed upon by the Primary Investigator and DSMB Board. The DSMB will be notified within 24 hours of an SAE. All or the majority of the DSMB members will meet to discuss the SAE report promptly.

Mandatory Unscheduled DSMB Review:

Study suspension is defined as a hold on recruitment. The following criteria require a review and a recommendation from the DSMB for continuation, suspension or termination of the study:

- Unexpected significant worsening of functional motor state as documented by a change at 6 months in: the MDS-UPDRS total score of > 10 points or > 5 points for the motor MDS-UPDRS subscale or an increase in H&Y of 1 or more.
- An SAE related or possibly-related to the infusion, occurring at any time post-dosing
- 2 possibly-related or related, unexpected, Grade 3 or higher Infusion-Related Allergic Reactions observed per 3 treated subjects at any time post-infusion.

17. Intellectual Property and data sharing:

As a naturally occurring unmodified cell that exists in the bone marrow, our proposed mesenchymal stem cells cannot be patented (Leahy–Smith America Invents Act (AIA), Pub. L. 112-29, sec. 33(a), 125 Stat. 284). The MSC manufactured rights belong to the Center for Cell and Gene Therapy at Baylor College of Medicine. All the biospecimens, neuro-imaging, and measurements collected for this study are considered the intellectual property of the University of Texas Health Science Center at Houston.

The results of this study will be published in a scientific journal and will be presented at national and international scientific meetings. A summary of the results will be available in clinical trial.gov, Research Match, and Fox Trial Finder website. Additionally, we will send a newsletter to our patients at the end of the study.

18. Ethics

This protocol will be submitted and review by the local Institutional Review Board:

The Committee for the Protection of Human Subjects
The University of Texas Health Science Center at Houston
6410 Fannin Street, Suite 1100
Houston, Texas 77030
Institutional Official: Anne Dougherty, MD FWA# 00000667
IRB Registration Numbers: IRB00000308, IRB00003763, IRB00004604, IRB00008445

In addition, the study will be conducted according to the standards that meet Good Clinical Practice (GCP). These standards respect the following guidelines:

1. Guideline for Good Clinical Practice (International Conference on Harmonization ICH of Technical Requirements for the Registration of Pharmaceuticals for Human Use).
2. United States (US) Code of Federal Regulations (CFR) dealing with clinical studies (21 CFR

Parts 50, 54, 56, 312, 314, and 511).

3. Declaration of Helsinki, concerning medical research in humans ("Recommendations Guiding Physicians in Biomedical Research Involving Human Subjects,") Helsinki 1964, amended Tokyo 1975, Venice 1983, Hong Kong 1989 and revised version of Somerset West, Republic of South Africa, October, 1996, Note of Clarification added by the WMA World Medical Association General Assembly, Washington 2002, Note of Clarification added by the WMA General Assembly, Tokyo 2004

19. Consent

The consent form will describe the purpose of the study, the procedures to be followed, and the risks and benefits of participation. Potential subjects are to be fully explained the protocol and study procedures and allowed them to ask any questions. All subjects must sign the IRB approved informed consent form before performing any study-related activity (including screening activities). For subjects who cannot consent for themselves the legally authorized representative (LAR) must sign the consent form. The original signed consent form is to be placed in the subject's study file. A copy of the signed consent form is to be given to subjects or LAR for their records.

Prior to obtaining written informed consent, subjects will be asked to be in the OFF- medication state (i.e. hold PD medications) for at least 8-12 hours before coming to the screening visit appointment. Subjects will be asked to bring their PD medications to the CRU. After performing an OFF-state neurological exam (Modified H&Y and MDS-UPDRS), subjects will be asked to take their PD medications with a snack, wait for an hour, and then the motor section of the MDS-UPDRS) will be repeated in the ON-medicine state to assess improvements in motor symptoms. Subjects will be emailed written instructions on how to prepare for the screening visit and their response will be considered as their willingness and verbal consent to preparation.

20. Contacts

For MSC related issues	Zhuyong Mei, PhD: 832-232-2283
For Study-related issues	Mya Schiess, MD: 713-500-7121<br>Jessika Suescun, MD: 713-500-7074<br>Vanessa Thyne, MS: 713-500-7051
For UTHealth Clinical Research Unit (CRU) at Memorial Hermann Hospital (MHH) - Texas Medical Center (TMC)	Kathy Franco, RN: 713-704-4147<br>CRU Unit: 713-704-4137
The University of Texas Health Science Center Committee for the Protection of Human Subjects 713-500-7942.	

21. References

1. Dorsey ER, Sherer T, Okun MS, Bloem BR. The Emerging Evidence of the Parkinson Pandemic. *J Parkinsons Dis.* 2018;8(s1):S3-S8.
2. Braak H, Del Tredici K, Rub U, de Vos RA, Jansen Steur EN, Braak E. Staging of brain pathology related to sporadic Parkinson's disease. *Neurobiol Aging.* 2003;24(2):197-211.
3. Connolly BS, Lang AE. Pharmacological treatment of Parkinson disease: a review. *JAMA.* 2014;311(16):1670-1683.
4. Ossig C, Reichmann H. Treatment strategies in early and advanced Parkinson disease. *Neurol Clin.* 2015;33(1):19-37.
5. Jellinger KA. Basic mechanisms of neurodegeneration: a critical update. *Journal of cellular and molecular medicine.* 2010;14(3):457-487.
6. Kortekaas R, Leenders KL, van Oostrom JC, et al. Blood-brain barrier dysfunction in parkinsonian midbrain in vivo. *Ann Neurol.* 2005;57(2):176-179.
7. Al-Bachari S, Vidyasagar R, Emsley H, Parkes L. PO071 Mri assessment of neurovascular changes in idiopathic parkinson’s disease. *Journal of Neurology, Neurosurgery & Psychiatry.* 2017;88(Suppl 1):A30-A30.
8. Gray MT, Woulfe JM. Striatal blood-brain barrier permeability in Parkinson's disease. *Journal of cerebral blood flow and metabolism : official journal of the International Society of Cerebral Blood Flow and Metabolism.* 2015;35(5):747-750.
9. McGeer PL, Itagaki S, Akiyama H, McGeer EG. Rate of cell death in parkinsonism indicates active neuropathological process. *Ann Neurol.* 1988;24(4):574-576.
10. Nagatsu T, Mogi M, Ichinose H, Togari A. Cytokines in Parkinson's disease. *J Neural Transm Suppl.* 2000(58):143-151.
11. Orr CF, Rowe DB, Halliday GM. An inflammatory review of Parkinson’s disease. *Progress in Neurobiology.* 2002;68(5):325-340.

12. Gerhard A, Pavese N, Hotton G, et al. In vivo imaging of microglial activation with [11C](R)-PK11195 PET in idiopathic Parkinson's disease. *Neurobiol Dis.* 2006;21(2):404-412.
13. Mogi M, Harada M, Riederer P, Narabayashi H, Fujita K, Nagatsu T. Tumor necrosis factor-alpha (TNF-alpha) increases both in the brain and in the cerebrospinal fluid from parkinsonian patients. *Neuroscience letters.* 1994;165(1-2):208-210.
14. Stypula G, Kunert-Radek J, Stepien H, Zylinska K, Pawlikowski M. Evaluation of interleukins, ACTH, cortisol and prolactin concentrations in the blood of patients with parkinson's disease. *Neuroimmunomodulation.* 1996;3(2-3):131-134.
15. Bas J, Calopa M, Mestre M, et al. Lymphocyte populations in Parkinson's disease and in rat models of parkinsonism. *Journal of neuroimmunology.* 2001;113(1):146-152.
16. Brochard V, Combadiere B, Prigent A, et al. Infiltration of CD4+ lymphocytes into the brain contributes to neurodegeneration in a mouse model of Parkinson disease. *J Clin Invest.* 2009;119(1):182-192.
17. Kempuraj D, Thangavel R, Natteru P, et al. Neuroinflammation Induces Neurodegeneration. *J Neurol Neurosurg Spine.* 2016;1(1).
18. Benner EJ, Banerjee R, Reynolds AD, et al. Nitrated alpha-synuclein immunity accelerates degeneration of nigral dopaminergic neurons. *PLoS One.* 2008;3(1):e1376.
19. Lee JK, Tran T, Tansey MG. Neuroinflammation in Parkinson's disease. *Journal of neuroimmune pharmacology : the official journal of the Society on NeuroImmune Pharmacology.* 2009;4(4):419-429.
20. Hirsch EC, Hunot S. Neuroinflammation in Parkinson's disease: a target for neuroprotection? *Lancet Neurol.* 2009;8(4):382-397.
21. Chen H, Jacobs E, Schwarzschild MA, et al. Nonsteroidal antiinflammatory drug use and the risk for Parkinson's disease. *Ann Neurol.* 2005;58(6):963-967.
22. Gagne JJ, Power MC. Anti-inflammatory drugs and risk of Parkinson disease: a meta-analysis. *Neurology.* 2010;74(12):995-1002.
23. Schiess M. Nonsteroidal anti-inflammatory drugs protect against Parkinson neurodegeneration: can an NSAID a day keep Parkinson disease away? *Arch Neurol.* 2003;60(8):1043-1044.
24. Doursout M-F, Schurdell MS, Young LM, et al. Inflammatory cells and cytokines in the olfactory bulb of a rat model of neuroinflammation; insights into neurodegeneration? *Journal of interferon & cytokine research : the official journal of the International Society for Interferon and Cytokine Research.* 2013;33(7):376-383.
25. Doursout M-F, Liang Y, Schiess MC, et al. Are Temporal Differences in GDNF and NOS Isoform Induction Contributors to Neurodegeneration? A Fluorescence Microscopy-Based Study. *The open neurology journal.* 2016;10:67-76.
26. Bick RJ, Poindexter BJ, Kott MM, et al. Cytokines disrupt intracellular patterns of Parkinson's disease-associated proteins alpha-synuclein, tau and ubiquitin in cultured glial cells. *Brain research.* 2008;1217:203-212.
27. Schiess MC, Barnes JL, Ellmore TM, Poindexter BJ, Dinh K, Bick RJ. CSF from Parkinson disease patients differentially affects cultured microglia and astrocytes. *BMC neuroscience.* 2010;11:151-151.
28. Wang Y, Shi M, Chung KA, et al. Phosphorylated α-synuclein in Parkinson's disease. *Science translational medicine.* 2012;4(121):121ra120-121ra120.
29. Smith LM, Schiess MC, Coffey MP, Klaver AC, Loeffler DA. alpha-Synuclein and anti-alpha-synuclein antibodies in Parkinson's disease, atypical Parkinson syndromes, REM sleep behavior disorder, and healthy controls. *PLoS One.* 2012;7(12):e52285.

30. Csencsits-Smith K, Suescun J, Li K, Luo S, Bick DL, Schiess M. Serum Lymphocyte-Associated Cytokine Concentrations Change More Rapidly over Time in Multiple System Atrophy Compared to Parkinson Disease. *Neuroimmunomodulation.* 2017.
31. Friedenstein AJ, Petrakova KV, Kurolesova AI, Frolova GP. Heterotopic of bone marrow. Analysis of precursor cells for osteogenic and hematopoietic tissues. *Transplantation.* 1968;6(2):230-247.
32. Glavaski-Joksimovic A, Bohn MC. Mesenchymal stem cells and neuroregeneration in Parkinson's disease. *Experimental neurology.* 2013;247:25-38.
33. Aleynik A, Gernavage KM, Mourad Y, et al. Stem cell delivery of therapies for brain disorders. *Clin Transl Med.* 2014;3:24.
34. Momin EN, Mohyeldin A, Zaidi HA, Vela G, Quinones-Hinojosa A. Mesenchymal stem cells: new approaches for the treatment of neurological diseases. *Curr Stem Cell Res Ther.* 2010;5(4):326-344.
35. Parkinson Study Group S-PDI, Schwarzschild MA, Ascherio A, et al. Inosine to increase serum and cerebrospinal fluid urate in Parkinson disease: a randomized clinical trial. *JAMA Neurol.* 2014;71(2):141-150.
36. Simuni T, Borushko E, Avram MJ, et al. Tolerability of isradipine in early Parkinson's disease: a pilot dose escalation study. *Mov Disord.* 2010;25(16):2863-2866.
37. Remy P. Biotherapies for Parkinson disease. *Rev Neurol (Paris).* 2014;170(12):763-769.
38. Tanna T, Sachan V. Mesenchymal stem cells: potential in treatment of neurodegenerative diseases. *Curr Stem Cell Res Ther.* 2014;9(6):513-521.
39. Joyce N, Annett G, Wirthlin L, Olson S, Bauer G, Nolta JA. Mesenchymal stem cells for the treatment of neurodegenerative disease. *Regen Med.* 2010;5(6):933-946.
40. Olanow CW, Goetz CG, Kordower JH, et al. A double-blind controlled trial of bilateral fetal nigral transplantation in Parkinson's disease. *Ann Neurol.* 2003;54(3):403-414.
41. Freed CR, Greene PE, Breeze RE, et al. Transplantation of embryonic dopamine neurons for severe Parkinson's disease. *N Engl J Med.* 2001;344(10):710-719.
42. Kefalopoulou Z, Politis M, Piccini P, et al. Long-term clinical outcome of fetal cell transplantation for Parkinson disease: two case reports. *JAMA Neurol.* 2014;71(1):83-87.
43. Capitelli CS, Lopes CS, Alves AC, et al. Opposite effects of bone marrow-derived cells transplantation in MPTP-rat model of Parkinson's disease: a comparison study of mononuclear and mesenchymal stem cells. *Int J Med Sci.* 2014;11(10):1049-1064.
44. Jiaming M, Niu C. Comparing neuroprotective effects of CDNF-expressing bone marrow derived mesenchymal stem cells via differing routes of administration utilizing an in vivo model of Parkinson's disease. *Neurological sciences : official journal of the Italian Neurological Society and of the Italian Society of Clinical Neurophysiology.* 2015;36(2):281-287.
45. Park HJ, Bang G, Lee BR, Kim HO, Lee PH. Neuroprotective effect of human mesenchymal stem cells in an animal model of double toxin-induced multiple system atrophy parkinsonism. *Cell Transplant.* 2011;20(6):827-835.
46. Park HJ, Shin JY, Kim HN, Oh SH, Lee PH. Neuroprotective effects of mesenchymal stem cells through autophagy modulation in a parkinsonian model. *Neurobiol Aging.* 2014;35(8):1920-1928.
47. Suzuki S, Kawamata J, Iwahara N, et al. Intravenous mesenchymal stem cell administration exhibits therapeutic effects against 6-hydroxydopamine-induced dopaminergic neurodegeneration and glial activation in rats. *Neuroscience letters.* 2015;584:276-281.
48. Wang F, Yasuhara T, Shingo T, et al. Intravenous administration of mesenchymal stem cells exerts therapeutic effects on parkinsonian model of rats: focusing on neuroprotective effects of stromal cell-derived factor-1alpha. *BMC Neurosci.* 2010;11:52.

49. Schwerk A, Altschuler J, Roch M, et al. Human adipose-derived mesenchymal stromal cells increase endogenous neurogenesis in the rat subventricular zone acutely after 6-hydroxydopamine lesioning. *Cytotherapy.* 2015;17(2):199-214.
50. Riecke J, Johns KM, Cai C, et al. A Meta-Analysis of Mesenchymal Stem Cells in Animal Models of Parkinson's Disease. *Stem Cells Dev.* 2015.
51. Gugliandolo A, Bramanti P, Mazzon E. Mesenchymal stem cell therapy in Parkinson's disease animal models. *Current research in translational medicine.* 2017;65(2):51-60.
52. Wang Y, Chen X, Cao W, Shi Y. Plasticity of mesenchymal stem cells in immunomodulation: pathological and therapeutic implications. *Nat Immunol.* 2014;15(11):1009-1016.
53. Kitada M, Dezawa M. Parkinson's disease and mesenchymal stem cells: potential for cell-based therapy. *Parkinsons Dis.* 2012;2012:873706.
54. Park HJ, Lee PH, Bang OY, Lee G, Ahn YH. Mesenchymal stem cells therapy exerts neuroprotection in a progressive animal model of Parkinson's disease. *J Neurochem.* 2008;107(1):141-151.
55. Glavaski-Joksimovic A, Virag T, Chang QA, et al. Reversal of dopaminergic degeneration in a parkinsonian rat following micrografting of human bone marrow-derived neural progenitors. *Cell Transplant.* 2009;18(7):801-814.
56. Bouchez G, Sensebe L, Vourc'h P, et al. Partial recovery of dopaminergic pathway after graft of adult mesenchymal stem cells in a rat model of Parkinson's disease. *Neurochemistry international.* 2008;52(7):1332-1342.
57. Shintani A, Nakao N, Kakishita K, Itakura T. Protection of dopamine neurons by bone marrow stromal cells. *Brain research.* 2007;1186:48-55.
58. Lai RC, Yeo RW, Lim SK. Mesenchymal stem cell exosomes. *Semin Cell Dev Biol.* 2015;40:82-88.
59. Kim HJ, Lee JH, Kim SH. Therapeutic effects of human mesenchymal stem cells on traumatic brain injury in rats: secretion of neurotrophic factors and inhibition of apoptosis. *J Neurotrauma.* 2010;27(1):131-138.
60. Savitz SI, Misra V, Kasam M, et al. Intravenous autologous bone marrow mononuclear cells for ischemic stroke. *Ann Neurol.* 2011;70(1):59-69.
61. Li Y, Chen J, Chen XG, et al. Human marrow stromal cell therapy for stroke in rat: neurotrophins and functional recovery. *Neurology.* 2002;59(4):514-523.
62. Parr AM, Tator CH, Keating A. Bone marrow-derived mesenchymal stromal cells for the repair of central nervous system injury. *Bone marrow transplantation.* 2007;40(7):609-619.
63. Pisati F, Bossolasco P, Meregalli M, et al. Induction of neurotrophin expression via human adult mesenchymal stem cells: implication for cell therapy in neurodegenerative diseases. *Cell Transplant.* 2007;16(1):41-55.
64. Li Y, Chopp M. Marrow stromal cell transplantation in stroke and traumatic brain injury. *Neuroscience letters.* 2009;456(3):120-123.
65. Baraniak PR, McDevitt TC. Stem cell paracrine actions and tissue regeneration. *Regen Med.* 2010;5(1):121-143.
66. Chen JL, Guo ZK, Xu C, et al. [Mesenchymal stem cells suppress allogeneic T cell responses by secretion of TGF-beta1]. *Zhongguo Shi Yan Xue Ye Xue Za Zhi.* 2002;10(4):285-288.
67. Crigler L, Robey RC, Asawachaicharn A, Gaupp D, Phinney DG. Human mesenchymal stem cell subpopulations express a variety of neuro-regulatory molecules and promote neuronal cell survival and neuritogenesis. *Experimental neurology.* 2006;198(1):54-64.
68. Croitoru-Lamoury J, Lamoury FM, Zaunders JJ, Veas LA, Brew BJ. Human mesenchymal stem cells constitutively express chemokines and chemokine receptors that can be upregulated by cytokines, IFN-beta, and Copaxone. *J Interferon Cytokine Res.* 2007;27(1):53-64.

UTHealth Houston IRB NUMBER: HSC-MS-20-0150
IRB APPROVAL DATE: 03/08/2024

69. Honczarenko M, Le Y, Swierkowski M, Ghiran I, Glodek AM, Silberstein LE. Human bone marrow stromal cells express a distinct set of biologically functional chemokine receptors. *Stem Cells.* 2006;24(4):1030-1041.
70. Tate CC, Fonck C, McGrogan M, Case CC. Human mesenchymal stromal cells and their derivative, SB623 cells, rescue neural cells via trophic support following in vitro ischemia. *Cell Transplant.* 2010;19(8):973-984.
71. Wakabayashi K, Nagai A, Sheikh AM, et al. Transplantation of human mesenchymal stem cells promotes functional improvement and increased expression of neurotrophic factors in a rat focal cerebral ischemia model. *J Neurosci Res.* 2010;88(5):1017-1025.
72. Lin LF, Doherty DH, Lile JD, Bektesh S, Collins F. GDNF: a glial cell line-derived neurotrophic factor for midbrain dopaminergic neurons. *Science (New York, NY).* 1993;260(5111):1130-1132.
73. Hagg T, Varon S. Ciliary neurotrophic factor prevents degeneration of adult rat substantia nigra dopaminergic neurons in vivo. *Proc Natl Acad Sci U S A.* 1993;90(13):6315-6319.
74. Kearns CM, Gash DM. GDNF protects nigral dopamine neurons against 6-hydroxydopamine in vivo. *Brain research.* 1995;672(1-2):104-111.
75. Choi-Lundberg DL, Lin Q, Schallert T, et al. Behavioral and cellular protection of rat dopaminergic neurons by an adenoviral vector encoding glial cell line-derived neurotrophic factor. *Experimental neurology.* 1998;154(2):261-275.
76. Kaigler D, Krebsbach PH, Polverini PJ, Mooney DJ. Role of vascular endothelial growth factor in bone marrow stromal cell modulation of endothelial cells. *Tissue Eng.* 2003;9(1):95-103.
77. Khoo ML, Tao H, Meedeniya AC, Mackay-Sim A, Ma DD. Transplantation of neuronal-primed human bone marrow mesenchymal stem cells in hemiparkinsonian rodents. *PLoS One.* 2011;6(5):e19025.
78. Batchelor PE, Liberatore GT, Wong JY, et al. Activated macrophages and microglia induce dopaminergic sprouting in the injured striatum and express brain-derived neurotrophic factor and glial cell line-derived neurotrophic factor. *J Neurosci.* 1999;19(5):1708-1716.
79. Munoz JR, Stoutenger BR, Robinson AP, Spees JL, Prockop DJ. Human stem/progenitor cells from bone marrow promote neurogenesis of endogenous neural stem cells in the hippocampus of mice. *Proc Natl Acad Sci U S A.* 2005;102(50):18171-18176.
80. Bai L, Caplan A, Lennon D, Miller RH. Human mesenchymal stem cells signals regulate neural stem cell fate. *Neurochem Res.* 2007;32(2):353-362.
81. Park HJ, Shin JY, Lee BR, Kim HO, Lee PH. Mesenchymal stem cells augment neurogenesis in the subventricular zone and enhance differentiation of neural precursor cells into dopaminergic neurons in the substantia nigra of a parkinsonian model. *Cell Transplant.* 2012;21(8):1629-1640.
82. Robinson AP, Foraker JE, Ylostalo J, Prockop DJ. Human stem/progenitor cells from bone marrow enhance glial differentiation of rat neural stem cells: a role for transforming growth factor beta and Notch signaling. *Stem Cells Dev.* 2011;20(2):289-300.
83. Stagg J. Immune regulation by mesenchymal stem cells: two sides to the coin. *Tissue Antigens.* 2007;69(1):1-9.
84. Ren G, Zhang L, Zhao X, et al. Mesenchymal stem cell-mediated immunosuppression occurs via concerted action of chemokines and nitric oxide. *Cell Stem Cell.* 2008;2(2):141-150.
85. Camp DM, Loeffler DA, Farrah DM, Borneman JN, LeWitt PA. Cellular immune response to intrastriatally implanted allogeneic bone marrow stromal cells in a rat model of Parkinson's disease. *J Neuroinflammation.* 2009;6:17.
86. Nauta AJ, Fibbe WE. Immunomodulatory properties of mesenchymal stromal cells. *Blood.* 2007;110(10):3499-3506.
87. Le Blanc K, Mougiakakos D. Multipotent mesenchymal stromal cells and the innate immune system. *Nat Rev Immunol.* 2012;12(5):383-396.

88. Shi Y, Su J, Roberts AI, Shou P, Rabson AB, Ren G. How mesenchymal stem cells interact with tissue immune responses. *Trends Immunol.* 2012;33(3):136-143.
89. Bernardo ME, Fibbe WE. Mesenchymal stromal cells: sensors and switchers of inflammation. *Cell Stem Cell.* 2013;13(4):392-402.
90. Yu B, Zhang X, Li X. Exosomes derived from mesenchymal stem cells. *International journal of molecular sciences.* 2014;15(3):4142-4157.
91. Huang S, Xu L, Sun Y, Zhang Y, Li G. The fate of systemically administrated allogeneic mesenchymal stem cells in mouse femoral fracture healing. *Stem cell research & therapy.* 2015;6:206.
92. Kidd S, Spaeth E, Dembinski JL, et al. Direct Evidence of Mesenchymal Stem Cell Tropism for Tumor and Wounding Microenvironments using In Vivo Bioluminescence Imaging. *Stem cells (Dayton, Ohio).* 2009;27(10):2614-2623.
93. Wang F, Yasuhara T, Shingo T, et al. Intravenous administration of mesenchymal stem cells exerts therapeutic effects on parkinsonian model of rats: Focusing on neuroprotective effects of stromal cell-derived factor-1alpha. *BMC Neuroscience.* 2010;11(1):52.
94. Jiang X, Liu C, Hao J, et al. CD4+CD25+ regulatory T cells are not required for mesenchymal stem cell function in fully MHC-mismatched mouse cardiac transplantation. *Cell Tissue Res.* 2014;358(2):503-514.
95. Nauta AJ, Westerhuis G, Kruisselbrink AB, Lurvink EG, Willemze R, Fibbe WE. Donor-derived mesenchymal stem cells are immunogenic in an allogeneic host and stimulate donor graft rejection in a nonmyeloablative setting. *Blood.* 2006;108(6):2114-2120.
96. Beggs KJ, Lyubimov A, Borneman JN, et al. Immunologic consequences of multiple, high-dose administration of allogeneic mesenchymal stem cells to baboons. *Cell Transplant.* 2006;15(8-9):711-721.
97. Campeau PM, Rafei M, Francois M, Birman E, Forner KA, Galipeau J. Mesenchymal stromal cells engineered to express erythropoietin induce anti-erythropoietin antibodies and anemia in allorecipients. *Mol Ther.* 2009;17(2):369-372.
98. Eliopoulos N, Stagg J, Lejeune L, Pommey S, Galipeau J. Allogeneic marrow stromal cells are immune rejected by MHC class I- and class II-mismatched recipient mice. *Blood.* 2005;106(13):4057-4065.
99. Schu S, Nosov M, O'Flynn L, et al. Immunogenicity of allogeneic mesenchymal stem cells. *J Cell Mol Med.* 2012;16(9):2094-2103.
100. Thorey F, Floerkemeier T, Wellmann M, Windhagen H. Comparison of a manual and motorized stiffness meter to quantify bone regeneration in distraction osteogenesis. *Technol Health Care.* 2009;17(5-6):369-375.
101. Zangi L, Margalit R, Reich-Zeliger S, et al. Direct imaging of immune rejection and memory induction by allogeneic mesenchymal stromal cells. *Stem Cells.* 2009;27(11):2865-2874.
102. Jiaming M, Niu C. Comparing neuroprotective effects of CDNF-expressing bone marrow derived mesenchymal stem cells via differing routes of administration utilizing an in vivo model of Parkinson's disease. *Neurol Sci.* 2014;36(2):281-287.
103. Oh SH, Kim HN, Park HJ, Shin JY, Kim DY, Lee PH. The Cleavage Effect of Mesenchymal Stem Cell and Its Derived Matrix Metalloproteinase-2 on Extracellular alpha-Synuclein Aggregates in Parkinsonian Models. *Stem cells translational medicine.* 2017;6(3):949-961.
104. Stemberger S, Jamnig A, Stefanova N, Lepperdinger G, Reindl M, Wenning GK. Mesenchymal stem cells in a transgenic mouse model of multiple system atrophy: immunomodulation and neuroprotection. *PLoS One.* 2011;6(5):e19808.
105. Chao YX, He BP, Tay SS. Mesenchymal stem cell transplantation attenuates blood brain barrier damage and neuroinflammation and protects dopaminergic neurons against MPTP toxicity in

the substantia nigra in a model of Parkinson's disease. *Journal of neuroimmunology.* 2009;216(1-2):39-50.

106. Venkataramana NK, Kumar SK, Balaraju S, et al. Open-labeled study of unilateral autologous bone-marrow-derived mesenchymal stem cell transplantation in Parkinson's disease. *Transl Res.* 2010;155(2):62-70.
107. Venkataramana NK, Pal R, Rao SA, et al. Bilateral transplantation of allogenic adult human bone marrow-derived mesenchymal stem cells into the subventricular zone of Parkinson's disease: a pilot clinical study. *Stem Cells Int.* 2012;2012:931902.
108. Dongmei H, Jing L, Mei X, et al. Clinical analysis of the treatment of spinocerebellar ataxia and multiple system atrophy-cerebellar type with umbilical cord mesenchymal stromal cells. *Cytotherapy.* 2011;13(8):913-917.
109. Xi H, Chen L, Huang H, et al. Preliminary report of multiple cell therapy for patients with multiple system atrophy. *Cell Transplant.* 2013;22 Suppl 1:S93-99.
110. Lee PH, Kim JW, Bang OY, Ahn YH, Joo IS, Huh K. Autologous mesenchymal stem cell therapy delays the progression of neurological deficits in patients with multiple system atrophy. *Clin Pharmacol Ther.* 2008;83(5):723-730.
111. Karussis D, Karageorgiou C, Vaknin-Dembinsky A, et al. Safety and immunological effects of mesenchymal stem cell transplantation in patients with multiple sclerosis and amyotrophic lateral sclerosis. *Arch Neurol.* 2010;67(10):1187-1194.
112. Connick P, Kolappan M, Crawley C, et al. Autologous mesenchymal stem cells for the treatment of secondary progressive multiple sclerosis: an open-label phase 2a proof-of-concept study. *Lancet Neurol.* 2012;11(2):150-156.
113. Liang J, Zhang H, Hua B, et al. Allogeneic mesenchymal stem cells transplantation in treatment of multiple sclerosis. *Mult Scler.* 2009;15(5):644-646.
114. Hou ZL, Liu Y, Mao XH, et al. Transplantation of umbilical cord and bone marrow-derived mesenchymal stem cells in a patient with relapsing-remitting multiple sclerosis. *Cell Adh Migr.* 2013;7(5):404-407.
115. Mazzini L, Mareschi K, Ferrero I, et al. Mesenchymal stromal cell transplantation in amyotrophic lateral sclerosis: a long-term safety study. *Cytotherapy.* 2012;14(1):56-60.
116. Nabavi SM, Arab L, Jarooghi N, et al. Safety, Feasibility of Intravenous and Intrathecal Injection of Autologous Bone Marrow Derived Mesenchymal Stromal Cells in Patients with Amyotrophic Lateral Sclerosis: An Open Label Phase I Clinical Trial. *Cell journal.* 2019;20(4):592-598.
117. Kurtzberg J, Prockop S, Teira P, et al. Allogeneic human mesenchymal stem cell therapy (remestemcel-L, Prochymal) as a rescue agent for severe refractory acute graft-versus-host disease in pediatric patients. *Biol Blood Marrow Transplant.* 2014;20(2):229-235.
118. Hare JM, Traverse JH, Henry TD, et al. A randomized, double-blind, placebo-controlled, dose-escalation study of intravenous adult human mesenchymal stem cells (prochymal) after acute myocardial infarction. *J Am Coll Cardiol.* 2009;54(24):2277-2286.
119. Wang P, Li Y, Huang L, et al. Effects and safety of allogenic mesenchymal stem cell intravenous infusion in active ankylosing spondylitis patients who failed NSAIDs: a 20-week clinical trial. *Cell Transplant.* 2014;23(10):1293-1303.
120. Wang D, Zhang H, Liang J, et al. Allogeneic mesenchymal stem cell transplantation in severe and refractory systemic lupus erythematosus: 4 years of experience. *Cell Transplant.* 2013;22(12):2267-2277.
121. Zhang Z, Fu J, Xu X, et al. Safety and immunological responses to human mesenchymal stem cell therapy in difficult-to-treat HIV-1-infected patients. *AIDS.* 2013;27(8):1283-1293.

122. Wang D, Akiyama K, Zhang H, et al. Double allogenic mesenchymal stem cells transplantations could not enhance therapeutic effect compared with single transplantation in systemic lupus erythematosus. *Clin Dev Immunol.* 2012;2012:273291.
123. Liang J, Zhang H, Hua B, et al. Allogenic mesenchymal stem cells transplantation in refractory systemic lupus erythematosus: a pilot clinical study. *Ann Rheum Dis.* 2010;69(8):1423-1429.
124. Forbes GM, Sturm MJ, Leong RW, et al. A phase 2 study of allogeneic mesenchymal stromal cells for luminal Crohn's disease refractory to biologic therapy. *Clinical gastroenterology and hepatology : the official clinical practice journal of the American Gastroenterological Association.* 2014;12(1):64-71.
125. Muroi K, Miyamura K, Ohashi K, et al. Unrelated allogeneic bone marrow-derived mesenchymal stem cells for steroid-refractory acute graft-versus-host disease: a phase I/II study. *Int J Hematol.* 2013;98(2):206-213.
126. Weiss DJ, Casaburi R, Flannery R, LeRoux-Williams M, Tashkin DP. A placebo-controlled, randomized trial of mesenchymal stem cells in COPD. *Chest.* 2013;143(6):1590-1598.
127. Smith KC, Suescun J, Bick DL, Schiess MC. Patterns of peripheral immune activity in prodromal asymptomatic and symptomatic Parkinsonism. *Mov Disord.* 2015;30 Suppl 1:S47.
128. Le Blanc K, Tammik C, Rosendahl K, Zetterberg E, Ringden O. HLA expression and immunologic properties of differentiated and undifferentiated mesenchymal stem cells. *Exp Hematol.* 2003;31(10):890-896.
129. Ankrum JA, Ong JF, Karp JM. Mesenchymal stem cells: immune evasive, not immune privileged. *Nat Biotechnol.* 2014;32(3):252-260.
130. Griffin MD, Ryan AE, Alagesan S, Lohan P, Treacy O, Ritter T. Anti-donor immune responses elicited by allogeneic mesenchymal stem cells: what have we learned so far? *Immunol Cell Biol.* 2013;91(1):40-51.
131. Lalu MM, McIntyre L, Pugliese C, et al. Safety of cell therapy with mesenchymal stromal cells (SafeCell): a systematic review and meta-analysis of clinical trials. *PLoS One.* 2012;7(10):e47559.
132. Hallek M, Cheson BD, Catovsky D, et al. iwCLL guidelines for diagnosis, indications for treatment, response assessment, and supportive management of CLL. *Blood.* 2018;131(25):2745-2760.
133. Holford NH, Chan PL, Nutt JG, Kieburtz K, Shoulson I, Parkinson Study G. Disease progression and pharmacodynamics in Parkinson disease - evidence for functional protection with levodopa and other treatments. *J Pharmacokinet Pharmacodyn.* 2006;33(3):281-311.
134. Schrag A, Sampaio C, Counsell N, Poewe W. Minimal clinically important change on the unified Parkinson's disease rating scale. *Mov Disord.* 2006;21(8):1200-1207.
135. Poewe W. Clinical measures of progression in Parkinson's disease. *Mov Disord.* 2009;24 Suppl 2:S671-676.
136. Parashos SA, Luo S, Biglan KM, et al. Measuring disease progression in early Parkinson disease: the National Institutes of Health Exploratory Trials in Parkinson Disease (NET-PD) experience. *JAMA Neurol.* 2014;71(6):710-716.
137. Schrag A, Dodel R, Spottke A, Bornschein B, Siebert U, Quinn NP. Rate of clinical progression in Parkinson's disease. A prospective study. *Mov Disord.* 2007;22(7):938-945.
138. Horvath K, Aschermann Z, Acs P, et al. Minimal clinically important difference on the Motor Examination part of MDS-UPDRS. *Parkinsonism Relat Disord.* 2015;21(12):1421-1426.
139. Holden SK, Finseth T, Sillau SH, Berman BD. Progression of MDS-UPDRS Scores Over Five Years in De Novo Parkinson Disease from the Parkinson's Progression Markers Initiative Cohort. *Movement disorders clinical practice.* 2018;5(1):47-53.
140. Schrag A, Spottke A, Quinn NP, Dodel R. Comparative responsiveness of Parkinson's disease scales to change over time. *Mov Disord.* 2009;24(6):813-818.

141. Peto V, Jenkinson C, Fitzpatrick R. Determining minimally important differences for the PDQ-39 Parkinson's disease questionnaire. *Age Ageing.* 2001;30(4):299-302.
142. McClure NS, Sayah FA, Xie F, Luo N, Johnson JA. Instrument-Defined Estimates of the Minimally Important Difference for EQ-5D-5L Index Scores. *Value in health : the journal of the International Society for Pharmacoeconomics and Outcomes Research.* 2017;20(4):644-650.
143. Fernandez-Seara MA, Mengual E, Vidorreta M, et al. Cortical hypoperfusion in Parkinson's disease assessed using arterial spin labeled perfusion MRI. *Neuroimage.* 2012;59(3):2743-2750.
144. Shahnawaz M, Tokuda T, Waragai M, et al. Development of a Biochemical Diagnosis of Parkinson Disease by Detection of alpha-Synuclein Misfolded Aggregates in Cerebrospinal Fluid. *JAMA Neurol.* 2017;74(2):163-172.
145. Merello M, Gerschcovich ER, Ballesteros D, Cerquetti D. Correlation between the Movement Disorders Society Unified Parkinson's Disease rating scale (MDS-UPDRS) and the Unified Parkinson's Disease rating scale (UPDRS) during L-dopa acute challenge. *Parkinsonism Relat Disord.* 2011;17(9):705-707.
146. Hanley PJ, Mei Z, Durett AG, et al. Efficient manufacturing of therapeutic mesenchymal stromal cells with the use of the Quantum Cell Expansion System. *Cytotherapy.* 2014;16(8):1048-1058.
147. Saborio GP, Permanne B, Soto C. Sensitive detection of pathological prion protein by cyclic amplification of protein misfolding. *Nature.* 2001;411(6839):810-813.
148. Ellmore TM, Hood AJ, Castriotta RJ, Stimming EF, Bick RJ, Schiess MC. Reduced volume of the putamen in REM sleep behavior disorder patients. *Parkinsonism Relat Disord.* 2010;16(10):645-649.
149. Wang J, Alsop DC, Song HK, et al. Arterial transit time imaging with flow encoding arterial spin tagging (FEAST). *Magn Reson Med.* 2003;50(3):599-607.
150. Ellmore TM, Murphy SM, Cruz K, Castriotta RJ, Schiess MC. Averaging of diffusion tensor imaging direction-encoded color maps for localizing substantia nigra. *Comput Biol Med.* 2014;51:104-110.
151. Chen X, Huddleston DE, Langley J, et al. Simultaneous imaging of locus coeruleus and substantia nigra with a quantitative neuromelanin MRI approach. *Magnetic resonance imaging.* 2014;32(10):1301-1306.
152. O'Neill RT. FDA's critical path initiative: a perspective on contributions of biostatistics. *Biom J.* 2006;48(4):559-564.
153. Shulman LM, Gruber-Baldini AL, Anderson KE, Fishman PS, Reich SG, Weiner WJ. The clinically important difference on the unified Parkinson's disease rating scale. *Arch Neurol.* 2010;67(1):64-70.

Appendix A. UK PARKINSON'S DISEASE BRAIN BANK CRITERIA

Step 1. Diagnosis of Parkinsonian Syndrome

- Bradykinesia
- At least one of the following
  - Muscular rigidity
  - 4-6 Hz rest tremor
  - postural instability not caused by primary visual, vestibular, cerebellar, or proprioceptive dysfunction

Step 2 Exclusion criteria for Parkinson's disease

- history of repeated strokes with stepwise progression of parkinsonian features
- history of repeated head injury
- history of definite encephalitis
- oculogyric crises
- neuroleptic treatment at onset of symptoms
- more than one affected relative
- sustained remission
- strictly unilateral features after 3 years
- supranuclear gaze palsy
- cerebellar signs
- early severe autonomic involvement
- early severe dementia with disturbances of memory, language, and praxis
- Babinski sign
- presence of cerebral tumor or communication hydrocephalus on imaging study
- negative response to large doses of levodopa in absence of malabsorption
- MPTP exposure

Step 3 supportive prospective positive criteria for Parkinson's disease
Three or more required for diagnosis of definite Parkinson's disease in combination with step one

- Unilateral onset
- Rest tremor present
- Progressive disorder
- Persistent asymmetry affecting side of onset most
- Excellent response (70-100%) to levodopa
- Severe levodopa-induced chorea
- Levodopa response for 5 years or more
- Clinical course of ten years or more

****From: Hughes AJ, Daniel SE, Kilford L, Lees AJ. Accuracy of clinical diagnosis of idiopathic Parkinson's disease. A clinico-pathological study of 100 cases. JNNP 1992;55:181-184.***

Appendix B. Modified Hoehn and Yahr

Modified Hoehn and Yahr Scale
1.0: Unilateral involvement only
1.5: Unilateral and axial involvement
2.0: Bilateral involvement without impairment of balance
2.5: Mild bilateral disease with recovery on pull test
3.0: Mild to moderate bilateral disease; some postural instability; physically independent
4.0: Severe disability; still able to walk or stand unassisted
5.0: Wheelchair bound or bedridden unless aided

Appendix C. MoCA

MONTREAL COGNITIVE ASSESSMENT (MoCA ®)
Version 8.3 English

Name:
Education:
Sex:
Date of birth:
DATE:

Section	Content	POINTS
VISUOSPATIAL / EXECUTIVE	Trail (Begin 1 → A → 2 … End E) [ ] ; Copy bed [ ] ; Draw CLOCK (Five past ten) (3 points) [ ] Contour [ ] Numbers [ ] Hands	___/5
NAMING	[ ] [ ] [ ]	___/3

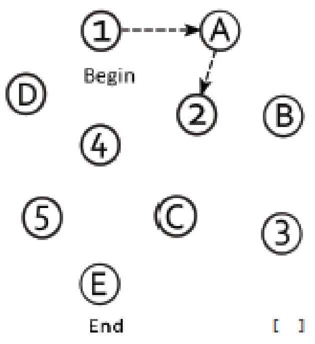

Copy bed

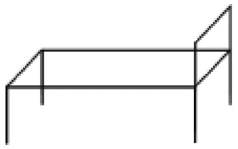

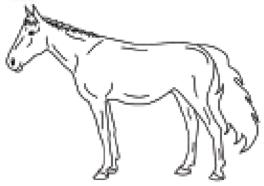

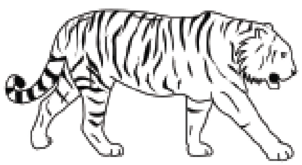

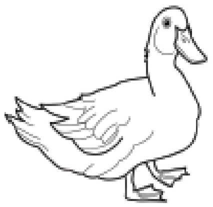

MEMORY
Read list of words, subject must repeat them. Do 2 trials, even if 1st trial is successful. Do a recall after 5 minutes.

	LEG	COTTON	SCHOOL	TOMATO	WHITE
1st TRIAL					
2nd TRIAL					

NO POINTS

ATTENTION
Read list of digits (1 digit / sec.).
Subject has to repeat them in the forward order. [ ] 2 4 8 1 5
Subject has to repeat them in the backward order. [ ] 4 2 7 ___/2

Read list of letters. The subject must tap with his hand at each letter A. No points if ≥ 2 errors.
[ ] F B A C M N A A J K L B A F A K D E A A A J A M O F A A B ___/1

Serial 7 subtraction starting at 60. [ ] 53 [ ] 46 [ ] 39 [ ] 32 [ ] 25
4 or 5 correct subtractions: 3 pts, 2 or 3 correct: 2 pts, 1 correct: 1 pt, 0 correct: 0 pt ___/3

LANGUAGE
Repeat: The child walked his dog in the park after midnight. [ ]
The artist finished his painting at the right moment for the exhibition. [ ] ___/2

Language Fluency. Name maximum number of words in one minute that begin with the letter B. [ ] ______ (N ≥ 11 words) ___/1

ABSTRACTION
Similarity between e.g. banana - orange = fruit [ ] hammer - screwdriver [ ] matches - lamp ___/2

DELAYED RECALL

(MIS)	Has to recall words	LEG	COTTON	SCHOOL	TOMATO	WHITE	
X3	WITH NO CUE	[ ]	[ ]	[ ]	[ ]	[ ]	Points for UNCUED recall only ___/5
X2	Category cue						
X1	Multiple choice cue						MIS = ____/15

Memory Index Score (MIS)

ORIENTATION
[ ] Date [ ] Month [ ] Year [ ] Day [ ] Place [ ] City ___/6

© Z. Nasreddine MD www.mocatest.org
MIS: /15
(Normal ≥ 26/30)
Add 1 point if ≤ 12 yr education
TOTAL ___/30

Administered by: ____________________

Training and Certification are required to ensure accuracy.

Appendix D. MSC label sample

LABELS

Partial Label for Final Product
To be used during preparation for administration

MSC, CULTURED, THAWED & WASHED
LOT#: C2334.5 Expiration: 16.00h 03/14/16
RECIPIENT: DOE, John HeMH 0987654 P12345
For use by intended recipient only
CAUTION: New drug for investigational use only

Full Label
To be used for final product

TC-MSC CULTURED - Thawed and Washed,

Component #:	Collection Date and Time	Nonreactive for HBsAg by FDA required test. Non-reactive for syphilis by STS. Negative by a test for antibody to HIV. No unexpected antibodies detected.	n/a
C2334.5	13.00h 11/06/12		
	Expiration Date and Time		
	17.00h 11/06/12		Rh n/a

Total Vol. (ml): ______
Total Cells ______
Recipient Wt:(kg) 75

DO NOT IRRADIATE
DO NOT USE LEUKO-REDUCTION FILTER

Product Contains	Vol / Units
Plasma Lyte	50ml

Recipient: Doe, John
Recipient MR#: TCH0987654
Recipient CAGT#: P12345

CAUTION: NEW DRUG - LIMITED BY FEDERAL LAW TO INVESTIGATIONAL USE

VOLUNTEER DONOR
WARNING. This product may transmit infectious agents.
Caution: Federal law prohibits dispensing without a prescription
PROPERLY IDENTIFY INTENDED RECIPIENT AND PRODUCT
ALLOGENEIC

Donor Name: XXXX-XXXX
Donor MR#: n/a
Donor CMT#: n/a

FOR USE BY INTENDED RECIPIENT ONLY

Processed By: CAGT GMP Facility, Baylor College of Medicine
16th Floor Feigin Center, 1102 Bates St., Houston, TX 77030
CLIA ID#: 45D0958729
L07.02

Appendix E. Medication Form

MEDICATION FORM					
**Patient's name:**	Scr Date (mm/dd/yy)	Inf #1 Date (mm/dd/yy)	Inf #2 Date (mm/dd/yy)	Inf#3 Date (mm/dd/yy)	DOB(mm/dd/yy):
**Subject ID:**	**Allergies:**				

**Visit #***	**Medication Name** (List generic name if possible)	**Dosage Units** (mg, drops, mcg, puffs, etc)	**Frequency** (QD, BID, TID, QID, QOD, one time, prn, once per week/month)	**Route** (PO, topical, IM, IV, SC, rectal, intranasal, intraocular)	**Indication**	**X if for PD**	**Start Date**	**Stop Date** (X if ongoing)	**Reason, if changed** (increase, decrease, DC)

*Visit #: 1 = Scr, 2 =BL, 3 = inf # 1, 4 = wk 7, 5 = inf# 2, 6 = wk 20, 7 = inf# 3, 8 = wk 29, 9 = wk 39, 10 = wk 52, 11 = wk 78, or phone visit = Pv date

Appendix F. Modified Schwab and England Activities of Daily Living Scale (ADL)

*Score: __________ (Number between 1-100)

100% – Completely independent. Able to do all chores without slowness, difficulty or impairment. Essentially normal. Unaware of any difficulty.

90% – Completely independent. Able to do all chores with some degree of slowness, difficulty and impairment. Might take twice as long. Beginning to be aware of difficulty.

80% – Completely independent in most chores. Takes twice as long. Conscious of difficulty and slowness.

70% – Not completely independent. More difficulty with some chores. Three to four times as long in some. Must spend a large part of the day with chores.

60% – Some dependency. Can do most chores, but exceedingly slowly and with much effort. Errors; some impossible.

50% – More dependent. Help with half, slower, et cetera. Difficulty with everything.

40% – Very dependent. Can assist with all chores, but few alone.

30% – With effort, now and then does a few chores alone or begins alone. Much help needed.

20% – Nothing alone. Can be a slight help with some chores. Severe invalid.

10% – Total dependent, helpless. Complete invalid.

0% – Vegetative functions such as swallowing, bladder and bowel functions are not functioning. Bed-ridden.

Appendix G. Parkinson's disease Questionnaire (PDQ-39)

Please complete the following

Please tick one box for each question

Due to having Parkinson's disease, how often during the last month have you....		**Never**	**Occasionally**	**Sometimes**	**Often**	**Always** or cannot do at all
1	Had difficulty doing the leisure activities which you would like to do?	☐	☐	☐	☐	☐
2	Had difficulty looking after your home, e.g. DIY, housework, cooking?	☐	☐	☐	☐	☐
3	Had difficulty carrying bags of shopping?	☐	☐	☐	☐	☐
4	Had problems walking half a mile?	☐	☐	☐	☐	☐
5	Had problems walking 100 yards?	☐	☐	☐	☐	☐
6	Had problems getting around the house as easily as you would like?	☐	☐	☐	☐	☐
7	Had difficulty getting around in public?	☐	☐	☐	☐	☐
8	Needed someone else to accompany you when you went out?	☐	☐	☐	☐	☐
9	Felt frightened or worried about falling over in public?	☐	☐	☐	☐	☐
10	Been confined to the house more than you would like?	☐	☐	☐	☐	☐
11	Had difficulty washing yourself?	☐	☐	☐	☐	☐
12	Had difficulty dressing yourself?	☐	☐	☐	☐	☐
13	Had problems doing up your shoe laces?	☐	☐	☐	☐	☐

*Please check that you have ticked **one box for each question** before going on to the next page*

Due to having Parkinson's disease, how often <u>during the last month</u> have you….	**Never**	**Occasionally**	**Sometimes**	**Often**	**Always** or cannot do at all
	Please tick <u>one</u> box for each question				
14 Had problems writing clearly?	☐	☐	☐	☐	☐
15 Had difficulty cutting up your food?	☐	☐	☐	☐	☐
16 Had difficulty holding a drink without spilling it?	☐	☐	☐	☐	☐
17 Felt depressed?	☐	☐	☐	☐	☐
18 Felt isolated and lonely?	☐	☐	☐	☐	☐
19 Felt weepy or tearful?	☐	☐	☐	☐	☐
20 Felt angry or bitter?	☐	☐	☐	☐	☐
21 Felt anxious?	☐	☐	☐	☐	☐
22 Felt worried about your future?	☐	☐	☐	☐	☐
23 Felt you had to conceal your Parkinson's from people?	☐	☐	☐	☐	☐
24 Avoided situations which involve eating or drinking in public?	☐	☐	☐	☐	☐
25 Felt embarrassed in public due to having Parkinson's disease?	☐	☐	☐	☐	☐
26 Felt worried by other people's reaction to you?	☐	☐	☐	☐	☐
27 Had problems with your close personal relationships?	☐	☐	☐	☐	☐
28 Lacked support in the ways you need from your spouse or partner?	☐	☐	☐	☐	☐
If you do not have a spouse or partner tick here		☐			
29 Lacked support in the ways you need from your family or close friends?	☐	☐	☐	☐	☐

Please check that you have ticked ***one box for each question*** *before going on to the next page*

Due to having Parkinson's disease, how often ***during the last month*** have you....		Never	Occasionally	Sometimes	Often	Always
30	Unexpectedly fallen asleep during the day?	☐	☐	☐	☐	☐
31	Had problems with your concentration, e.g. when reading or watching TV?	☐	☐	☐	☐	☐
32	Felt your memory was bad?	☐	☐	☐	☐	☐
33	Had distressing dreams or hallucinations?	☐	☐	☐	☐	☐
34	Had difficulty with your speech?	☐	☐	☐	☐	☐
35	Felt unable to communicate with people properly?	☐	☐	☐	☐	☐
36	Felt ignored by people?	☐	☐	☐	☐	☐
37	Had painful muscle cramps or spasms?	☐	☐	☐	☐	☐
38	Had aches and pains in your joints or body?	☐	☐	☐	☐	☐
39	Felt unpleasantly hot or cold?	☐	☐	☐	☐	☐

Please tick one box for each question

Please check that you have ticked ***one box for each question*** *before going on to the next page*

Thank you for completing the PDQ 39 questionnaire

Appendix H. EQ-5D-5l

Under each heading, please tick the ONE box that best describes your health TODAY.

MOBILITY

I have no problems in walking about ☐
I have slight problems in walking about ☐
I have moderate problems in walking about ☐
I have severe problems in walking about ☐
I am unable to walk about ☐

SELF-CARE

I have no problems washing or dressing myself ☐
I have slight problems washing or dressing myself ☐
I have moderate problems washing or dressing myself ☐
I have severe problems washing or dressing myself ☐
I am unable to wash or dress myself ☐

USUAL ACTIVITIES *(e.g. work, study, housework, family or leisure activities)*

I have no problems doing my usual activities ☐
I have slight problems doing my usual activities ☐
I have moderate problems doing my usual activities ☐
I have severe problems doing my usual activities ☐
I am unable to do my usual activities ☐

PAIN / DISCOMFORT

I have no pain or discomfort ☐
I have slight pain or discomfort ☐
I have moderate pain or discomfort ☐
I have severe pain or discomfort ☐
I have extreme pain or discomfort ☐

ANXIETY / DEPRESSION

I am not anxious or depressed ☐
I am slightly anxious or depressed ☐
I am moderately anxious or depressed ☐
I am severely anxious or depressed ☐
I am extremely anxious or depressed ☐

- We would like to know how good or bad your health is TODAY.
- This scale is numbered from 0 to 100.
- 100 means the <u>best</u> health you can imagine.
  0 means the <u>worst</u> health you can imagine.
- Mark an X on the scale to indicate how your health is TODAY.
- Now, please write the number you marked on the scale in the box below.

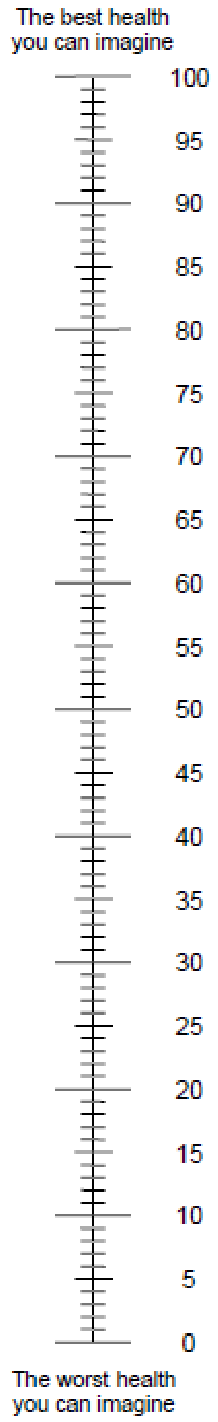

YOUR HEALTH TODAY = [ ]

Appendix I. C-SSRS, Lifetime version

COLUMBIA-SUICIDE SEVERITY

RATING SCALE

(C-SSRS)

Lifetime Recent - Clinical

Version 1/14/09

Posner, K.; Brent, D.; Lucas, C.; Gould, M.; Stanley, B.; Brown, G.; Fisher, P.; Zelazny, J.; Burke, A.; Oquendo, M.; Mann, J.

Disclaimer:

This scale is intended to be used by individuals who have received training in its administration. The questions contained in the Columbia-Suicide Severity Rating Scale are suggested probes. Ultimately, the determination of the presence of suicidal ideation or behavior depends on the judgment of the individual administering the scale.

Definitions of behavioral suicidal events in this scale are based on those used in ***The Columbia Suicide History Form****, developed by John Mann, MD and Maria Oquendo, MD, Conte Center for the Neuroscience of Mental Disorders (CCNMD), New York State Psychiatric Institute, 1051 Riverside Drive, New York, NY, 10032. (Oquendo M. A., Halberstam B. & Mann J. J., Risk factors for suicidal behavior: utility and limitations of research instruments. In M.B. First [Ed.]* Standardized Evaluation in Clinical Practice*, pp. 103 -130, 2003.)*

For reprints of the C-SSRS contact Kelly Posner, Ph.D., New York State Psychiatric Institute, 1051 Riverside Drive, New York, New York, 10032; inquiries and training requirements contact posnerk@nyspi.columbia.edu
© 2008 The Research Foundation for Mental Hygiene, Inc.

SUICIDAL IDEATION		
Ask questions 1 and 2. If both are negative, proceed to "Suicidal Behavior" section. If the answer to question 2 is "yes", ask questions 3, 4 and 5. If the answer to question 1 and/or 2 is "yes", complete "Intensity of Ideation" section below.	**Lifetime: Time He/She Felt Most Suicidal**	**Past 1 month**
**1. Wish to be Dead**<br><br>Subject endorses thoughts about a wish to be dead or not alive anymore, or wish to fall asleep and not wake up.<br><br>***Have you wished you were dead or wished you could go to sleep and not wake up?***<br><br>If yes, describe:	**Yes** **No**<br>☐ ☐	**Yes** **No**<br>☐ ☐
**2. Non-Specific Active Suicidal Thoughts**<br><br>General non-specific thoughts of wanting to end one's life/commit suicide (e.g., *"I've thought about killing myself"*) without thoughts of ways to kill oneself/associated methods, intent, or plan during the assessment period.<br><br>***Have you actually had any thoughts of killing yourself?***<br><br>If yes, describe:	**Yes** **No**<br>☐<br>☐	**Yes** **No**<br>☐ ☐
**3. Active Suicidal Ideation with Any Methods (Not Plan) without Intent to Act**<br><br>Subject endorses thoughts of suicide and has thought of at least one method during the assessment period. This is different than a specific plan with time, place or method details worked out (e.g., thought of method to kill self but not a specific plan). Includes person who would say, *"I thought about taking an overdose but I never made a specific plan as to when, where or how I would actually do it…and I would never go through with it."*<br><br>***Have you been thinking about how you might do this?***<br><br>If yes, describe:	**Yes** **No**<br>☐ ☐	**Yes** **No**<br>☐ ☐
**4. Active Suicidal Ideation with Some Intent to Act, without Specific Plan**<br><br>Active suicidal thoughts of killing oneself and subject reports having <u>some intent to act on such thoughts</u>, as opposed to *"I have the thoughts but I definitely will not do anything about them."*<br><br>***Have you had these thoughts and had some intention of acting on them?***<br><br>If yes, describe:	**Yes** **No**<br>☐ ☐	**Yes** **No**<br>☐ ☐

**5. Active Suicidal Ideation with Specific Plan and Intent**<br><br>Thoughts of killing oneself with details of plan fully or partially worked out and subject has some intent to carry it out.<br><br>***Have you started to work out or worked out the details of how to kill yourself? Do you intend to carry out this plan?***<br><br>If yes, describe:	**Yes** **No**<br>☐ ☐	**Yes** **No**<br>☐ ☐
**INTENSITY OF IDEATION**		
The following features should be rated with respect to the most severe type of ideation (i.e., 1-5 from above, with 1 being the least severe and 5 being the most severe). Ask about time he/she was feeling the most suicidal.<br><br>Lifetime - ***Most Severe Ideation:*** _______ ______________________________________<br>***Type # (1-5)*** ***Description of Ideation***<br><br>Recent - ***Most Severe Ideation:*** _______ ______________________________________<br>***Type # (1-5)*** ***Description of Ideation***	*Most Severe*	*Most Severe*
**Frequency**<br><br>***How many times have you had these thoughts?***<br><br>(1) Less than once a week (2) Once a week (3) 2-5 times in week (4) Daily or almost daily (5) Many times each day	____	____
**Duration**<br><br>***When you have the thoughts how long do they last?***<br><br>(1) Fleeting - few seconds or minutes<br>(2) Less than 1 hour/some of the time<br>(3) 1-4 hours/a lot of time<br>(4) 4-8 hours/most of day<br>(5) More than 8 hours/persistent or continuous	____	____

**Controllability**<br><br>***Could/can you stop thinking about killing yourself or wanting to die if you want to?***<br><br>(1) Easily able to control thoughts (4) Can control thoughts with a lot of difficulty<br><br>(2) Can control thoughts with little difficulty (5) Unable to control thoughts<br><br>(3) Can control thoughts with some difficulty (0) Does not attempt to control thoughts	____	____
**Deterrents**<br><br>***Are there things - anyone or anything (e.g., family, religion, pain of death) - that stopped you from wanting to die or acting on thoughts of committing suicide?***<br><br>(1) Deterrents definitely stopped you from attempting suicide (4) Deterrents most likely did not stop you<br><br>(2) Deterrents probably stopped you (5) Deterrents definitely did not stop you<br><br>(3) Uncertain that deterrents stopped you (0) Does not apply	____	____
**Reasons for Ideation**<br><br>***What sort of reasons did you have for thinking about wanting to die or killing yourself? Was it to end the pain or stop the way you were feeling (in other words you couldn't go on living with this pain or how you were feeling) or was it to get attention, revenge or a reaction from others? Or both?***<br><br>(1) Completely to get attention, revenge or a reaction from others (4) Mostly to end or stop the pain (you couldn't go on<br><br>(2) Mostly to get attention, revenge or a reaction from others living with the pain or how you were feeling)<br><br>(3) Equally to get attention, revenge or a reaction from others (5) Completely to end or stop the pain (you couldn't go on<br><br>and to end/stop the pain living with the pain or how you were feeling)<br><br>(0) Does not apply	____	____
SUICIDAL BEHAVIOR<br><br>*(Check all that apply, so long as these are separate events; must ask about all types)*	**Lifetime**	**Past 3 months**

**Actual Attempt:**<br><br>A potentially self-injurious act committed with at least some wish to die, *as a result of act.* Behavior was in part thought of as method to kill oneself. Intent does not have to be 100%. If there is ***any*** intent/desire to die associated with the act, then it can be considered an actual suicide attempt. ***There does not have to be any injury or harm***, just the potential for injury or harm. If person pulls trigger while gun is in mouth but gun is broken so no injury results, this is considered an attempt.<br><br>Inferring Intent: Even if an individual denies intent/wish to die, it may be inferred clinically from the behavior or circumstances. For example, a highly lethal act that is clearly not an accident so no other intent but suicide can be inferred (e.g., gunshot to head, jumping from window of a high floor/story). Also, if someone denies intent to die, but they thought that what they did could be lethal, intent may be inferred.<br><br>***Have you made a suicide attempt?***<br><br>***Have you done anything to harm yourself?***<br><br>***Have you done anything dangerous where you could have died?***<br><br>***What did you do?***<br><br>***Did you______ as a way to end your life?***<br><br>***Did you want to die (even a little) when you_____?***<br><br>***Were you trying to end your life when you _____?***<br><br>***Or Did you think it was possible you could have died from_____?***<br><br>***Or did you do it purely for other reasons / without ANY intention of killing yourself (like to relieve stress, feel better,***<br><br>***get sympathy, or get something else to happen)?*** (Self-Injurious Behavior without suicidal intent)<br><br>If yes, describe:<br><br>**Has subject engaged in Non-Suicidal Self-Injurious Behavior?**	**Yes** **No**<br>☐ ☐<br><br>Total # of<br>Attempts<br>______<br><br>**Yes** **No**<br>☐ ☐	**Yes** **No**<br>☐ ☐<br><br>Total # of<br>Attempts<br>______<br><br>**Yes** **No**<br>☐ ☐
**Interrupted Attempt:**<br><br>When the person is interrupted (by an outside circumstance) from starting the potentially self-injurious act *(if not for that, actual attempt would have occurred).*<br><br>Overdose: Person has pills in hand but is stopped from ingesting. Once they ingest any pills, this becomes an attempt rather than an interrupted attempt. Shooting: Person has gun pointed toward self, gun is taken away by someone else, or is somehow prevented from pulling trigger. Once they pull the trigger, even if the gun fails to fire, it is an attempt. Jumping: Person is poised to jump, is grabbed and taken down from ledge. Hanging: Person has noose around neck but has not yet started to hang - is stopped from doing so.	**Yes** **No**<br>☐ ☐	**Yes** **No**<br>☐ ☐

Has there been a time when you started to do something to end your life but someone or something stopped you before you actually did anything?<br><br>If yes, describe:	Total # of interrupted<br><br>______	Total # of interrupted<br><br>______
**Aborted or Self-Interrupted Attempt:**<br><br>When person begins to take steps toward making a suicide attempt, but stops themselves before they actually have engaged in any self-destructive behavior. Examples are similar to interrupted attempts, except that the individual stops him/herself, instead of being stopped by something else.<br><br>***Has there been a time when you started to do something to try to end your life but you stopped yourself before you actually did anything?***<br><br>If yes, describe:	**Yes** **No**<br>☐ ☐<br><br>Total # of<br><br>aborted or self-interrupted<br><br>______	**Yes** **No**<br>☐ ☐<br><br>Total # of<br><br>aborted or self-interrupted<br><br>______
**Preparatory Acts or Behavior:**<br><br>Acts or preparation towards imminently making a suicide attempt. This can include anything beyond a verbalization or thought, such as assembling a specific method (e.g., buying pills, purchasing a gun) or preparing for one's death by suicide (e.g., giving things away, writing a suicide note).<br><br>***Have you taken any steps towards making a suicide attempt or preparing to kill yourself (such as collecting pills, getting a gun, giving valuables away or writing a suicide note)?***<br><br>If yes, describe:	**Yes** **No**<br>☐ ☐<br><br>Total # of preparatory acts<br><br>______	**Yes** **No**<br>☐ ☐<br><br>Total # of preparator y acts<br><br>______

	Most Recent Attempt<br><br>Date:	Most Lethal Attempt<br><br>Date:	Initial/First Attempt<br><br>Date:
**Actual Lethality/Medical Damage:**<br><br>0. No physical damage or very minor physical damage (e.g., surface scratches).<br><br>1. Minor physical damage (e.g., lethargic speech; first-degree burns; mild bleeding; sprains).<br><br>2. Moderate physical damage; medical attention needed (e.g., conscious but sleepy, somewhat responsive; second-degree burns; bleeding of major vessel).	*Enter Code*	*Enter Code*	*Enter Code*

3. Moderately severe physical damage; *medical* hospitalization and likely intensive care required (e.g., comatose with reflexes intact; third-degree burns less than 20% of body; extensive blood loss but can recover; major fractures).<br><br>4. Severe physical damage; *medical* hospitalization with intensive care required (e.g., comatose without reflexes; third-degree burns over 20% of body; extensive blood loss with unstable vital signs; major damage to a vital area).<br><br>5. Death	_______	_______	_______
**Potential Lethality: Only Answer if Actual Lethality=0**<br><br>Likely lethality of actual attempt if no medical damage (the following examples, while having no actual medical damage, had potential for very serious lethality: put gun in mouth and pulled the trigger but gun fails to fire so no medical damage; laying on train tracks with oncoming train but pulled away before run over).<br><br>0 = Behavior not likely to result in injury<br><br>1 = Behavior likely to result in injury but not likely to cause death<br><br>2 = Behavior likely to result in death despite available medical care	*Enter Code*<br><br>_______	*Enter Code*<br><br>_______	*Enter Code*<br><br>_______

Appendix J. C-SSRS, Last visit version

COLUMBIA-SUICIDE SEVERITY

RATING SCALE

(C-SSRS)

Since Last Visit - Clinical

Version 1/14/09

Posner, K.; Brent, D.; Lucas, C.; Gould, M.; Stanley, B.; Brown, G.; Fisher, P.; Zelazny, J.; Burke, A.; Oquendo, M.; Mann, J.

Disclaimer:

This scale is intended to be used by individuals who have received training in its administration. The questions contained in the Columbia-Suicide Severity Rating Scale are suggested probes. Ultimately, the determination of the presence of suicidal ideation or behavior depends on the judgment of the individual administering the scale.

Definitions of behavioral suicidal events in this scale are based on those used in ***<u>The Columbia Suicide History Form</u>****, developed by John Mann, MD and Maria Oquendo, MD, Conte Center for the Neuroscience of Mental Disorders (CCNMD), New York State Psychiatric Institute, 1051 Riverside Drive, New York, NY, 10032. (Oquendo M. A., Halberstam B. & Mann J. J., Risk factors for suicidal behavior: utility and limitations of research instruments. In M.B. First [Ed.]* Standardized Evaluation in Clinical Practice*, pp. 103 -130, 2003.)*

For reprints of the C-SSRS contact Kelly Posner, Ph.D., New York State Psychiatric Institute, 1051 Riverside Drive, New York, New York, 10032; inquiries and training requirements contact posnerk@nyspi.columbia.edu

© 2008 The Research Foundation for Mental Hygiene, Inc.

SUICIDAL IDEATION

Ask questions 1 and 2. If both are negative, proceed to "Suicidal Behavior" section. If the answer to question 2 is "yes", ask questions 3, 4 and 5. If the answer to question 1 and/or 2 is "yes", complete "Intensity of Ideation" section below.	Since Last Visit
**1. Wish to be Dead**<br><br>Subject endorses thoughts about a wish to be dead or not alive anymore, or wish to fall asleep and not wake up.<br><br>***Have you wished you were dead or wished you could go to sleep and not wake up?***<br><br>If yes, describe:	**Yes** **No**<br>☐ ☐
**2. Non-Specific Active Suicidal Thoughts**<br><br>General, non-specific thoughts of wanting to end one's life/commit suicide (e.g., *"I've thought about killing myself"*) without thoughts of ways to kill oneself/associated methods, intent, or plan during the assessment period.<br><br>***Have you actually had any thoughts of killing yourself?***<br><br>If yes, describe:	**Yes** **No**<br>☐ ☐
**3. Active Suicidal Ideation with Any Methods (Not Plan) without Intent to Act**<br><br>Subject endorses thoughts of suicide and has thought of at least one method during the assessment period. This is different than a specific plan with time, place or method details worked out (e.g., thought of method to kill self but not a specific plan). Includes person who would say, *"I thought about taking an overdose but I never made a specific plan as to when, where or how I would actually do it…and I would never go through with it."*<br><br>***Have you been thinking about how you might do this?***<br><br>If yes, describe:	**Yes** **No**<br>☐ ☐
**4. Active Suicidal Ideation with Some Intent to Act, without Specific Plan**<br><br>Active suicidal thoughts of killing oneself and subject reports having some intent to act on such thoughts, as opposed to *"I have the thoughts but I definitely will not do anything about them."*<br><br>***Have you had these thoughts and had some intention of acting on them?***<br><br>If yes, describe:	**Yes** **No**<br>☐ ☐

**5. Active Suicidal Ideation with Specific Plan and Intent**<br>Thoughts of killing oneself with details of plan fully or partially worked out and subject has some intent to carry it out.<br>***Have you started to work out or worked out the details of how to kill yourself? Do you intend to carry out this plan?***<br>If yes, describe:	**Yes** ☐ **No** ☐
INTENSITY OF IDEATION	
The following features should be rated with respect to the most severe type of ideation (i.e., 1-5 from above, with 1 being the least severe and 5 being the most severe).<br>***Most Severe Ideation:*** _____<br>____<br>***Type # (1-5)*** ***Description of Ideation***	Most Severe
**Frequency**<br>***How many times have you had these thoughts?***<br>(1) Less than once a week (2) Once a week (3) 2-5 times in week (4) Daily or almost daily (5) Many times each day	____
**Duration**<br>***When you have the thoughts, how long do they last?***<br>(1) Fleeting - few seconds or minutes<br>(2) Less than 1 hour/some of the time<br>(3) 1-4 hours/a lot of time<br>(4) 4-8 hours/most of day<br>(5) More than 8 hours/persistent or continuous	____
**Controllability**<br>***Could/can you stop thinking about killing yourself or wanting to die if you want to?***<br>(1) Easily able to control thoughts<br>(2) Can control thoughts with little difficulty<br>(3) Can control thoughts with some difficulty<br>(4) Can control thoughts with a lot of difficulty<br>(5) Unable to control thoughts<br>(0) Does not attempt to control thoughts	____
**Deterrents**<br>***Are there things - anyone or anything (e.g., family, religion, pain of death) - that stopped you from wanting to die or acting on thoughts of committing suicide?***	____

(1) Deterrents definitely stopped you from attempting suicide (4) Deterrents most likely did not stop you<br>(2) Deterrents probably stopped you (5) Deterrents definitely did not stop you<br>(3) Uncertain that deterrents stopped you (0) Does not apply	
**Reasons for Ideation**<br>***What sort of reasons did you have for thinking about wanting to die or killing yourself? Was it to end the pain or stop the way you were feeling (in other words you couldn't go on living with this pain or how you were feeling) or was it to get attention, revenge or a reaction from others? Or both?***<br>(1) Completely to get attention, revenge or a reaction from others (4) Mostly to end or stop the pain (you couldn't go on<br>(2) Mostly to get attention, revenge or a reaction from others living with the pain or how you were feeling)<br>(3) Equally to get attention, revenge or a reaction from others (5) Completely to end or stop the pain (you couldn't go on<br>and to end/stop the pain living with the pain or how you were feeling)<br>(0) Does not apply	____
SUICIDAL BEHAVIOR<br>*(Check all that apply, so long as these are separate events; must ask about all types)*	Since Last Visit
**Actual Attempt:**<br>A potentially self-injurious act committed with at least some wish to die, *as a result of act.* Behavior was in part thought of as method to kill oneself. Intent does not have to be 100%. If there is ***any*** intent/desire to die associated with the act, then it can be considered an actual suicide attempt. ***There does not have to be any injury or harm***, just the potential for injury or harm. If person pulls trigger while gun is in mouth but gun is broken so no injury results, this is considered an attempt.<br>Inferring Intent: Even if an individual denies intent/wish to die, it may be inferred clinically from the behavior or circumstances. For example, a highly lethal act that is clearly not an accident so no other intent but suicide can be inferred (e.g., gunshot to head, jumping from window of a high floor/story). Also, if someone denies intent to die, but they thought that what they did could be lethal, intent may be inferred.<br>***Have you made a suicide attempt?***<br>***Have you done anything to harm yourself?***<br>***Have you done anything dangerous where you could have died?***<br>***What did you do?***<br>***Did you______ as a way to end your life?***<br>***Did you want to die (even a little) when you_____?***<br>***Were you trying to end your life when you _____?***<br>***Or did you think it was possible you could have died from_____?***	**Yes** **No**<br>☐ ☐<br>Total # of Attempts<br>_____

Or did you do it purely for other reasons / without ANY intention of killing yourself (like to relieve stress, feel better, get sympathy, or get something else to happen)? (Self-Injurious Behavior without suicidal intent)<br><br>If yes, describe:<br><br>**Has subject engaged in Non-Suicidal Self-Injurious Behavior?**	**Yes** **No**<br><br>☐ ☐
**Interrupted Attempt:**<br><br>When the person is interrupted (by an outside circumstance) from starting the potentially self-injurious act *(if not for that, actual attempt would have occurred).*<br><br>Overdose: Person has pills in hand but is stopped from ingesting. Once they ingest any pills, this becomes an attempt rather than an interrupted attempt. Shooting: Person has gun pointed toward self, gun is taken away by someone else, or is somehow prevented from pulling trigger. Once they pull the trigger, even if the gun fails to fire, it is an attempt. Jumping: Person is poised to jump, is grabbed and taken down from ledge. Hanging: Person has noose around neck but has not yet started to hang - is stopped from doing so.<br><br>***Has there been a time when you started to do something to end your life but someone or something stopped you before you actually did anything?***<br><br>If yes, describe:	**Yes** **No**<br><br>☐ ☐<br><br>Total # of interrupted<br><br>______
**Aborted or Self-Interrupted Attempt:**<br><br>When person begins to take steps toward making a suicide attempt, but stops themselves before they actually have engaged in any self-destructive behavior. Examples are similar to interrupted attempts, except that the individual stops him/herself, instead of being stopped by something else.<br><br>***Has there been a time when you started to do something to try to end your life but you stopped yourself before you actually did anything?***<br><br>If yes, describe:	**Yes** **No**<br><br>☐ ☐<br><br>Total # of<br><br>aborted or self-interrupted<br><br>______

**Preparatory Acts or Behavior:**<br><br>Acts or preparation towards imminently making a suicide attempt. This can include anything beyond a verbalization or thought, such as assembling a specific method (e.g., buying pills, purchasing a gun) or preparing for one's death by suicide (e.g., giving things away, writing a suicide note).<br><br>***Have you taken any steps towards making a suicide attempt or preparing to kill yourself (such as collecting pills, getting a gun, giving valuables away or writing a suicide note)?***<br><br>If yes, describe:	**Yes** **No**<br>☐ ☐<br><br>Total # of preparatory acts<br><br>______
**Suicide:**<br><br>Death by suicide occurred since last assessment.	**Yes** **No**<br>☐ ☐
	Most Lethal Attempt<br><br>Date:
**Actual Lethality/Medical Damage:**<br><br>0. No physical damage or very minor physical damage (e.g., surface scratches).<br><br>1. Minor physical damage (e.g., lethargic speech; first-degree burns; mild bleeding; sprains).<br><br>2. Moderate physical damage; medical attention needed (e.g., conscious but sleepy, somewhat responsive; second-degree burns; bleeding of major vessel).<br><br>3. Moderately severe physical damage; *medical* hospitalization and likely intensive care required (e.g., comatose with reflexes intact; third-degree burns less than 20% of body; extensive blood loss but can recover; major fractures).<br><br>4. Severe physical damage; *medical* hospitalization with intensive care required (e.g., comatose without reflexes; third-degree burns over 20% of body; extensive blood loss with unstable vital signs; major damage to a vital area).<br><br>5. Death	*Enter Code*<br><br>_______
**Potential Lethality: Only Answer if Actual Lethality=0**<br><br>Likely lethality of actual attempt if no medical damage (the following examples, while having no actual medical damage, had potential for very serious lethality: put gun in mouth and pulled the trigger but gun fails to fire so no medical damage; laying on train tracks with oncoming train but pulled away before run over).<br><br>0 = Behavior not likely to result in injury<br><br>1 = Behavior likely to result in injury but not likely to cause death<br><br>2 = Behavior likely to result in death despite available medical care	*Enter Code*<br><br>_______

Appendix K. Geriatric Depression Scale (Short Form)

Geriatric Depression Scale (Short Form)
Self-Rated Version

Patient's Name: ______________________________________ Date: __________________

***Instructions**: Choose the best answer for how you felt over the past week.*

No.	Question	Answer	Score
1.	Are you basically satisfied with your life?	YES / NO	
2.	Have you dropped many of your activities and interests?	YES / NO	
3.	Do you feel that your life is empty?	YES / NO	
4.	Do you often get bored?	YES / NO	
5.	Are you in good spirits most of the time?	YES / NO	
6.	Are you afraid that something bad is going to happen to you?	YES / NO	
7.	Do you feel happy most of the time?	YES / NO	
8.	Do you often feel helpless?	YES / NO	
9.	Do you prefer to stay at home, rather than going out and doing new things?	YES / NO	
10.	Do you feel you have more problems with memory than most people?	YES / NO	
11.	Do you think it is wonderful to be alive?	YES / NO	
12.	Do you feel pretty worthless the way you are now?	YES / NO	
13.	Do you feel full of energy?	YES / NO	
14.	Do you feel that your situation is hopeless?	YES / NO	
15.	Do you think that most people are better off than you are?	YES / NO	
		TOTAL	

(Sheikh & Yesavage, 1986)

Appendix L. The Parkinson Anxiety Scale (PAS)

The Parkinson Anxiety Scale (PAS); English version

A. Persistent anxiety

B.3. Heart palpitations or heart beating fast (not related to physical effort or activity)

- Never
- Rarely
- Sometimes
- [illegible]

C.3. Specific objects or situations (such as flying, heights, spiders or other animals, needles, or blood)

- Never
- Rarely
- Sometimes
- Often
- Nearly always

C. Avoidance behavior

Please mark one circle for each item below

In the past four weeks, to what extent did you fear or avoid the following situations?

C.1. Social situations (where one may be observed, or evaluated by others, such as speaking in public, or talking to unknown people)

- Never
- Rarely
- Sometimes
- Often
- Nearly always

C.2. Public settings (situations from which it may be difficult or embarrassing to escape, such as queues or lines, crowds, bridges, or public transportation)

- Never
- Rarely
- Sometimes
- Often
- Nearly always

Appendix M. RBD-Single-Question Screen

Have you ever been told, or suspected yourself, that you seem to "act out your dreams" while asleep (for example, punching, flailing your arms in the air, making running movements, etc.)?

☐ Yes ☐ No

Appendix N. Red Flags

RED FLAGS

The following is a list of possible signs and symptoms related to transfusion reactions. If you experience any of these please immediately contact one of the research coordinators (Vicki 713-500-7073; 832-265-6329 or Vanessa 612-559-1124) or the PI of the study (713-500-7121):

- Fever or chills (temperature higher than 37.5 °C / 100.9 °F)
- Pain
- Dyspnea (respiratory distress)
- Headache
- Nausea or vomiting
- Abdominal cramps
- Diarrhea
- Cough
- Bleeding
- Changes in blood pressure: Hypo/hypertension
- Rash or hives
- Edema
- Tachycardia (Increase heart rate >100bpm)
- Jaundice (yellow discoloration in skin and eyes)
- Cyanosis (a bluish discoloration of the skin)
- Numbness/ tingling
- Muscular weakness (loss of strength in any extremity)
- Changes in gait
- Any drastic change in your PD symptoms

IF YOU ARE EXPERIENCING AN EMERGENCY, PLEASE CALL 911 OR HEAD FOR THE NEAREST EMERGENCY ROOM.

Symptoms that could be an emergency include:

- Bleeding that cannot be controlled
- A **worsening** symptom such as fever, difficulty breathing, chest discomfort, or severe pain.
- Increasing swelling or hives, especially if involving the face or throat
- Loss of consciousness, even momentary.

Appendix O. Telephone Evaluation

Patient ID: MSCII-PD-_________________________ Date of Phone Call: ____/_____/__________

Contact Information		
Patient Phone:	Alternate Phone Number:	Patient Email:

Please circle the specific time point:

Infusion 1			
3-7 days post, week 1 (After Infusion #1)	21-35 days post, week 3-5 (Before S&C Assessment)	70 days post, week 10 (After S&C Assessment)	Unscheduled

Infusion 2		
3-7 post, week 14 (After Infusion #2)	21-35 day post, week 16-18 (Before S&C Assessment)	161 days post, week 23 (After S&C Assessment)

Infusion 3			
3-7 days post, week 27 (After infusion #3)	Day 238-252 post, week 34-36 (After S&C Assessment 1)	Day 308-329 post, week 44-47 (After S&C Assessment 2)	Day 420-452 post, week 60-66 (After S&C Assessment 3)

Print HX summary page to include with this report

1. General Narrative/Emailed report

2. Follow up of previous AE reported & not resolved, listed below:

3. List New AEs (System/Start date/End date/severity/cause):

Adverse Event	Start Date	Stop Date	Severity	Cause

** Please use source document *Adverse Event Form* to list all adverse events compl
