# Supplementary material for: Allogeneic bone marrow-derived mesenchymal stem cells in the aging kidney: secondary results of a Parkinson’s disease clinical trial
Source: Stem Cell Res Ther. 2025 Sep 24;16:493. doi: 10.1186/s13287-025-04577-y (PMC12461955; doi:10.1186/s13287-025-04577-y)
Supplement: Supplementary file 2 — Additional file 2. [file 13287_2025_4577_MOESM2_ESM.pdf]

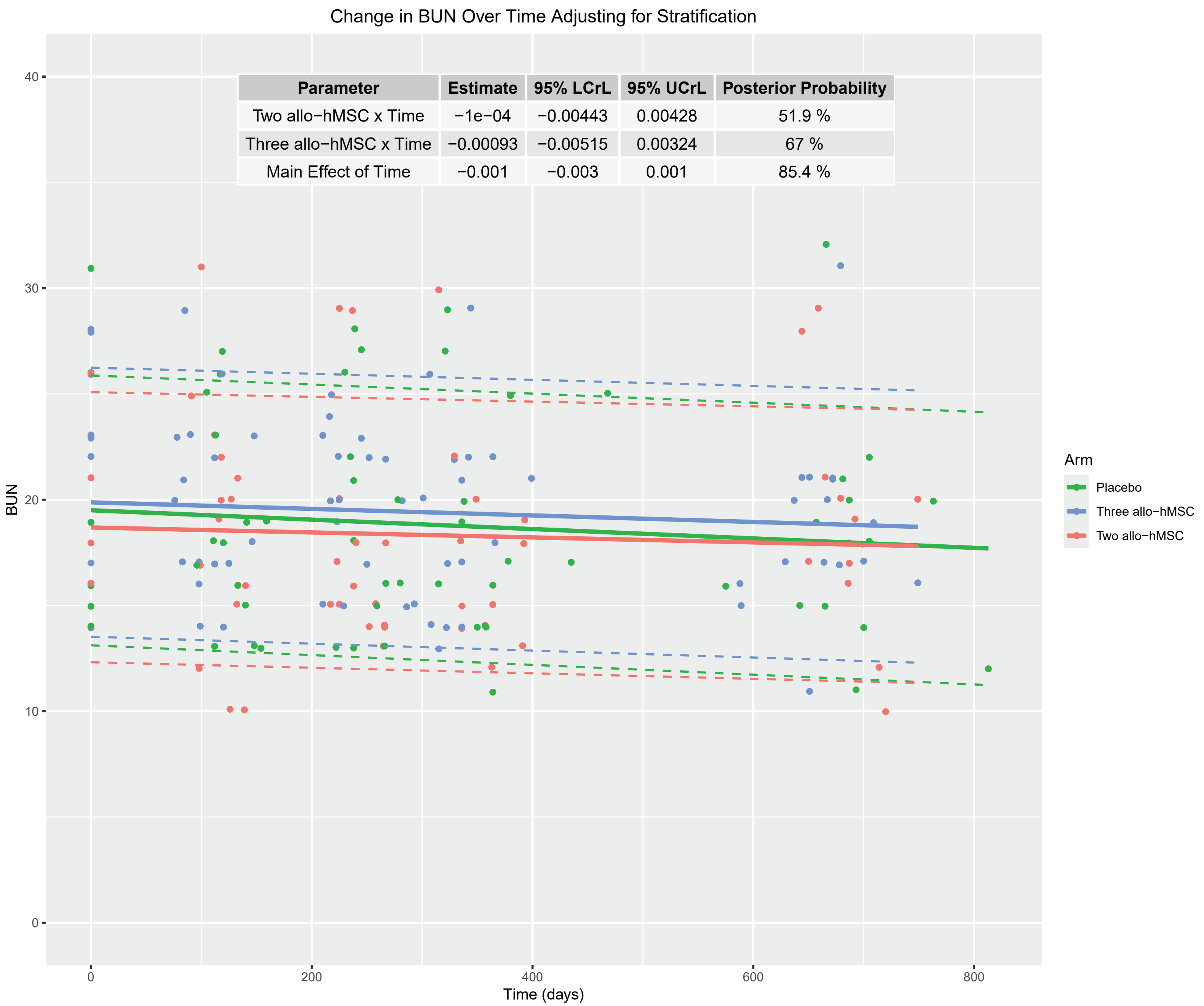

Supplement 2A. Generalized linear modeling of the daily rate of change in BUN levels between the active treatment arms and placebo. Bayesian modeling was used to estimate the daily rate of change, with credible intervals (CrI) and posterior probabilities (PP) reported to quantify the certainty of the observed effects. A posterior probability (PP) of 50–70% indicates weak certainty, 70–90% moderate certainty, 90–95% strong certainty, and >95% very strong certainty.

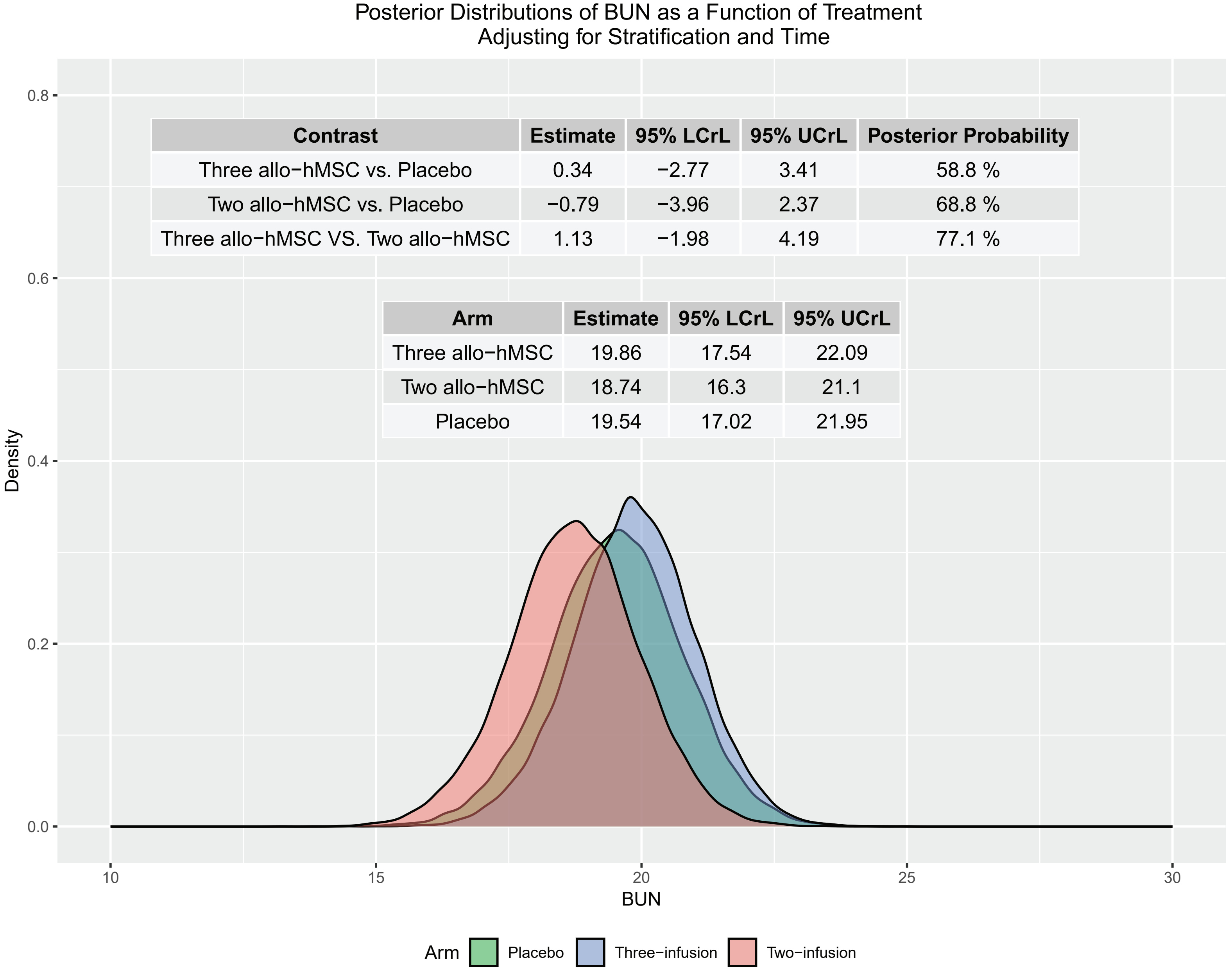

Contrast	Estimate	95% LCrL	95% UCrL	Posterior Probability
Three allo−hMSC vs. Placebo	0.34	−2.77	3.41	58.8 %
Two allo−hMSC vs. Placebo	−0.79	−3.96	2.37	68.8 %
Three allo−hMSC VS. Two allo−hMSC	1.13	−1.98	4.19	77.1 %

Arm	Estimate	95% LCrL	95% UCrL
Three allo−hMSC	19.86	17.54	22.09
Two allo−hMSC	18.74	16.3	21.1
Placebo	19.54	17.02	21.95

Supplement 2B. Comparison of the average serum BUN levels across all timepoints between the treatment arms and placebo. Bayesian modeling was used to estimate the daily rate of change, with credible intervals (CrI) and posterior probabilities (PP) reported to quantify the certainty of the observed effects. A posterior probability (PP) of 50–70% indicates weak certainty, 70–90% moderate certainty, 90–95% strong certainty, and >95% very strong certainty.
